# Transcriptomes and Proteomes Define Gene Expression Progression in Pre-meiotic Maize Anthers

**DOI:** 10.1534/g3.113.009738

**Published:** 2014-06-01

**Authors:** Han Zhang, Rachel L. Egger, Timothy Kelliher, Darren Morrow, John Fernandes, Guo-Ling Nan, Virginia Walbot

**Affiliations:** Department of Biology, Stanford University, Stanford, California 94305-5020

**Keywords:** archesporial cell, cell fate specification, Multiple archesporial cells 1, mac1, pre-meiotic development, genetics of sex

## Abstract

Plants lack a germ line; consequently, during reproduction adult somatic cells within flowers must switch from mitotic proliferation to meiosis. In maize (*Zea mays* L.) anthers, hypoxic conditions in the developing tassel trigger pre-meiotic competence in the column of pluripotent progenitor cells in the center of anther lobes, and within 24 hr these newly specified germinal cells have patterned their surrounding neighbors to differentiate as the first somatic niche cells. Transcriptomes were analyzed by microarray hybridization in carefully staged whole anthers during initial specification events, after the separation of germinal and somatic lineages, during the subsequent rapid mitotic proliferation phase, and during final pre-meiotic germinal and somatic cell differentiation. Maize anthers exhibit a highly complex transcriptome constituting nearly three-quarters of annotated maize genes, and expression patterns are dynamic. Laser microdissection was applied to begin assigning transcripts to tissue and cell types and for comparison to transcriptomes of mutants defective in cell fate specification. Whole anther proteomes were analyzed at three developmental stages by mass spectrometric peptide sequencing using size-fractionated proteins to evaluate the timing of protein accumulation relative to transcript abundance. New insights include early and sustained expression of meiosis-associated genes (77.5% of well-annotated meiosis genes are constitutively active in 0.15 mm anthers), an extremely large change in transcript abundances and types a few days before meiosis (including a class of 1340 transcripts absent specifically at 0.4 mm), and the relative disparity between transcript abundance and protein abundance at any one developmental stage (based on 1303 protein-to-transcript comparisons).

The plant life cycle exhibits an alternation of generations: haploid gametophytes contain some cells that differentiate as gametes and the diploid sporophyte contains the megasporocytes and microsporocytes that undergo meiosis to generate the haploid phase founder cells. Unlike animal embryogeny, during which a germ line of cells competent for meiosis is established (Extavour and Akam 2003), plant sporophytes are entirely somatic until reproduction (Goldberg *et al.* 1993). In angiosperms, age and environmental cues trigger the switch of a somatic shoot apical meristem to an inflorescence meristem that can generate flowers rather than stems and leaves. Flowers are almost entirely composed of somatic cells; within the central-most organ (carpel) and adjacent organ whorl (stamens), a few archesporial (AR) cells differentiate into meiocytes (Hord and Ma 2007). Meiosis generates haploid founder cells and then mitotic cell divisions in gametophytes ultimately produce an egg cell in each embryo sac and two sperm nuclei in each pollen grain (Batygina 2005; Boavida *et al.* 2005; Feng and Dickinson 2007; Feng *et al.* 2013). Fusion of an egg and one sperm cell during fertilization restores the diploid state in the embryo, completing the life cycle (Goldberg *et al.* 1993). Most of the world’s food supply depends on these processes as gamete fertilization initiates seed development encased within the maturing fruit; together, fruits and harvested mature seeds are our most important food sources. Modern agriculture also increasingly relies on hybrid seeds, combining favorable traits from two distinct parents for higher yield and plant vigor. Suppressing male fertility in one parent to permit facile crossing by pollen from the second partner is the key technology in hybrid seed production (Scott *et al.* 2004). Fundamental insight into male reproduction can thus result in agricultural innovations in the multi-billion dollar hybrid seed industry.

Multiple transcriptomes and several proteomes have been reported for whole anthers, utilizing organs that have completed cell specification and are proceeding through meiosis (Lu *et al.* 2006; Ma *et al.* 2007; Wijeratne *et al.* 2007; Skibbe *et al.* 2009; Wang *et al.* 2010a; Aya *et al.* 2011; Deveshwar *et al.* 2011; Nan *et al.* 2011) and for anther developmental stages after meiosis (Ma *et al.* 2008). Using manual or laser capture microdissection, transcriptomes are also available for isolated meiotic plant cells (Tang *et al.* 2010; Schmidt *et al.* 2011; Yang *et al.* 2011) and for isolated sporophytic anther tissues such as the tapetum (Hobo *et al.* 2008; Huang *et al.* 2009). Mature pollen, containing the vegetative cell and sperm, has also been characterized from multiple species (Honys and Twell 2004; Ma *et al.* 2006, 2008; Calarco *et al.* 2012). Collectively, these data provide rich resources for bioinformatic analysis of anther transcriptome progression during meiosis and gametophytic development.

Sample staging and anther dissection during the earlier cell fate specification period are difficult in most flowering plants because typical inflorescences such as those of the model species *Arabidopsis thaliana* and *Oryza sativa* (rice) contain a series of immature perfect flowers at successive stages. Consequently, each flower contains anthers at a different stage and contains both carpels and stamens. Additionally, anthers of most species are very small (<0.05 mm in length) during cell type specification, and the patterning events required to build the layered somatic tissues occur in rapid succession. Given these limitations, initial anther developmental stages in rice and *A. thaliana* have been approached by analysis of whole flowers, including genetic ablation of the carpel to allow anther enrichment (Wellmer *et al.* 2004). These whole flower studies, followed by *in situ* hybridization to localize specific transcripts (Schiefthaler *et al.* 1999) and evaluation of mutants with defects in cell fate specification, provide a glimpse into the complexity of gene regulation during early anther development (Ma 2005; Feng *et al.* 2013).

Maize (*Zea mays*) offers five distinct advantages for analysis of isolated anthers: because maize is monoecious, in the tassel carpels abort early, resulting in male-only tassel flowers; large cohorts of flowers are at the same stage in each tassel; development is highly regular with landmark events and chronology corresponding to anther length (Kelliher and Walbot 2011); maize anthers are relatively large (0.2–0.7 mm) during anther patterning, which facilitates manual dissection; and there are temporal delays between specification events. The longer duration of early stages and intercalation of several-day cell proliferation periods between the completion of successive somatic cell fate specification events account for the increased size of maize anthers compared to *A. thaliana* or rice and makes maize anther stages easier to recognize and dissect (Kelliher and Walbot 2011). For a size comparison between maize and *A. thaliana* over the course of pre-meiotic anther development, see Supporting Information, Table S1 (Smyth *et al*. 1990, Sanders *et al*. 1999).

Maize anther development requires approximately 30 days from the formation of the stamen primordium through pollen shed. We previously profiled gene expression in six anther stages starting at day 5 (1.0 mm anther length), day 8 (1.5 mm, start of prophase I of meiosis), day 9 (2.0 mm, pachytene), day 15 (2.5 mm, tetrad), day 20 (3.0 mm, uninucleate gametophyte), day 30 (4.0 mm, binucleate gametophyte), and day 33 (mature, tricellular pollen) (Ma *et al.* 2008). Anther gene expression is very complex, encompassing ∼75% of the maize protein–coding genes, and highly dynamic with many stage-specific transcripts. The intricacy of the transcriptomes and utilization of anther-specific programs support striking conclusions from genetic analysis: defects in hundreds of genes confer male sterility in maize and other flowering plants (Ma 2005), and male sterility is at least 10-fold more common than female sterility (Timofejeva *et al.* 2013).

To rectify the absence of comprehensive data on early anther development, we isolated maize anthers after initial cell fate specification events, during the mitotic proliferative periods, and during preparation for meiosis. Transcriptome profiling of these whole anther stages combined with cell types isolated by laser capture microdissection permitted elucidation of patterns of gene expression and likely cell type marker transcripts. Stage markers and patterns were tested by quantitative real-time PCR (qRT-PCR), and RNA *in situ* hybridization was used to evaluate a few presumptive cell type specific transcripts. Additionally, *multiple archesporial cells 1* (*mac1*) mutant anthers, which contain excess meiotically competent AR cells but lack normal subepidermal somatic cell types, were evaluated to gain further insight into the hierarchy of genetic programs generating initial cell types. To evaluate the temporal link between RNA expression and protein production, we established proteomes for three stages after 1-D gel fractionation into 11 size classes, isobaric protein sample labeling, and MS/MS detection. The transcriptomes and proteomes allow us to address: (1) the distinctiveness of each stage of anther progression; (2) the reuse of developmental modules at different stages; (3) the possibility that anthers store mRNA without translation; and (4) the likely cell type specific processes facilitating cell type specification and initial cell differentiation in maize anthers.

## Materials and Methods

### Transcriptome analysis

#### Plant samples:

Except as noted, all anthers were collected from the W23 *bz2* (defect in vacuolar anthocyanin sequestration) inbred maize line. Anthers were dissected and staged by length using a micrometer. Anthers of the target length (±0.03 mm) were pooled from one tassel to generate a biological replicate for each size class. Cell counts of anthers at successive stages were obtained from confocal images of entire anther lobes. Images were collected using a Leica SP5 confocal microscope after propidium iodide staining as outlined by Kelliher and Walbot (2011). A representative view was chosen near the center of each scanned anther, and the numbers and dimensions of each cell type were measured using Volocity software (PerkinElmer, Waltham, MA). The cell counts were extrapolated to estimate the total numbers for that organ by scaling to whole anther length. A minimum of three anthers of each length was evaluated. Volumes were measured with the line tool in Volocity using an average of the three dimensions (X, Y, Z) for each of three different cells for each cell type. Anthers of similar length were then classified into size bins and average values were calculated for both cell number and cell volume within those bins; each bin contained a minimum of four individually analyzed anthers. The results obtained with these methods are very similar to those reported by Kelliher and Walbot (2011), with a slight change to counting procedure and the addition of several younger stages.

#### RNA extraction, cDNA preparation, and qRT-PCR:

Total RNA from pools of staged, whole anthers was extracted using the Trizol-Plus procedure (Life Technologies, Grand Island, NY). DNase-treated RNA samples were evaluated on the 2010 Agilent Bioanalyzer (Santa Clara, CA) before being reverse-transcribed by the SuperScript III First-Strand Synthesis System (Life Technologies). The qRT-PCR data were obtained on an Opticon 2 (BioRad, Hercules, CA) using primers designed by primer3 software (Untergasser *et al.* 2012); primers are listed in Table S4. Data were analyzed using PCR Miner software (Zhao and Fernald 2005). For laser microdissected samples, 0.7 mm anthers from four plants were fixed, sectioned, and laser-microdissected to recover cell types as described previously (Kelliher and Walbot 2012).

#### RNA in situ hybridization:

Tassels were vacuum-infiltrated on ice in 50% ethanol, 3.7% formaldehyde, 5% glacial acetic acid, 0.5% Triton X-100, and 1% DMSO, and then fixed overnight. Tassels were dehydrated through an ethanol series, cleared with Histoclear (National Diagnostics, Atlanta, GA), and then embedded in Paraplast Plus (Sigma-Aldrich, St, Louis, MO) following the protocols of Jackson *et al.* (1994). Hybridizations were performed with probes transcribed using the DIG RNA Labeling Kit (T7/SP6) (Roche, http://www.roche-applied-science.com). Sense and antisense probes were synthesized from PCR fragments amplified from cDNA clones obtained from the University of Arizona maize cDNA collection (http://maizecdna.org/). Probes were produced using T7 and SP6 polymerases directly from the cDNA clone; no gene-specific primers were used. RNA *in situ* hybridizations were performed on tassel sections containing anthers from 0.15 to 1 mm, as described previously (Schiefthaler *et al.* 1999). Estimation of anther length from *in situ* images is based on tissue and cellular morphology.

#### Microarray hybridization:

cDNA samples were amplified and labeled; the resulting cRNA was hybridized to a 4x44K format two-color microarray (Agilent Part number: G2519F; Design ID: 016047) in a balanced dye swap design as described previously (Kelliher and Walbot 2012). The samples were arranged into three experimental loops. The first loop compared wild-type 0.15 mm, 0.25 mm, 0.4 mm, and 0.4 mm *mac1* (introgressed into the W23 inbred for five generations) anthers. In the second loop, wild-type 0.7 mm whole anther, AR cells, and inner somatic layers (middle layer and tapetum) were compared, with the outer somatic layers (epidermis and endothecium) to whole anther comparison as an out group. The third loop compared 0.2 mm wild-type with 0.2 mm *mac1*. The 1.0 mm wild-type data were from a separate experiment but used the same array and sample preparation procedures. Typically, a minimum of two replicates and up to four biological replicates were used. In cases in which two biological replicates were utilized, each was hybridized in duplicate for a total of four hybridization assessments.

#### Data analysis:

Slides were scanned and data were processed as described previously (Nan *et al.* 2011). The resulting median foreground values for the red and green channels were normalized in two steps using the LIMMA package in R as follows: “within arrays” using the LOWESS method and “between arrays” using the quartile method. Probes with expression values more than 3.0 SD above the average foreground of the array’s negative controls were considered ON, resulting in an estimated false detection rate of 0.13%. The small number of probes with <75% of the biological replicate measurements scored as ON were excluded from further analysis. Significance for differential expression was set at 1.5-fold (log2 ± 0.58) with a p-value ≤ 0.05 for evaluation of data within a single experimental loop.

#### Metabolic pathways analysis:

Transcripts constitutively expressed in whole anthers from the 0.15 to 1.0 mm stages were selected and log2 ratios of average intensities between each pair of consecutive stages were mapped in the Pathway Tools Omic Viewer (SRI, Menlo Park, CA) using the MaizeCyc database version 2.1 (http://pathway.gramene.org/gramene/maizecyc.shtml). The list of pathways with differentially expressed genes was extracted using a two-fold cutoff for significance. The average intensities were extracted for all the constitutively expressed transcripts belonging to the hormone-associated pathways identified with at least one transcript meeting the two-fold cutoff. For the laser-microdissected cell type data, we identified pathways *ad hoc* that had high transcript enrichments in at least one tassel lobe zone and then calculated the percentage of differentially regulated genes for each sample.

### Proteome analysis

#### Anther samples and isobaric labeling:

Duplicate biological replicate samples of 0.4, 0.7, and 1.0 mm wild-type anthers were collected. Total protein was extracted as described by Wang *et al.* (2012a) and Casati *et al.* (2005) and quantified using a Bradford assay. Samples of 25 μg were loaded onto NuPAGE Novex Bis-Tris Precast gel cassette acrylamide gels (Life Technologies) along with 10 μl Novex Sharp Standard size standard (Life Technologies) in a separate lane and electrophoresed for 35 min at constant 200 V. Using a methanol-cleaned scalpel, 11 size-fractionated samples were collected from each lane (3.5–10 kD, 10–15 kD, 15–20 kD, 20–30 kD, 30–40 kD, 40–50 kD, 50–60 kD, 60–80 kD, 80–110 kD, 110–160 kD, 160–260 kD). The three lowest molecular weight fractions were digested in-gel using LysC (Promega, Madison, WI) as described by Wang *et al.* (2012a) and the other fractions were digested with Trypsin/LysC (Promega). After peptide extraction, each pool was dried to completion, after which peptides were labeled with amine-reactive tags (TMT) (Thermo Scientific, Rockford, IL) in a sixplex fashion with the following assignments: 0.4 mm replicate one, isobaric tag 130; 0.4 mm replicate two, isobaric tag 131; 0.7 mm replicate one, isobaric tag 126; 0.7 mm replicate two, isobaric tag 127; 1.0 mm replicate one, isobaric tag 128; and 1.0 mm replicate two, isobaric tag 129. After isobaric tagging, each fraction was pooled 1:1 and tested in a pilot analysis; the pilot assays were used to devise appropriate multiplex pooling mixtures.

#### Peptide sequencing:

The peptide pool of each representative fraction was then loaded on a nano-Acquity UPLC (Waters, Milford, MA) using a self-packed nano-UPLC column 25 cm in length with an internal diameter of 100 µM and a C18 matrix. Gradient lengths were 120 min for each fraction and the source was a Proxeon source directly in front of a LTQ Orbitrap Velos mass spectrometer (Thermo Fisher, San Jose, CA). The data acquisition was such that the survey scan was performed in the orbitrap mass analyzer at a resolution of 60,000 with 750,000 ions, and the MS/MS was performed using HCD for the top eight most intense precursor ions where the MSn target ion value was 75,000. RAW data were converted to mzXML format and searched on a Sorcerer platform using a project-generated maize database incorporating all six potential reading frames for each gene. Using a reverse decoy database approach, the dataset was then filtered to a 1% false discovery rate (FDR); approximately 2000 proteins were identified with high confidence. Using a virtual translation of all maize genes from the project-generated protein database, peptides were matched to the genome using Scaffold4 software (Proteome Software Inc., Portland, OR).

#### Data analysis:

To assess protein abundances quantitatively, only the constitutively present proteins were analyzed; this procedure is required from our experimental design in which each sample type (containing a particular isobaric tag) can be set as the reference for a comparison, but the software can only analyze quantitative values using a ratiometric algorithm. Therefore, proteins absent in any sample are excluded from the analysis and no analysis of stage-specific proteins is available. In total “change” proteins were identified at FDR of 1% and normalized for quantitative analysis using the Scaffold4 software package using log2 values to compare between sample types. Approximately 80% of the proteins identified by mass spectrometry matched an annotated maize gene; for ∼67% of these, there was a probe on the 4x44K Agilent microarrays, resulting in a total of 1303 protein-to-RNA comparisons.

## Results

### Anther ontogeny

**Figure 1:**
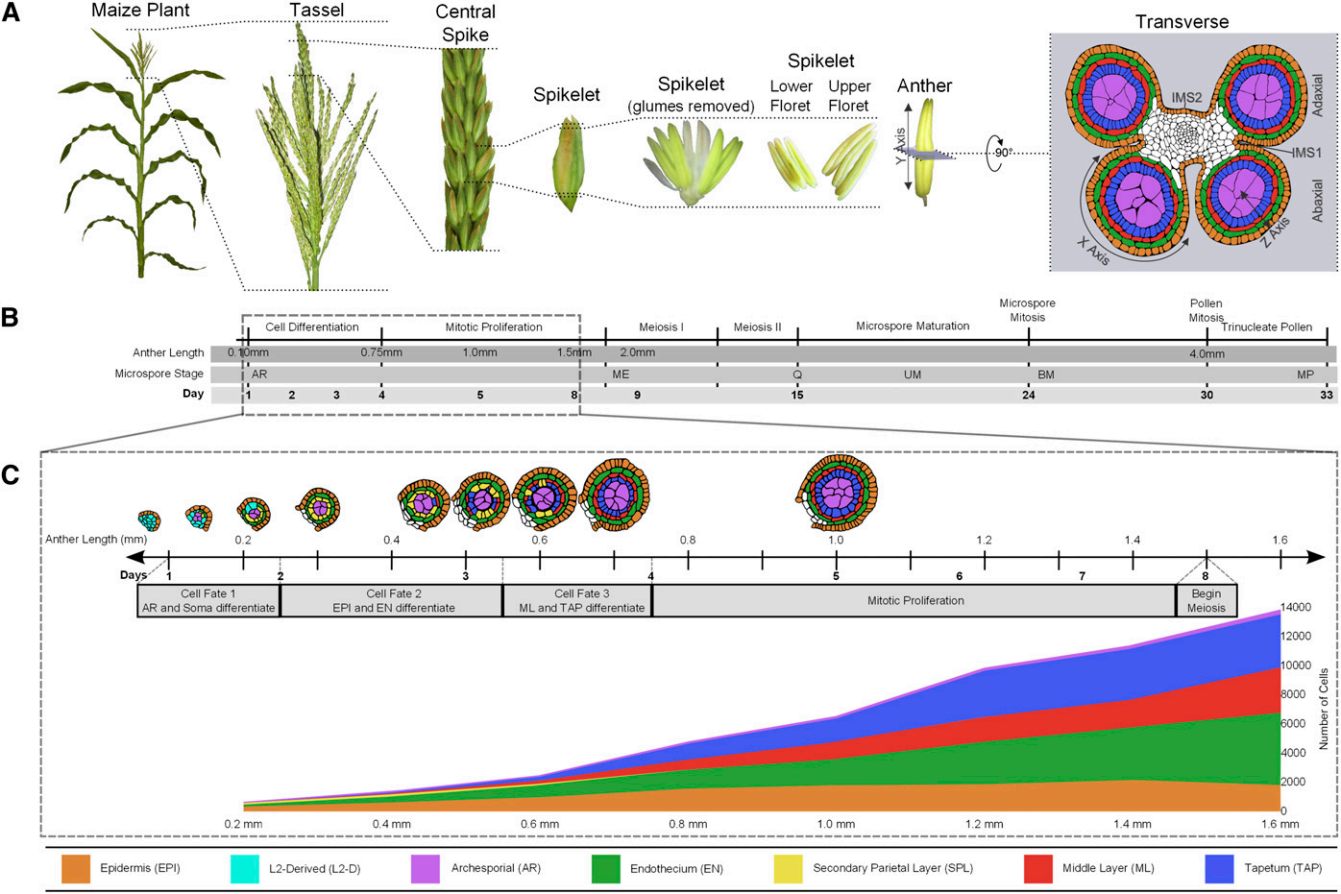
Morphology, anatomical terms, and staging of maize anthers. (A) Major structures of a maize plant relevant to understanding anther ontogeny. The final panel also indicates the X-, Y-, and Z-axis designations used in confocal microscopic analysis of anther cells. The Y-axis is the length dimension used as a proxy for anther developmental stage. Abaxial and adaxial lobes of the anther are indicated in the transverse view, as are the intermicrosporangial stripes (IMS) 1 and 2 that separate the lobes. A color code for the anther lobe cell types is provided at the bottom of the figure. (B) Complete timeline of maize anther development from organ identity specification through pollen shed. The dotted box outlines the period corresponding to (C). Descriptors are provided for the key events in anther development across the entire timeline. The microspore stages are abbreviated: archesporial cells (AR); meiocytes (ME); quartet stage of microspores (Q); uninucleate microspore (UM); binucleate microspore (BM); and mature pollen (MP). (C) Tracings of anther lobes in transverse section after anther staging by length with the typical number of days of development since anther primordium inception are also indicated. Given the variable environment of plant growth in the field and greenhouse, days are approximate, but anther lengths are tightly correlated with developmental stage. The gray bar provides a guide to the key developmental events and the span of each event. The graph depicts the increase in cell numbers from 0.2 mm to 1.6 mm (germinal cell specification through entry into meiosis). Not included in the graph are the vasculature and connective tissue cells in the center of each anther, which constitute ∼10% of all cells.

Anthers are contained within the tassel, the terminal inflorescence at the top of the maize plant (Figure 1A); each tassel produces hundreds of spikelets, each containing two flowers (florets). Because large cohorts of florets are at the same developmental stage within a tassel, recovery of carefully staged anthers for microscopic or molecular analysis is straightforward. Similar to most flowering plants, maize initially produces perfect flowers with both reproductive organs; however, the stamens abort in the ear and the carpels abort in the tassel. Consequently, each tassel floret has three stamens but no carpel. Within a spikelet, the upper floret stamens develop approximately 1 day ahead of the lower floret. Each stamen consists of a slender filament connecting the terminal anther to the vegetative plant. At the initiation of meiosis, each anther consists of four equivalent lobes containing four rings of somatic cells encircling the meiocytes.

As shown in Figure 1B, an anther lobe primordium consists of only Layer1-derived (L1-d; presumptive epidermis) and Layer2-derived (L2-d; internal tissues) cells. Subsequent anatomical development is charted against anther length because this metric shows excellent correspondence to internal stages in maize anthers (Kelliher and Walbot 2011). The first step in anther patterning is AR specification; in maize, this event requires a redox switch mediated by the MSCA1 glutaredoxin (Kelliher and Walbot 2012). This process starts just after the 0.15 mm stage and a full column of AR cells is present in each anther lobe by 0.25 mm. The newly specified AR secrete MAC1, a dual-function ligand that suppresses AR proliferation but activates the single layer sheath of neighboring pluripotent L2-d cells to divide periclinally, a process that initiates while the AR column is still forming and is completed by 0.3 mm. Sister cells in the newly formed somatic bilayer acquire distinctive fates and cell shapes by the 0.35 mm stage: the subepidermal cells become endothecium (EN) and those adjacent to the AR become bipotent secondary parietal layer (SPL) cells (Kelliher and Walbot 2012; Wang *et al.* 2012b). After 3 days of within-layer proliferation, the final cell specification event is completed by 0.7 mm after the periclinal division of each SPL cell generates two daughters, a middle layer (ML) and tapetal (TAP) cell; the resulting lobes contain five tissues each composed of one cell type and arranged in a dartboard architecture (Kelliher and Walbot 2011).

Over the subsequent several days, rapid anticlinal cell division in all somatic cell types results in a longer and wider anther by the 1.0 mm stage (Figure 1C). Each cell type has a characteristic cell shape and volume and the numbers of cells in each tissue are highly reproducible (Kelliher and Walbot 2011; Figure 1C). After the 1.2 mm stage, division slows in all somatic cell types. From their inception the AR are the largest cells in the anther lobe; although specified first, their mitotic pace is much slower than the somatic cell types. From the initial column of two to five AR in each 0.2 mm anther lobe, a final number of ∼160 AR per lobe, haphazardly arranged in four columns each with ∼40 AR, is reached by 1.0 mm. AR mitosis finishes at 1.0 mm; over the subsequent several days these cells expand further and differentiate as pollen mother cells (PMC) in preparation for meiosis. In W23, the PMC are in prophase I by the 1.5 mm stage, by 2.0 mm in other inbreds, and meiotic stages are synchronous among all PMC in an anther over the following 6 days (Figure 1C). All four meiotic products of a normal meiosis are viable, and their post-meiotic development involves two haploid mitotic divisions to generate the trinucleate pollen grain, containing two sperm nuclei encompassed within a large vegetative cell.

### Transcriptome profiling of staged anthers

Three key stages were selected for transcriptome profiling: 0.4 mm during a period of rapid clonal proliferation after the generation of EN and SPL completes the second cell fate specification step; 0.7 mm after the SPL divisions complete the third cell fate specification event forming the ML and TAP; and 1.0 mm when AR mitotic divisions finish and the somatic and germinal cells have differentiated into stereotypic shapes and volumes (Table S2). For comparison to the earliest steps in primordium differentiation, two earlier anther stages were included in the 0.4 mm experimental loop: 0.15 mm anthers prior to differentiation of the L2-d cells into AR and somatic tissues and 0.25 mm anthers in which AR occupy a column from lobe tip to base and the EN and SPL cells are being generated by periclinal division of the L2-d cells adjacent to the AR (Kelliher and Walbot 2014). Analysis of numerous maize male-sterile mutants indicates that defects in somatic cell fate specification prior to 0.4 mm as well as at the 0.4 and 0.7 mm stages result in meiotic arrest (Timofejeva *et al.* 2013).

**Figure 2:**
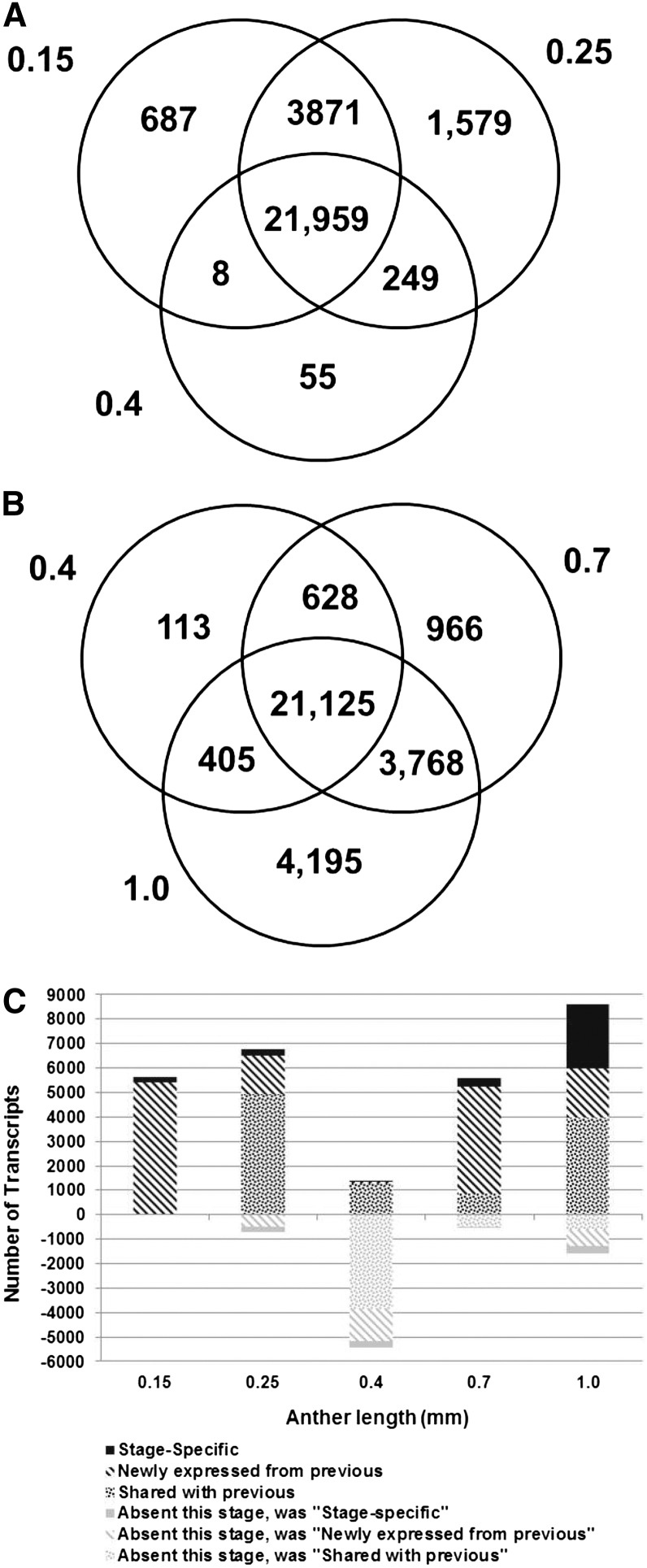
Major patterns of transcript expression in immature maize anthers. (A) Overlap of ON expression between stages 0.15 mm, 0.25 mm, and 0.4 mm. (B) Overlap of ON expression between stages 0.4 mm, 0.7 mm, and 1.0 mm. (C) Graphical analysis of transcript gain and loss from the 0.15 mm to 1 mm stage. Counts above zero represent transcripts present in that stage classified by their relation to the previous stage (newly expressed or shared with previous stage), but stage-specific transcripts are separately categorized. Counts below zero indicate transcripts that were present in the previous stage but were not detected in the current stage. The previous category of these transcripts is also indicated by the similar but lighter color pattern.

Table S2 reports two transcriptome data analyses. The Venn diagrams in Figure 2, A and B illustrate that at the early stages of anther development from 0.15 to 1.0 mm the transcriptome is very complex, with 21,959 transcripts commonly expressed among the 0.15 to 0.4 mm stages and 21,125 common transcripts among the 0.4 to 1.0 mm stages, plus numerous stage-specific or stage-restricted transcripts. With the exception of the 0.4 mm stage, there is considerable overlap in transcript accumulation between contiguous stages. The distinctiveness of the 0.4 mm stage is highlighted in Figure 2C, which quantifies the major classes of transcript gain or loss over the developmental progression. The number of stage-specific transcripts generally increases with developmental stage: 0.15 mm (N = 208, pluripotent cells present), 0.25 mm (N = 259, AR present and EN and SPL forming), 0.7 mm (N = 327, all lobe cell types are present), and 1.0 mm (N = 2614, when cell differentiation is advanced). The 0.4 mm stage after the three-layered somatic niche is established contains surprisingly few (11) stage-specific transcripts; this may reflect the persistence of transcripts from the previous stage and the absence of cell type specific differentiation transcripts, many of which are likely expressed after this stage.

**Figure 3:**
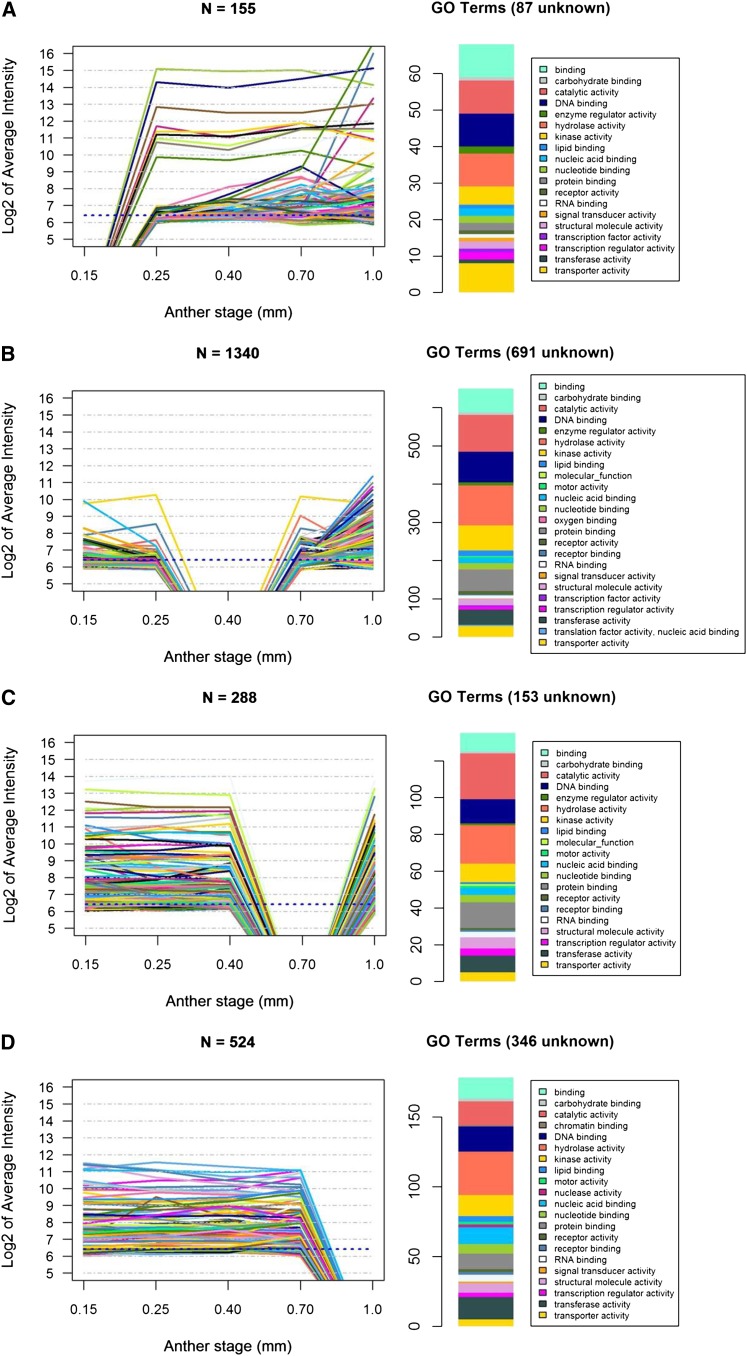
Microarray intensities for four clusters of genes with detectable transcript in all but one stage. Clusters of transcripts shown are those not detected only at 0.15 mm (A), 0.4 mm (B), 0.7 mm (C), or 1.0 mm (D). To the right of each expression pattern is a histogram of the GO term counts associated with the transcripts after removal of any transcripts lacking GO terms.

Each developmental stage shares many transcripts with the previous stage, with a low proportion of transcript disappearance (negative numbers below zero) (Figure 2C), except at 0.4 mm. At this developmental stage when one L2-d cell layer (the SPL) is still bipotent, there are only 11 stage-specific transcripts and thousands of transcripts expressed at 0.25 mm are no longer detectable by microarray hybridization (Figure 2C). This “trough” pattern at 0.4 mm is just one example of gene cohorts turned ON or OFF at key stages of development, but it is of particular interest biologically because of the lack of cell specification events at this stage and the magnitude of the OFF class. In Figure 3, the microarray intensity levels are shown for transcripts exhibiting the trough pattern at four of the five stages studied (all but 0.25 mm). For each pattern, a large number of the intensities before or after the trough are above the median level for the experiment (indicated with a dotted line): 39 of 155 at 0.15 mm; 551 of 1340 at 0.4 mm; 263 of 288 at 0.7 mm; and 422 of 524 at 1.0 mm (Figure 3, Table S3). Gene Ontology (GO) terms were used to classify these transcripts into categories. These GO assignments further demonstrate the dynamic nature of the anther transcriptome and the involvement of diverse cellular processes.

To assess transcript classification by microarray hybridization, qRT-PCR was used. Constitutive ON and constitutive OFF patterns were validated for 7/7 and 2/3 tests, respectively. Seven gene transcripts exhibiting the trough pattern at 0.4 mm were examined; only one showed the matching expression pattern and two transcripts were scored as exceedingly rare at 0.4 mm (C_t_ value greater than 31) (Table S4); the other four were scored as present by qRT-PCR. Our array analysis sets a relatively high background threshold (three-times the SD of the non-hybridizing control probes) to maintain an FDR of 0.13%. These criteria mean that probes with very low expression values can be scored as OFF. Furthermore, some array probes may exhibit poor hybridization behavior, resulting in a false negative classification. To reexamine the nature of the trough at 0.4 mm, we evaluated expression of this gene set at the preceding stage. Of the 5450 transcripts missing specifically from the 0.4 mm stage, 5444 (99%) were below the median level of expression at 0.25 mm and 3321 (61%) were in the lowest quartile. Therefore, most transcripts “missing” at 0.4 mm were low or very low abundance transcripts at the previous stage and then decreased below the quantitative cutoff value in microarray hybridization.

Are the transcripts absent at 0.4 mm markers for anther primordia that are permanently downregulated after the first round of differentiation? This does not appear to be the case because most (3523, 65%) are re-expressed in the subsequent 0.7 mm anther stage (Figure 3B). Some of the missing transcripts may represent genes responsible for executing layer-adding periclinal divisions and/or cell fate establishment. These developmental events are prevalent in 0.15 mm, 0.25 mm, and 0.7 mm anthers; however, at 0.4 mm, only within-layer anticlinal divisions occur, contributing to anther elongation (L-anticlinal divisions) and increases in girth (G-anticlinal divisions). Future research can address whether the decrease in transcript abundances at 0.4 mm reflects transient transcriptional repression or stage-specific mRNA degradation.

### Proteomics to microarray comparison

To investigate if high-abundance transcripts observed in the microarray analysis would be translated at the same stage, anthers of 0.4 mm, 0.7 mm, and 1.0 mm were collected for protein identification by mass spectrometry. In total, 1967 proteins were identified in all three of these stages with an FDR of 1%. The use of TMT-sixplex isobaric tags allowed for direct, quantitative comparison between sample types. Two technical issues constrained the depth of proteome analysis: duration of the gradients during data acquisition and the use of TMT isobaric tags, which can result in some protein loss during the tagging procedure. Given that the total number of proteins identified is approximately 10% of the constitutively expressed transcripts, we conclude that this proteome analysis will only address abundant proteins.

**Figure 4:**
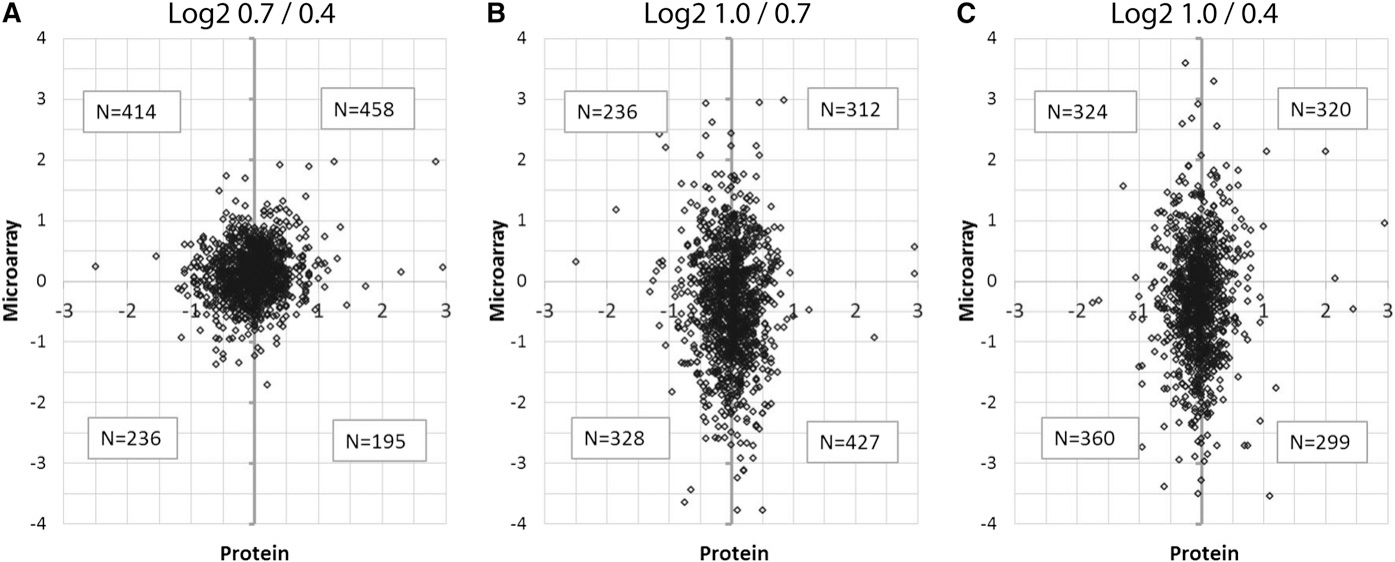
Protein abundance ratios compared to microarray intensity ratios. For the two stages indicated, log2 ratios of the protein abundance are plotted against the ratios of the microarray intensities for the 1303 gene types on both platforms at all three stages. (A) Comparing 0.7 mm to 0.4 mm. (B) Comparing 1.0 mm to 0.7 mm. (C) Comparing 1.0 mm to 0.4 mm. The non-linearity of the relationship between the two datasets emphasizes that a change in transcript abundance between two developmental stages (often days apart) is not necessarily associated with a similar change in the corresponding protein over that same interval.

To evaluate the protein data in relation to the microarray results, log2 values of the 1303 proteins identified for which there was a corresponding gene probe on the microarray were compared in all pairwise combinations of developmental stages (Figure 4). If the hypothesis that large fold-changes in RNA between stages are accompanied by large changes in protein were true, then we would expect a linear relationship when protein values were plotted against the microarray values. Instead, we observe in all three comparisons that the relationship between transcript changes and proteins is much less predictable. This is also true when the RNA and protein data are binned into deciles and then compared (Figure S1). In Figure S1, both protein and microarray abundances are represented as circles scaled to relative abundances of data within each decile of the respective data sets. The relatively uniform distribution of the data agrees with the representation in Figure 4 that transcript abundance is not a good indicator of protein abundance at a given developmental stage.

A previous proteomics data set generated using similar methods analyzed proteins below 20 kD in meiotic anthers at stages 1.5 mm and 2.0 mm (Wang *et al.* 2012a). To determine how dynamic small protein types are, we pooled these size classes (N = 226 proteins) from our 0.4 mm, 0.7 mm, and 1.0 mm datasets to compare to the 424 small proteins identified for the combined 1.5 mm and 2.0 mm stages identified by Wang *et al.* (2012a) (Figure S2). These 424 proteins were identified at the same 1% FDR and peptide confidence levels as the TMT-multiplexed data presented in this study, although more stringent criteria reported in the original Wang *et al.* (2012a) study yielded a list of 359 proteins. These early and later stages are strikingly different: only 19 proteins were found in common between the two data sets for proteins in the size classes between 3.5 and 20 kD (Figure S2). When all the proteins identified in the TMT-tagged proteome were included in the comparison, there were 120 proteins in common with the list of Wang *et al.* (2012a). The low-molecular-weight proteins identified may vary between these data sets because of a reduction in the number of proteins post-translationally cleaved after the onset of meiosis. Overall, the results indicate that the proteome for early anther development is very different from that in meiotic anthers.

### Comparison between stages

**Figure 5:**
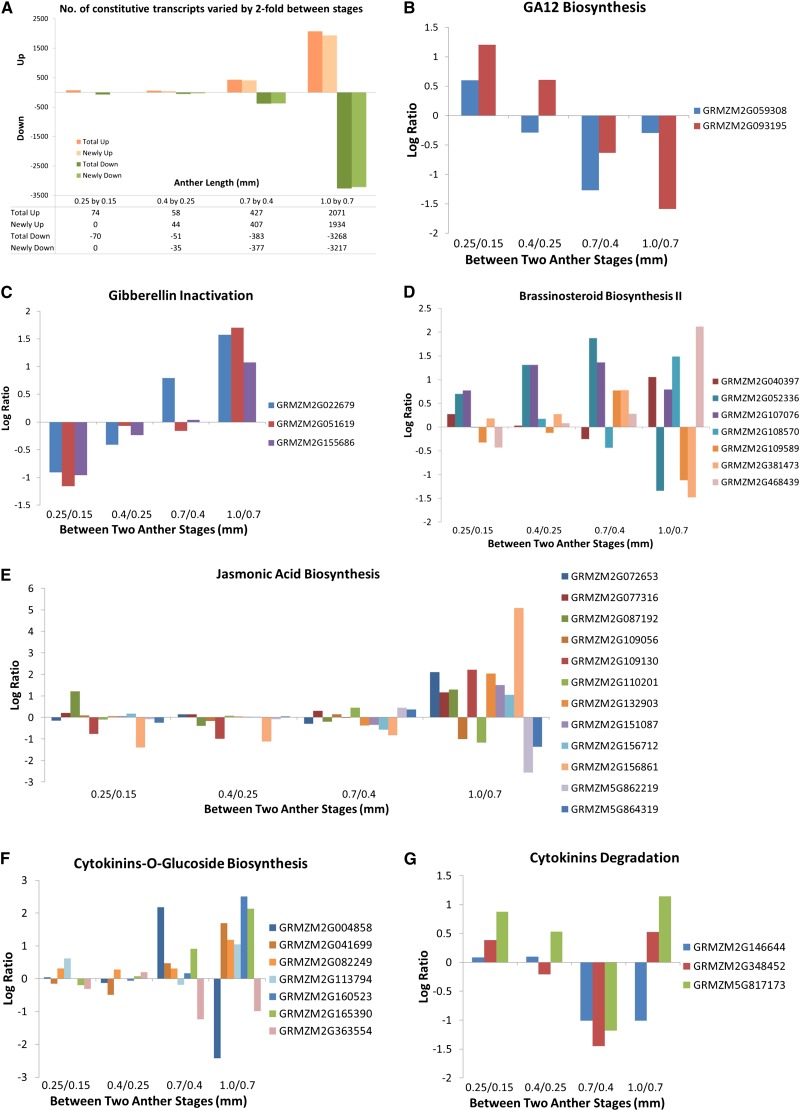
Pathway analysis of constitutive transcripts that are differentially regulated with the significance set at two-fold of normalized average intensities. (A) Number of differentially regulated transcripts compared between two consecutive anther stages: UP, increased by two-fold from the previous stage, and DOWN, decreased by two-fold from previous stage. Newly indicates not found in the previous stage. (B–G) Log2 ratio of differentially expressed transcripts in GA12 biosynthesis (B), gibberellin inactivation (C), brassinosteroid biosynthesis II (D), jasmonic acid biosynthesis (E), cytokinin-glucoside biosynthesis (F), and cytokinin degradation (G) pathways.

In addition to the ON and OFF categories, we also investigated transcripts that are constitutively expressed at all stages (N = 22,257) but are differentially expressed between at least two developmental stages using a two-fold cutoff for significance. Few transcripts met these criteria from 0.15 mm to 0.4 mm followed by a large increase in such differentially regulated transcripts at subsequent stages. There were 144 transcripts (74 UP, 70 DOWN) between 0.15 mm and 0.25 mm and 109 transcripts between 0.25 mm and 0.4 mm (58 UP, 51 DOWN). In transitioning from the 0.4 mm to 0.7 mm stages, 810 transcripts were differentially expressed (427 UP, 383 DOWN); between 0.7 mm and 1.0 mm, 5339 transcripts (2071 UP, 3268 DOWN) were identified (Figure 5A). We hypothesize that the large increase in significantly differentially regulated transcripts at 0.7 mm and 1.0 mm reflects cell differentiation of the EPI, EN, and AR by 0.7 mm and by all five lobe cell types at 1.0 mm.

To gain insight into the types of processes contained within the differentially regulated gene sets, transcripts were mapped to metabolic pathways. The current MaizeCyc database permits evaluation of 4049 genes organized into 479 pathways. All pathways with a two-fold upregulated or downregulated gene were chosen for closer inspection. Pathways well-represented in all four comparisons (0.25/0.15, 0.4/0.25, 0.7/0.4, and 1.0/0.7) include carbohydrate, amino acid, and lipid/fatty acid metabolism (both biosynthesis and degradation). This result indicates that there is ongoing turnover of basic metabolites necessary to support the fast pace of growth and development in immature anthers and that there is unexpectedly dynamic gene regulation of these core biochemical pathways.

Anthers are unique organs because there is no meristem organizing cell production. Organ growth and cellular differentiation are either organized cell-autonomously or through perception of signals delivered through the vascular tissue or generated by one lobe cell type and perceived by another. Hormone production within each anther, with specific hormones playing larger roles at specific stages, is a logical hypothesis to explain growth coordination within anthers (Mutasa-Göttgens and Hedden 2009; Ye *et al.* 2010; Depuydt and Hardtke 2011; Song *et al.* 2013). Although constitutively expressed, a number of hormone-related genes are differentially regulated during pre-meiotic anther development (Figure 5, Table S5). If the gene expression patterns are predictive of hormone levels, then the data indicate that there are substantial changes in hormone biology. Supporting this idea, among the 180 steps analyzed in the hormone-associated pathways, 18 correspond to a protein detected in the proteomics data set (Table S5) indicating that at least some of the steps evaluated are present as proteins. Comparing the 0.15 mm to 0.25 mm stages, two gibberellin A_12_ (GA_12_) biosynthesis gene transcripts (GRMZM2G059308 and GRMZM2G093195) are upregulated (UP) and three gibberellin inactivation transcripts (GRMZM2G022679, GRMZM2G051619, and GRMZM2G155686) are downregulated (DOWN). The opposite pattern is present at the 0.7 mm and 1.0 mm stages (Figure 5, B and C). These observations suggest the hypothesis that GA is involved in cell fate specification events—AR, EN, and SPL patterning—but is less important during anther anticlinal cell proliferation.

In contrast, transcript levels of genes in the brassinosteroid biosynthesis II pathway were UP at both the 0.4 mm stage (GRMZM2G052336 and GRMZM2G107076) and the 1.0 mm stage (GRMZM2G040397, GRMZM2G108570, and GRMZM2G468439) (Figure 5D), whereas the majority in the jasmonic acid biosynthesis pathway (GRMZM2G072653, GRMZM2G077316, GRMZM2G087192, GRMZM2G109130, GRMZM2G132903, GRMZM2G151087, GRMZM2G156712, and GRMZM2G156861) were UP only at the 1.0 mm stage (Figure 5E). These results also suggest that specific hormones may play instructive roles during landmark developmental events.

Genes involved in cytokinin inactivation through formation of cytokinin-O-glucoside (Figure 5F) and cytokinin degradation (Figure 5G) showed complex yet interesting patterns. Two were DOWN at both the 0.7 mm stage and the 1.0 mm stage (cytokinin-O-glucoside biosynthesis: GRMZM2G363554; cytokinin degradation: GRMZM2G146644). Two were DOWN at the 0.7 mm stage and then were re-expressed at the 1.0 mm stage (cytokinin degradation: GRMZM2G348452 and GRMZM5G17173), whereas another one was UP at the 0.7 mm stage (cytokinin-O-glucoside biosynthesis: GRMZM2G004858) and then went DOWN at the 1.0 mm stage. Finally, a total of five genes in the cytokinin-O-glucoside biosynthesis pathway (GRMZM2G041699, GRMZM2G082249, GRMZM2G113794, GRMZM2G160523, and GRMZM135390) were UP only at the 1.0 mm stage. Collectively, these data suggest that cytokinin degradation and sequestration may be important in the modulation of cell division pace and hint at cell type specificity: AR divisions stop by 1.0 mm and the pace of somatic cell mitosis slows after 1.2 mm (Figure 1C).

Because the tassel develops within a whorl of immature leaves and maize anthers are encased in several outer organs, anthers grow in darkness during the developmental stages considered here. Therefore, it was surprising to find that anthers express genes involved in photosynthesis starting at the 0.7 mm stage. Examples include photosynthesis light reaction components (GRMZM2G080107 and GRMZM2G168143), Calvin-Benson-Bassham cycle enzymes (*Ssu1*-GRMZM2G098520 and *Gpa1*-GRMZM2G337113), CO_2_ fixation (*Pep1*-GRMZM2G083841), and chlorophyllide, a biosynthesis factor (GRMZM2G084958 and GRMZM2G073351).

### Transcript profiling of transcription factors and meiotic genes

**Figure 6:**
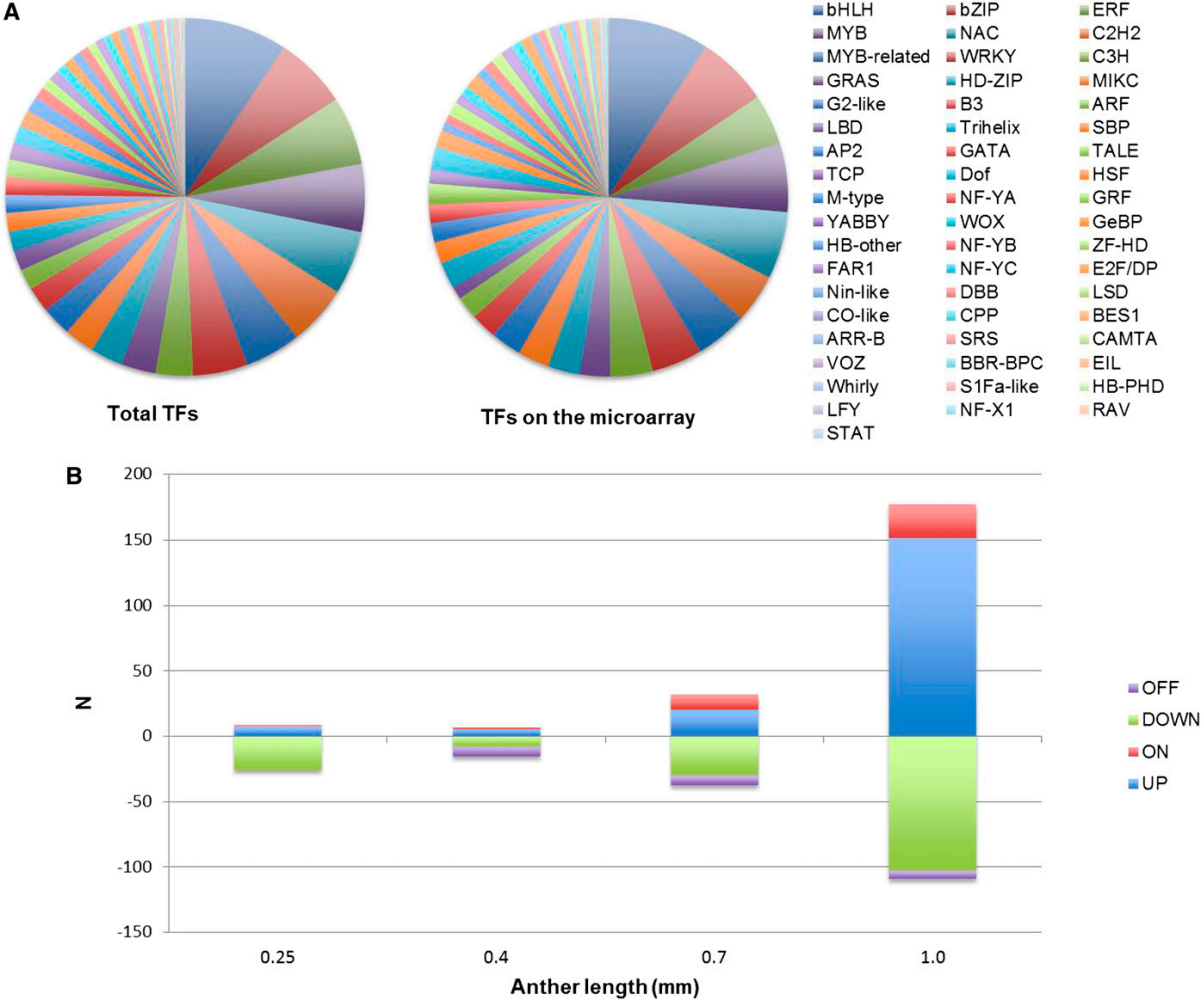
Evaluation of transcription factor microarray data. (A) 3307 predicted TFs classified into 56 families (left pie chart); 1208 of them were represented on the microarray (right pie chart) in approximately equivalent proportions. (B) Transcription factor abundance changes at each stage. ON and OFF indicate detectability at that stage. UP and DOWN classifications for a transcript were determined relative to the previous stage using a two-fold minimum fold change.

To study whether master regulators control the transcriptional changes during anther development, we examined the expression profiles of known transcription factors (TFs). There are 2298 TF loci in maize and, as a result of alternative splicing, these encode 3307 predicted TFs classified into 56 families (Jiang *et al.* 2012). Among these, 1208 TF loci (52.6%) are represented by 1901 probes on the microarray. The distribution is a fair representation of each family (Figure 6A); 813 TFs represented by 1041 probes are constitutively expressed from 0.15 to 1.0 mm. During the initial stages of anther development, we found few changes in specific TF abundance (Figure 6B, Table S6). In contrast, 178 TF transcripts were increased (including 151 transcripts that are at least two-fold more abundant and 26 transcripts that are newly detectable) at 1.0 mm compared to 0.7 mm; 103 transcripts were decreased by more than two-fold at 1.0 mm (Figure 6B, Table S6) as well. These findings are consistent with the observation that there are both more transcript types and higher expression levels when anthers are approaching meiosis at 1.0 mm (Figure 4A), suggesting that large-scale transcriptome reprogramming is important for anther differentiation at this stage.

Analysis of *mac1* mutants (Wang *et al.* 2012b) demonstrated that successful PMC entry into meiosis does not require normal anther somatic tissues. A properly differentiated PMC is required for successful entry into meiosis, however, because differentiation failure in *ameiotic1* (*am1*) mutants (Nan *et al.* 2011) results in cytologically normal PMC conducting mitosis instead of meiosis. Based on differential expression in *ameiotic1*, 297 genes were associated with meiotic preparation. Surprisingly, 104 of these are expressed by the 0.35 mm stage in laser-microdissected AR (Kelliher and Walbot 2014). To focus on factors with more strictly defined roles in meiosis, we classified genes either as having established roles in maize or as maize genes that are homologs of proven meiotic factors in other plants (Table 1). Five meiotic genes have been characterized in maize: *Ameiotic1*, a plant-specific gene controlling the switch from the mitotic to meiotic cycle (Pawlowski *et al.* 2009); *Absence of first division1*, a meiosis-specific gene that controls meiotic chromosome formation and sister chromatid cohesion (Golubovskaya *et al.* 2006); *Poor homologous synapsis1*, a novel gene required for loading recombination machinery onto chromosomes and for homologous pairing (Pawlowski *et al.* 2004); and two paralogs of *Rad51* (Lin *et al.* 2006) — *Rad51a1* and *Rad51a2* — that play roles in homologous recombination and the repair of double-strand breaks (Li *et al.* 2007). Except for *Ameiotic1*, which is not detectable in 0.15 mm and 0.4 mm anthers, these genes are expressed constitutively at all five stages (Table 1).

**Table 1:** Known maize meiotic genes with a specific function and maize homologs of well-established meiotic genes in other flowering plants

**Known Gene**									
Protein Name	Gene ID	Function Timing	Function	Reference	0.15 mm	0.25 mm	0.4 mm	0.7 mm	1.0 mm
Ameiotic 1	GRMZM5G883855	Prophase I	Switch from mitotic to meiotic cell cycle	Pawlowski *et al.* 2009	Off	On	Off	On	On
AFD1/REC8	GRMZM2G059037	Leptene	Establishment of meiotic chromotin structure	Gloubovskaya *et al.* 2006	On	On	On	On	On
PHS1	GRMZM2G100103	Leptene-zygotene	Homology search	Pawlowski *et al.* 2004	On	On	On	On	On
Rad51a1	GRMZM2G121543	Leptene-zygotene	Recombination, required for homologous pairing	Li *et al.* 2007	On	On	On	On	On
Rad51a2	GRMZM2G084762	Leptene-zygotene	Recombination, required for homologous pairing	Li *et al.* 2007	On	On	On	On	On
**Homologs of Meiotic Factors**									
SPO11-1	GRMZM2G129913	Leptene-zygotene	Meiotic DSB formation	Yu *et al.* 2010	On	On	On	On	On
SPO11-2	GRMZM5G890820	Leptene-zygotene	Meiotic DSB formation	Yu *et al.* 2010	N/A	N/A	N/A	N/A	N/A
SPO11-3	GRMZM2G052581	Leptene-zygotene	Meiotic DSB formation	Yu *et al.* 2010	On	On	On	On	On
Rad51b	AC219006.2_FG007	Zygotene	Recombination	Lin *et al.* 2006	On	On	On	On	On
Rad51c	GRMZM2G123089	Zygotene	Recombination	Lin *et al.* 2006	On	On	On	On	On
Rad51d	GRMZM2G055464	Zygotene	Recombination	Lin *et al.* 2006	On	On	On	On	On
XRCC3	GRMZM2G157817	Zygotene	Recombination	Lin *et al.* 2006	On	On	On	On	On
ZEP1/ZYP1	GRMZM2G143590	Zygotene	Component of the synaptonemal complex, regulates the numbers of crossovers	Wang *et al.* 2010b	On	On	On	On	On

Expression patterns during early anther development are indicated at five developmental stages. Gene expression marked as “N/A” for genes not represented on the microarray.

Based on well-studied genes in rice and *A. thaliana*, additional meiotic factors were analyzed, including the following: *SPO11*, which generates double-stranded breaks along meiotic chromosomes (Yu *et al.* 2010); *DMC1*, which repairs double-stranded breaks using the sister chromatid as template initiating a crossover event (Couteau *et al.*1999); and *ZYP1*, a central component of the synaptonemal complex (Wang *et al.* 2010b). These genes are also constitutively expressed throughout the duration of early anther development (Table 1). Because meiosis-associated genes are expressed in 0.15 mm anther cells or the very first AR, if present, it is clear that very immature anthers activate expression, albeit at a low level, of some meiosis-specific genes. To test this hypothesis further, we utilized the list of 297 genes mis-regulated in *am1* mutants (Nan *et al.* 2011) to generate a list of 222 genes whose functions have been well-annotated; 77.5% of them (172/222) were expressed constitutively while only five gene transcripts were undetectable until 1.0 mm when meiotic preparation has been considered to start as AR differentiate into PMC (Table S7). Our data strongly suggest that young anthers are preparing for meiosis as early as anther organ identity has been set.

### Transcriptome profiling of outer somatic, inner somatic, and AR cell types from 0.7 mm anthers

**Figure 7:**
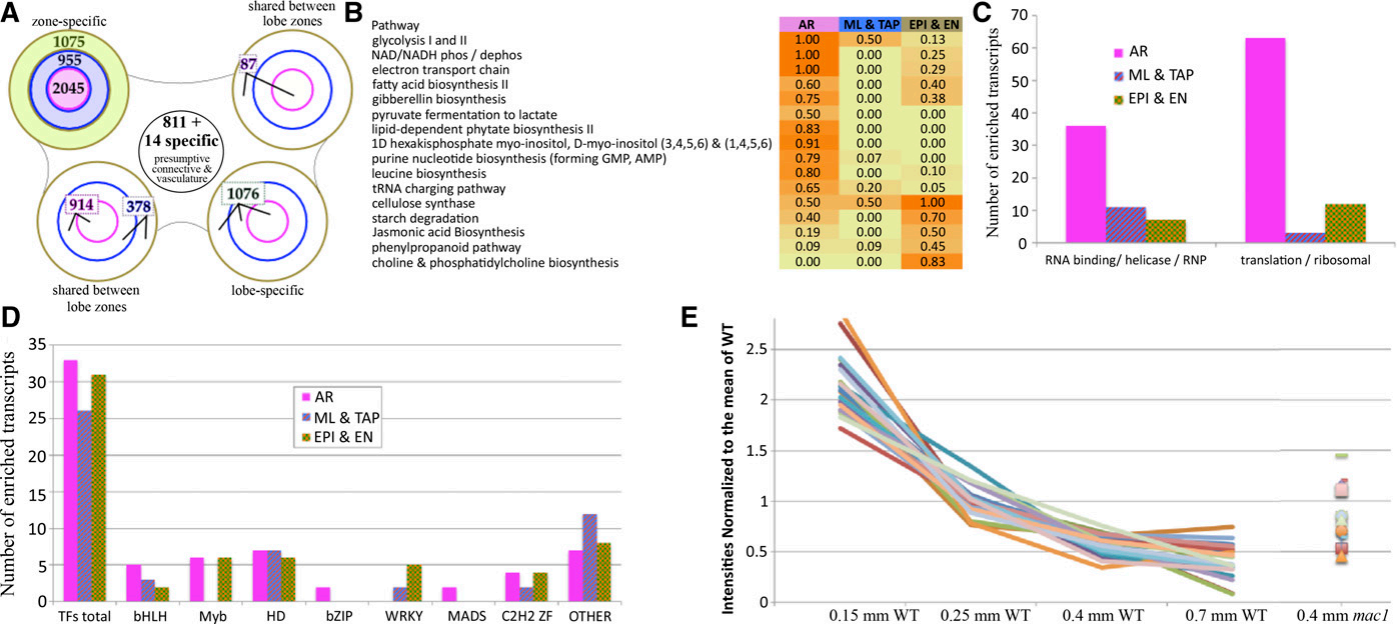
Distribution of enriched transcripts and pathway analysis in pre-meiotic anther cell types. (A) RNA samples from three laser-microdissected cell types were compared to equivalently staged whole anthers, and the distribution of significantly enriched transcripts is shown. The combined EPI/EN collection (green) had 1075 enriched transcripts (top left lobe). The combined ML/TAP collection (blue) had 955 enriched transcripts, and the germinal AR cell collection (red) had 2045 enriched transcripts. The other lobes in the diagram show the number of enriched transcripts shared between two (top right, bottom left) or all three cell type collections (bottom right). In the middle of the diagram, 811 enriched plus 14 specific transcripts were assigned to the connective and vasculature tissues by virtue of being DOWN or OFF in all three cell collections compared to whole anthers. (B) The data were analyzed using the MaizeCyc database to identify molecular pathways that were enriched in at least one of the cell types; the criterion was that >45% of the composite enzymes had to be enriched in a particular cell type. Sixteen pathways fit this criterion, including 11 enriched only in AR cells, five enriched only in EPI/EN cells, and one, cellulose synthesis (required for construction of the cell wall), enriched in both AR and EPI/EN collections. Zero pathways were identified in the ML/TAP sample, likely because at this stage these cell types are just starting to differentiate after birth from a periclinal division of the bipotent SPL. (C) The number of transcripts annotated as encoding RNA binding proteins, RNA helicases, ribonucleoproteins (RNPs), ribosomal proteins, or translation initiation and elongation factors was much higher in the germinal cells than either of the two somatic cell collections. (D) Distribution of specific classes of transcription factors in the anther lobe cell types is indicative of massive transcriptional reprogramming during differentiation. Overall, the three cell type collections had similar numbers of enriched TFs and similar numbers of homeodomain (HD), C2H2, and bHLH TFs. Myb TFs were enriched in AR and EPI/EN cells but not in the ML/TAP, whereas only AR cells were enriched in bZIPs and MADS box TFs. Seven WRKY transcription factors were enriched in somatic cell types but none in the AR. (E) Expression intensities of pluripotency markers during the early anther stages normalized to the mean. These 21 markers are gradually shut-off during early anther development as greater numbers of cells differentiate away from pluripotency. All but three of these markers show elevation at the 0.4 mm stage in *mac1* as compared to wild-type, supporting morphological interpretations of that mutant as having an extended period of pluripotency concomitant with failure to properly differentiate somatic (EN/SPL) cell types.

By the 0.7 mm stage, all cell fate specification events are completed, and the resulting cells are dividing anticlinally to fuel anther growth in length and girth. By this stage the EPI, EN, and AR have distinctive cellular properties and dimensions. The ML and TAP cell types will soon acquire distinctive cellular dimensions and staining properties (Kelliher and Walbot 2011), visual indicators of cellular differentiation. Three samples were collected via laser capture microdissection (LCM) to compare directly to whole anthers of the same stage: the outer somatic sample contains EPI plus EN cells (EPI/EN), the inner somatic sample contains two layers (ML/TAP), and the AR cells were dissected as the third sample (Figure 7A). Compared directly to equivalently staged whole anthers, there were 19,743 common transcript types, 6728 present in whole anthers but absent from the LCM samples, but only a few were found in LCM samples but not whole anthers. The majority of those absent from the three LCM samples had low expression in whole anthers; only 235 (3.5%) are above the first quartile and 14 (0.2%) are above the median, including two TFs. This suggests that many of these are actually present in lobes, but below detection using our methods. Adding the 14 transcripts above the median to the 811 downregulated in all three LCM samples, we can assign 825 transcripts to non-lobe tissues (vasculature and connective cells) (Figure 7A); these are the first presumptive markers assigned to these non-lobe cell tissues. Each LCM sample also exhibited a large set of significantly enriched transcripts (1075 in EPI/EN, 955 in ML/TAP, and 2045 in AR) (Figure 7A). Particularly for the differentiated cell types (EPI/EN and AR), the enriched transcript types point to processes likely to be characteristic of specific cell types.

**Figure 8:**
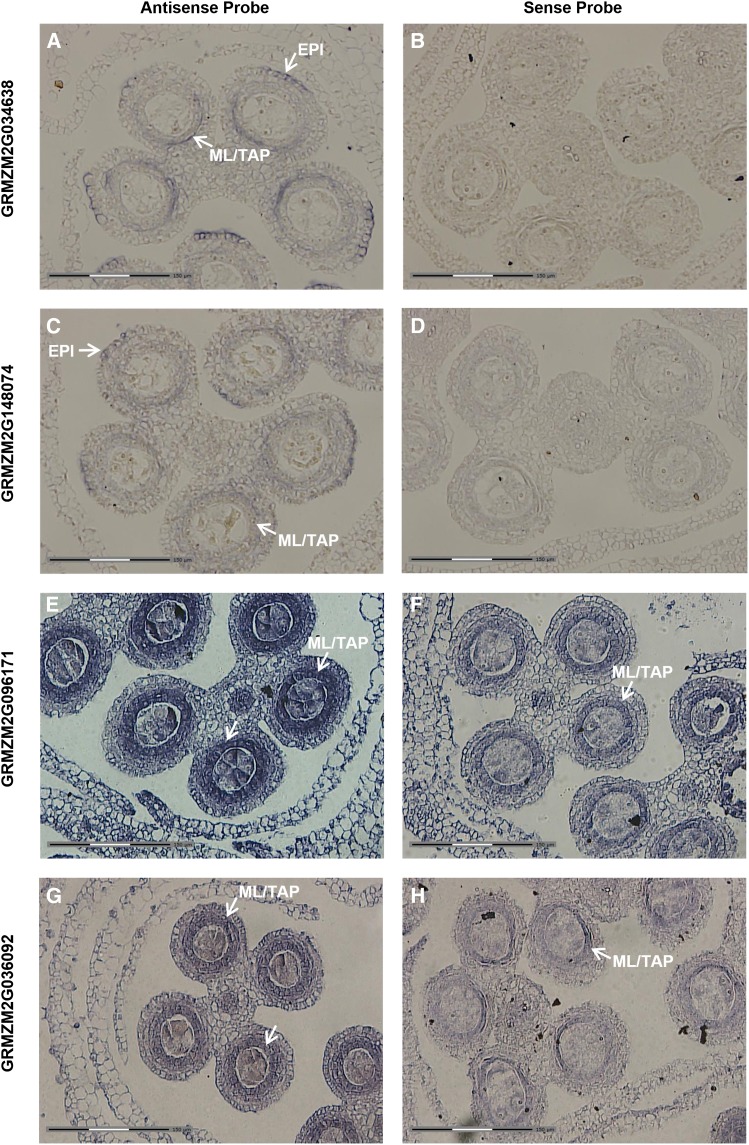
*In situ* candidates for cell-enriched markers. Genes were chosen for *in situ* analysis to verify patterns selected as somatic-specific in the 0.7 mm laser-microdissected data set. Antisense probes (A, C, E, G) for each gene and the corresponding sense probes (B, D, F, H) are shown. As predicted, all genes showed significant enrichment in the somatic cells in lobes of anthers ≥0.7 mm. (A, B) GRMZM2G034638, a predicted EDG-6 sphingosine 1-phosphate receptor. (C, D) GRMZM2G148074, an HD-ZIP protein with predicted DNA binding and transcription regulator activity. (E, F) GRMZM2G096171, a CheY-like protein with predicted signal transducer and transcription regulator activity. (G, H) GRMZM2G036092, a predicted DNA binding protein with transcription regulator activity. White arrows point to cell types where the signal is detected. The white section of the scale bars in the lower left of each panel corresponds to 150 µm.

To determine if transcripts exhibiting zone-specific hybridization could be useful markers for zones or cell types, RNA *in situ* hybridization was performed on four transcripts (Figure 8) that each showed increased expression (fold-change >1.5-fold; p-value < 0.05) in the somatic cell types (ML/TAP, EPI/EN) and whole anthers compared to the AR. By *in situ* localization with ∼0.7 mm anthers, both GRMZM2G034638 and GRMZM2G148074 show expression in the ML/TAP and at the peripheral edge of the lobe arch, specifically in a restricted zone of the EPI (Figures 8, A and C). Interestingly, the male sterile mutant *outer cell layer 4* (*ocl4*) produces an extra subepidermal layer in peripheral anther lobes, and *Ocl4* is specifically expressed in normal EPI in the zone adjacent to the defective double endothecial layer observed in mutants (Vernoud *et al.* 2009). The authors proposed that OCL4 protein could have a direct effect on anther cell layer differentiation by signaling between the epidermis and endothecium. Because GRMZM2G148074 encodes an HD-ZIP protein likely to be a TF and GRMZM2G034638 encodes an EDG-6 sphingosine 1-phosphate receptor and is likely to be involved in signaling, the pattern of expression in both the peripheral EPI arch and the ML/TAP hints at an unexpected relationship in developmental control between the external and internal somatic layers. Confirmation of two additional markers for the EPI peripheral arch is very intriguing, because it suggests that these cells may have a particularly important role in coordinating anther development.

Two other transcripts, GRMZM2G096171 and GRMZM2G036092, encoding a CheY-like protein and a DNA-binding protein, respectively, show specific expression in the ML/TAP in ∼0.7 mm anthers and are thus presumptive markers for those anther cell types (Figure 8, E and G). Although there is similar expression in these cell types with the sense control probes (Figure 8, F and H), this is not unusual with maize anther genes (Kelliher and Walbot 2014). Globally approximately 20% of maize genes tested in a microarray experiment express an antisense transcript (Ma *et al.* 2006). The prevalence of antisense transcription during cell differentiation may indicate a mechanistic role for antisense transcripts to control anther development.

### Analysis of tissue-specific pathways

Mapping the transcriptome data from the LCM samples onto the MaizeCyc database highlighted the cellular processes and biosynthesis pathways enriched in each sample type (Figure 7B). There were five pathways highly enriched in the EPI/EN sample, including JA biosynthesis and phospholipid and cellulose biosynthesis. The identification of JA biosynthesis is intriguing, given its key role earlier as a signal in carpel abortion in tassel spikelets (Acosta *et al.* 2009). JA is a gas that could readily diffuse from the EPI to cause carpel elimination from each floret; therefore, we hypothesize that EPI is the site of JA synthesis in anthers. Application of 1 µM JA around developing tassels during cell fate specification (anthers 0.3–0.7 mm) can result in cessation of anther development (Walbot and Skibbe 2010), suggesting that JA may also regulate anther developmental processes as suggested by the pathway analysis of whole anther hormone-related genes. Membrane and cellulose production could support the massive proliferation of EPI and EN to build the axial orthogonal array (Kelliher and Walbot 2011), a cellular bilayer that provides structural support to the anther. There were no pathways enriched in the ML/TAP sample, likely because these cell types are just starting to differentiate (Figure 7B).

The AR is the most distinctive of the three tissue samples. Hypoxia is a critical physiological factor in triggering AR specification in anther primordia (Kelliher and Walbot 2012). The AR at 0.7 mm are enriched in transcripts for alternative energy pathways that perform non-mitochondrial energy generation and permit cellular survival in hypoxic conditions, as elaborated in a recent article focused on earlier stages of AR cell development (Kelliher and Walbot 2014). For example, there is enrichment for glycolysis I and II and NAD/NADH activity (Figure 7B). Hypoxic conditions are likely to persist in the center of the tightly packed anther lobes, given the high metabolic demand of actively dividing and growing somatic cells and the low oxygen availability in the air space around tassels (Kelliher and Walbot 2012). The AR are also enriched in pathways required for translation: genes encoding ribosomal proteins, translation factors, and tRNA charging enzymes are all enriched (Figure 7C). Predicted RNA-binding proteins are also highly represented in the AR, and are a common transcript type in both plant and animal reproductive cells (Extavour and Akam 2003; Schmidt *et al.* 2011) to manage translation and to sequester mRNAs for later use. Two additional pathways enriched in AR are related to myo-inositol metabolism and gibberellin biosynthesis; however, not much is known about these two pathways in anthers. Genes required for the latter are activated both in AR cells and in whole anthers at the 0.15 mm to 0.25 mm stage during AR specification.

To investigate whether the larger class of trough transcripts at 0.4 mm in normal anthers was quenched as a prerequisite for appropriate cell differentiation, we reexamined the restoration of gene expression for this set at 0.7 mm in the laser-microdissected cell type dataset. Although 2.3% of genes remained OFF at 0.7 mm and 62% showed no significant enrichment by cell type, 20.8% of the genes were scored as ON in a highly restricted pattern (169 in AR, 204 in ML/TAP, and 235 in EPI/EN), whereas 14.8% showed refined expression in two of three zones (143 in ML/TAP + EPI/EN, 188 in ML/TAP + AR, and 102 in AR + EPI/EN) (Table S8). These could be examples of spatial refinement of transcripts expressed in undifferentiated cells that later become zone or cell type specific. The existence of 102 genes common between AR + EPI/EN, nearly as many as the somatic-only shared set of ML/TAP + EPI/EN, supports the case that AR and EPI are the abaxial and adaxial epidermal sides of an ancestral “flat fertile frond” that rolled up, fused along the edge, and generated a circular organ, the modern anther.

Refinement of cell functions can require both increased and decreased gene expression. To analyze this hypothesis, we examined regional specialization in TF representation at 0.7 mm. Ninety TFs are significantly enriched in only one of the three sample types: 31 in EPI/EN, 26 in ML/TAP, and 33 in AR (Figure 7D). Of particular interest, only the AR cells were enriched in MADS box and bZIP TFs (two of each class). The EN/EPI and AR samples each had six Myb TFs but the ML/TAP sample had none. In contrast, WRKY family TFs were only enriched in the ML/TAP and EPI/EN cell types (Figure 7D). These data indicate that in addition to stage specificity of TF representation, there is also spatially restricted expression of TF as would be required for many aspects of cell differentiation.

### Transcriptome profiling of *mac1* and delays in fate acquisition

To further understand the 0.4 mm expression trough, we examined gene expression in 0.4 mm *mac1* mutant anthers compared to wild-type anthers of the same size. During early anther growth, the MAC1 protein is secreted by AR cells after germinal fate acquisition, stimulating a periclinal division in the single-layered encircling L2-d tissue to produce somatic EN and SPL daughters. In the *mac1* mutant, the L2-d cells are unable to produce EN and SPL daughters but continue to generate supernumerary AR cells, extending the period of L2-d pluripotency (Wang *et al.* 2012b). Of the 3871 transcripts scored OFF at the 0.4 mm stage after being ON in both 0.15 mm and 0.25 mm anthers, more than 84% (3252) are ON in 0.4 mm *mac1*, supporting the idea that these transcripts are normally shut-off concomitant with the completion of the first round of cell fate acquisition. Persistence of expression in the *mac1* mutant of transcripts that are normally downregulated to very low levels or are completely missing substantiates the idea that transient suppression of many transcript types is an important developmental feature of the 0.4 mm stage during which newly established cell types begin to differentiate and proliferate anticlinally to build a larger anther.

To define a set of transcripts strongly associated with pluripotency, *mac1* transcriptome data were filtered sequentially by three criteria. First, significant downregulation between 0.15 mm anther primordia and 0.25 mm partially differentiated anthers (log2 < −0.58; p-value < 0.05), then significantly downregulated again between 0.25 mm and 0.4 mm, and finally the requirement that transcripts remain low or OFF at later stages. Filtering for these parameters, we recovered 21 transcripts, many of which are already known to be highly abundant in tassel primordia (Bolduc *et al.* 2012), including a bHLH and two Yabby-like TFs (Figure 7E, Table S9). Eighteen of these have a higher intensity value in 0.4 mm *mac1* than in fertile anthers, and 10 can be classified as significantly enriched in this mutant, which is defective in specifying somatic cell fates. Thus, the classification of these 21 transcripts as anther pluripotency markers is supported by both wild-type and male-sterile mutant expression data.

To further investigate the molecular processes involved in the transition from pluripotency to bipotency, we examined the transcriptome changes in the *mac1* mutant more thoroughly at both the 0.2 mm and 0.4 mm stages in direct comparison to fertile anthers of the equivalent size classes. At 0.2 mm, the difference between mutant and fertile is not yet apparent microscopically, and we would expect changes only in the set of genes whose transcription is directly affected by the lack of MAC1 protein. At 0.2 mm there are 301 genes differentially regulated (193 up, 108 down) in *mac1* compared to wild-type, along with 16 transcripts specific to wild-type (Figure S10, Figure S11). By 0.4 mm, *mac1* anthers contain three-times to five-times more AR and lack both the EN and SPL layers characteristic of fertile anthers. At this stage there are 1082 differentially regulated transcripts (328 up, 754 down) in *mac1* compared to wild-type, along with 258 present in wild-type but missing from *mac1* (Figure S12, Figure S13). To identify genes regulated directly by the MAC1 signaling pathway, we looked at the overlap of the two stages of transcriptome comparisons to wild-type (0.4 mm and 0.2 mm) and identified 23 downregulated and 24 upregulated transcripts (Figure S14, Figure S15). Of the 754 genes downregulated in *mac1* only at the 0.4 mm anther length stage, 287 are upregulated at 0.7 mm in the EPI/EN, suggesting these are already EPI/EN transcripts at 0.4 mm. These are likely candidate differentiation markers for the EN (Table S12). Another 137 are also upregulated in the ML/TAP, suggesting these may be SPL transcripts at 0.4 mm (Table S12).

## Discussion

Prior studies reported W23 maize anther transcriptomes during pre-meiotic preparation (1.0 mm), several stages of meiosis (1.5 mm, 2.0 mm, 2.5 mm), and key stages of post-meiotic (3.0 mm, 4.0 mm, mature pollen) ontogeny (Ma *et al.* 2008). The current study analyzed the transcriptomes of much younger anthers, including the 0.15 mm primordium prior to L2-d cell fate setting, at 0.25 mm when the archesporial cells are present and are regulating periclinal division in the L2-d to establish the endothecial and secondary parietal founding cells, at 0.4 mm when all cell types are proliferating to increase anther size, at 0.7 mm after the final cell fate specification event accomplished by the SPL cells to establish the middle layer and tapetum, and at 1.0 mm when the phase of AR mitosis is finished and the somatic cells have all acquired distinctive shapes, volumes, and staining features indicative of cell differentiation. The transcriptome analyses for whole maize anthers are now available for all the landmark developmental stages, and several common themes are clear.

### Transcriptome complexity

First, the anther transcriptome is very complex and dynamic. Such complexity was noted in the earliest studies of dicot anther and pollen RNA composition (Goldberg *et al.* 1993), and this has remained a consistent observation for all species evaluated. Why would anthers express so many genes given the relative simplicity of tissues and cell types? Gametophytic transcriptome complexity could be a selected feature to eliminate deleterious mutations — unlike animal gametes, the haploid cells of plants are transcriptionally active and synthesize cellular structural components, factors required for mitosis, metabolic enzymes, and proteins required for their specialized functions as gametes or helper cells. A haplosufficiency test in the microgametophytes could purge the genome of deleterious mutations in genes that are also important in diploid sporophytes. Prior to meiosis, all anther cells are diploid, and we speculate that the diverse gene expression program could be a pre-meiotic diploid robustness test—a process that allows for selection for alleles that are sufficient to program normal development in plants heterozygous for a non-functional allele.

The relatively high number of genes expressed may also reflect a more general characteristic of plant stem cells, which are abundant in maize anthers as pluripotent L2-d cells up to the 0.25 mm stage and then as bipotent SPL up to the 0.7 mm stage. Do plant stem cells exhibit basal transcription of thousands of untranslated mRNAs as a consequence of a largely non-repressive global chromatin status? Low levels of transcript production in stem cells would later be refined during cell fate acquisition for thousands of genes that are either selectively upregulated to provide mRNA for protein production or downregulated to eliminate transcription. To test this idea, it would be interesting to determine whether the thousands of low-abundance transcripts in stem cells are translated into proteins and, if not, whether the mRNAs are stored for later use or degraded at a later stage. It is not uncommon to identify plant genes with genetically defined developmental roles that are also expressed outside their predicted cell-specific and temporal patterns. For example, the AR-somatic patterning gene *Mac1* is expressed in roots, which lack germinal cells and exhibit no mutant phenotype (Wang *et al.* 2012b). This could be a case of unregulated expression, or *Mac1* transcription may hint at a more general role of MAC1 in forming the nested rings of cells by periclinal division, a function that may be masked in *mac1* roots by a functionally redundant factor that is not expressed in anthers. Another example of ectopic transcription is the meiotic gene *DNA meiotic recombinase 1* (*Dmc1*). The protein is responsible for crossover formation in meiotic cells and, despite having no role in vegetative tissues, the transcript is found in both silks and embryos (http://maizegdb.org/cgi-bin/displaygenemodelrecord.cgi?id=GRMZM2G109618). Deep proteome data would be necessary to address this issue of the meaning and timing behind transcription of such an enormously complex and dynamic anther transcript set.

A third contributor to complexity may be the multiple roles played by anther cell types over the 30 d from inception to pollen shed. Organs such as leaves, stems, and roots are built with specific, generally terminally differentiated cell types to perform a more fixed range of functions. In contrast, anther cells continue to exhibit new properties as the organ matures. The germinal cells are an obvious example: initially they are mitotic AR, then PMC, then haploid meiotic products, then developing gametophytes, and, finally, they are mature pollen. Accompanying these major changes in germinal cell functions, somatic cells are also changing roles. For example, one of the first differentiated roles of tapetal cells is to secrete callase to remodel the callose coat of PMC cells (Wang *et al.* 2010a) prior to meiosis; the tapetum is also the likely source of nutrients for the AR, PMC, and developing pollen. After meiosis, tapetal cells differentiate to synthesize and secrete a wide range of molecules that are deposited as exine components on developing pollen grains. In maize, the developing pollen stick to modified tapetal cell walls; the grains are arrayed helically within the lobe surrounding a large locular airspace (Kelliher and Walbot 2011). Eventually the tapetal cells die, and the remnant cell walls become part of the structural stiffening of the anther. Similarly, other somatic cell types undergo sequential changes in function to facilitate the ultimate role of pollen dispersal. Although pollen dispersal mechanisms differ among flowering plants, anther dehiscence inevitably involves changes in the epidermis and endothecium to allow pollen out of the encasing somatic tissues.

A fourth possible explanation for transcriptome complexity is that germinal and somatic cells may synthesize then store mRNAs required at later stages of development. This is a long-recognized, major mechanism in animal pre-meiotic cells (for example, in *Drosophila melanogaster*) (Spradling 1993; Fuller 1993). The abundance of transcripts corresponding to RNA binding GO annotation, particularly in AR cells, indicates that the role of stored mRNA should be explored in anther cells as well. The stored mRNAs of animal eggs and sperm are required to program early embryo development. In the case of plants, such stored mRNAs may be required during the cascade of differentiation events in both the germinal and somatic anther cells. In some animal embryos such as *Caenorhabditis elegans*, there are periods of massive transcript loss (Levin *et al.* 2012) ascribed to the degradation of maternally deposited mRNA to facilitate a differentiation step in moving from one larval stage to the next. The cohorts of anther genes exhibiting a trough of expression could represent similar cases in which elimination and then re-expression of a transcript type are required for normal development.

One approach to documenting stored mRNA is to demonstrate a temporal separation between mRNA maturation and translation into protein products. Our initial anther proteomes are insufficient to answer this question on a broad scale because we sampled only the most abundant proteins in whole anthers. Much deeper proteome sampling or antibody reagents to individual protein types will be required to adequately address the possibility of stored mRNA during anther ontogeny. Of particular interest in this regard is the striking early synthesis of transcripts for proteins with documented roles in meiosis. Transcripts for a number of well-studied meiotic factors and a longer list of meiosis-associated transcripts appear 5 days before meiotic preparation is considered to start when AR differentiate as PMC. These transcripts are present during AR specification, albeit often at low levels, and persist through the period of AR mitotic proliferation. These observations are further evidence of the distinctiveness of AR once specified (Kelliher and Walbot 2012).

Reproduction is the purpose in constructing an anther, and the AR cells are the most distinctive cell type in the organ. They undergo successive differentiation events, from AR to PMC to haploid meiotic products, before dividing to produce binucleate microspores and, finally, mature, trinucleate pollen. With each stage, these reproductive cells exhibit distinct morphology, cytology, gene expression patterns, and functions. Much has been learned about the post-meiotic transcriptome of developing microspores and mature pollen, but comparatively little is known about the pre-meiotic stage. In size they dwarf the inner and outer somatic cell types, growing to a maximum diameter of 50 µm at the PMC stage (Kelliher and Walbot 2011). From this study and a recent one focused on germinal and somatic transcriptomes just following AR differentiation (Kelliher and Walbot 2014), the following principles are established in maize. First, based on gene expression profiles, AR cells differentiate rapidly from their L2-d precursors and are distinct from the specified somatic cell types. Second, mirroring the distinctive roles of these reproductive cells, isolated AR, PMC, and pollen exhibit distinct transcriptomes. Third, despite the distinctive features of each stage and contrary to prevailing models, the AR from the earliest stage measured express meiosis-associated genes. These transcripts, constituting approximately one-third of the meiosis-associated list (Kelliher and Walbot 2014) including several strictly meiotic factors (this study), are expressed at low levels prior to PMC differentiation but are nonetheless enriched in AR cells within 1 to 2 days of their specification. This observation merits additional study to clarify whether these precociously expressed transcripts are translated into protein, and whether the proteins are involved in early differentiation for meiosis, before the AR differentiate into PMC when meiotic preparation has been assumed to occur. Fourth, AR cells exhibit mainly autonomous development, because in the absence of normal somatic neighbors, as in the *mac1* mutant (Wang *et al.* 2012b; Kelliher and Walbot 2012), AR mature to PMC and start meiosis.

As established here, transcriptome profiling indicates that the AR initially differentiate by expressing transcripts involved in energy and RNA metabolism. Just 1 to 2 days after their birth, AR are enriched in the following: alternative metabolism transcripts, which are responsible for the AR thriving in a hypoxic (<2% O_2_) environment; RNA binding transcripts, which may be involved in long-term RNA storage; and ribosomal protein transcripts, suggesting that AR boost translational capacity or build ribosomes with distinctive properties. Future studies could address carbon physiology of AR cells to define the energy budget and determine the roles of RNA binding and ribosomal protein gene expression patterns in supporting AR proliferation and development. One of several additional unanswered questions is whether the PMC are transcriptionally silent during meiosis, an assumption based mainly on analyses of animal meiotic cells. Post-meiotic animal gametes, particularly sperm, are typically transcriptionally silent, whereas post-meiotic haploid gametophytes including mature pollen competent for fertilization are transcriptionally active. Consequently, silencing during meiosis may be part of the developmental program establishing silencing in animal gametes, a process that does not occur in plants. The maize mutant *ameiotic1* is a reminder that achieving normal PMC cytology is not a guarantee that meiosis will occur, because in this mutant PMC conduct mitosis instead. Those studying meiosis *per se* have focused on chromosome-level events. This focus has ignored the differentiation of the cytoplasmic and non-chromosomal nuclear compartments of germinal cells. Undoubtedly, many aspects of meiotic preparation involve new features of these as well, as previewed in the AR transcriptome assessment in which energy generation, RNA binding proteins, and ribosomal proteins are so prominent.

### Distinctiveness of early anther stages

We posed the question of whether each of the five stages of pre-meiotic anther development profiled would be distinctive. It is clear that four of the stages exhibit numerous stage-specific transcripts, indicative of distinctive processes and regulation of gene expression. This stage distinctiveness increases over developmental time, peaking at 1.0 mm when all cell types are differentiated. At 0.7 mm, the LCM samples of outer somatic cells (differentiated EN plus EPI) and differentiated AR exhibit distinctive transcriptomes and express genes correspondent with their functions. In contrast, the just-specified inner somatic layers (ML and TAP sister cells from a recent SPL periclinal division) show few zone-specific transcripts. This result indicates that specification is a required first-step in differentiation but, as is commonly assumed, that acquisition of differentiated cell features requires time.

Surprisingly, the 0.4 mm stage when EPI, EN, SPL, and AR are present has only 11 stage-specific transcripts. There is also a large decrease in transcript diversity at 0.4 mm —many transcripts that were expressed at low levels at the preceding 0.25 mm stage are not detectable by microarray hybridization—yet many of these transcripts are detectable a few days later at the 0.7 mm stage. We found this observation very puzzling and investigated it through bioinformatics analyses and additional experiments. From these experiments it is clear that this transient trough in transcript diversity reflects an active process that is part of normal differentiation. Our working model invokes two explanations for the trough. First, genes associated with pluripotency should disappear at 0.4 mm because normally all lobe cells have acquired a specific fate (including the bipotent SPL) by this stage. Such pluripotency genes are represented in the set mis-expressed by *mac1* at 0.4 mm, a mutant in which the pluripotent L2-d persist for far longer than normal and continue to generate extra AR until ∼0.4 mm. Second, we invoke a cell biological feature. The stages flanking 0.4 mm are periods of periclinal division and, hence, cell patterning in new tissue layers. We propose that many transcripts contributing to these processes are transiently downregulated at 0.4 mm when all cell division is anticlinal and, hence, contributing to anther growth in length and girth. Re-expression of the periclinal division programs in the SPL from 0.55 mm to 0.7 mm results in ML and TAP specification (Kelliher and Walbot 2011). We consider this a presumptive case of reuse of a developmental module within the context of anther development, a question we sought to address in our study. Future work could focus on identifying the regulators of periclinal division to test whether a few transcription factors are master regulators controlling periclinal rather than anticlinal division. Because ectopic periclinal divisions in the somatic cells cause dramatic developmental errors in anthers and result in male sterility (Timofejeva *et al.* 2013), tight regulation of this process appears to be essential to allow meiosis to proceed even if the AR are apparently normal, as in *mac1* (Wang *et al.* 2012b). Finally, it is of interest to understand how the trough transcripts are modulated in abundance: is it primarily through transcriptional control over short-lived mRNAs or are there post-transcriptional mechanisms that ensure degradation of this large class of transcripts at 0.4 mm and smaller transcript cohorts at other stages? Whether transcriptional or post-transcriptional, what is the refined temporal pattern of both the loss and re-gain of these trough transcripts and are they co-regulated in a highly synchronous manner?

In summary, the transcriptome and proteome profiling data for anther primordia through the stage of completion of AR mitosis provide a rich description of gene expression events at five stages of maize anther development. These data suggest many more questions than they answer, and the candidate genes and processes can guide future research identified in this study. In particular, we predict that further analysis of the regulation of periclinal division, analysis of transcriptome and proteome changes in developmental mutants, and *in situ* localization of mRNA and protein products will illuminate a more detailed understanding of anther development.

## Supplementary Material

Supporting Information
