# Supplementary material for: Transcriptomes and Proteomes Define Gene Expression Progression in Pre-meiotic Maize Anthers
Source: G3 (Bethesda). 2014 Jun 1;4(6):993–1010. doi: 10.1534/g3.113.009738 (PMC4065268; doi:10.1534/g3.113.009738)
Supplement: Supporting Information [file supp_4_6_993__index.html]

Supporting Information 

# Transcriptomes and Proteomes Define Gene Expression Progression in Pre-meiotic Maize Anthers

## Supporting Information for Zhang *et al.*, 2014

**Files in this Data Supplement:**

- Supporting Information - Figures S1-S2 and Tables S1-S15 (PDF, 360 KB)
- Figure S1 - Decile comparisons of transcriptomes and proteomes at threes stages. (PDF, 213 KB)
- Figure S2 - Comparison between the early anther proteomes with previously-published meiotic-stage small protein proteomes. (PDF, 163 KB)
- Table S1 - Comparative anther staging in *Arabidopsis thaliana* and *Zea mays*. (PDF, 172 KB)
- Table S4 - Quantitative real-time PCR results for 19 genes sampled from the microarray results. (PDF, 142 KB)
- Table S5 - Average intensities of hormone-related pathways. (PDF, 148 KB)
- Table S6 - Microarray intensities for transcription factor transcripts that exhibit differential accumulation during anther development. (PDF, 154 KB)
- Table S7 - Meiotic gene expression at five stages of early anther development. (PDF, 162 KB)
- Table S2 - Differential expression of genes across three stages of pre-meiotic anther development. (.zip, 24 MB)
- Table S3 - Gene lists included in cluster graphs in Figure 3. (.zip, 483 KB)
- Table S8 - Candidate cell-type enriched transcripts as shown in Figure 7 Venn diagrams. (.zip, 248 KB)
- Table S9 - Pluripotency markers. (.zip, 26 KB)
- Table S10 - Transcripts enriched in or specific to wild type over *mac1* at 0.2 mm. (.zip, 42 KB)
- Table S11 - Transcripts enriched in or specific to *mac1* over wild type at 0.2 mm. (.zip, 52 KB)
- Table S12 - Transcripts enriched in or specific to wild type over *mac1* at 0.4 mm. (.zip, 121 KB)
- Table S13 - Transcripts enriched in or specific to *mac1* over wild type at 0.4 mm. (.zip, 343 KB)
- Table S14 - Transcripts enriched in or specific to *mac1* over wild type at both 0.2 and 0.4 mm stages. (.zip, 48 KB)
- Table S15 - Transcripts enriched in or specific to wild type over *mac1* at both 0.2 and 0.4 mm stages. (.zip, 34 KB)
