# Supplementary material for: Transcriptomes and Proteomes Define Gene Expression Progression in Pre-meiotic Maize Anthers
Source: G3 (Bethesda). 2014 Jun 1;4(6):993–1010. doi: 10.1534/g3.113.009738 (PMC4065268; doi:10.1534/g3.113.009738)
Supplement: Supporting Information [file supp_4.6.993_009738SI.pdf]

G3: Genes | Genomes | Genetics

Transcriptomes and Proteomes Define Gene Expression Progression in Pre-meiotic Maize Anthers

Han Zhang*, Rachel L. Egger*, Timothy Kelliher*[1], Darren Morrow, John Fernandes, Guo-Ling Nan and Virginia Walbot

Department of Biology
Stanford University, Stanford CA 94305-5020 USA

*These authors contributed equally to this work.
[1]Current address: Syngenta AG, 3054 E. Cornwallis Rd., Research Triangle Park, NC 27709

Corresponding author contact information
Rachel Egger
385 Serra Mall, Department of Biology
Stanford University
Stanford, CA 94305-5020
Phone: (650) 723 -2609 Cell Phone: (612) 236-5615 FAX: (650) 725 - 8221
E-mail: egger@stanford.edu

GEO records GSE43982 and GSE13118 contain all of the microarray data.

DOI: 10.1534/g3.113.009738

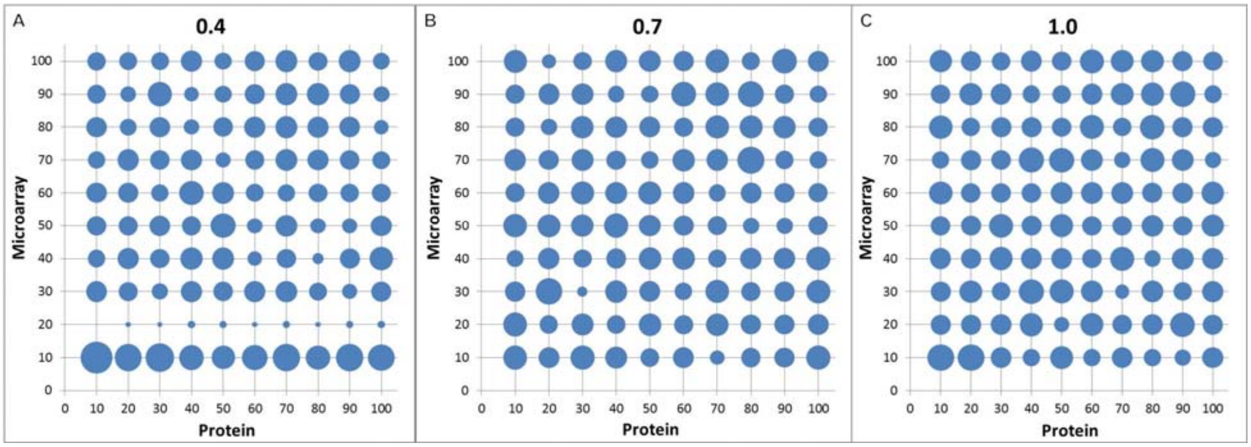

Figure S1 Decile comparisons of transcriptomes and proteomes at threes stages. Comparison of protein and transcript abundances at three developmental stages: 0.4, 0.7, and 1.0 mm. Data were divided into ten bins based on abundance. Circle diameters reflect the number of protein-transcript matches in each of the 100 bins.

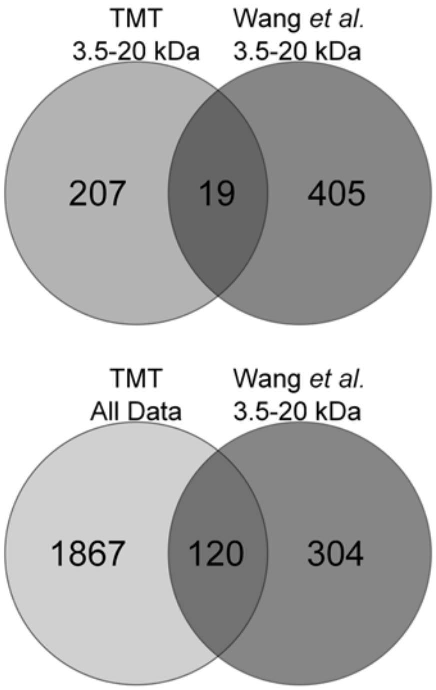

Figure S2 Comparison between the early anther proteomes with previously-published meiotic-stage small protein proteomes. Venn diagrams illustrating shared and unshared 3.5 – 20 kDa proteins identified in the present proteomes (*TMT*, including 0.4, 0.7, and 1.0 mm anther sizes) and published 1.5 plus 2.0 mm meiotic anther proteomes (Wang *et al*. 2010a). The upper Venn includes only proteins identified in the 3.5 – 20 kDa gel slices for both proteomes; the lower Venn examines proteins from any size class in the current data compared to the 3.5 – 20 kDa from Wang *et al.*

Table S1 Comparative anther staging in *Arabidopsis thaliana* and *Zea mays*.

Arabidopsis				Maize		
Anther stage <u>*/floral stage*</u>	Cell types <u>present</u>	<u>Hours</u>	Stamen length (µm) <u>*/anther*</u>	Cell types + <u>anther event</u>	<u>Hours</u>	Anther length <u>(µm)</u>
1 / *1-5*	L1 L2 L3	96	<10/ no data	L1 L2 primordium	96	0-120
2 / *6*	EPI/AR	30	<20/no data	EPI/L2-d/AR specification	56	70-220
3 / *7*	EPI/SPL/AR	24	<50/ no data	EPI/EN/SPL/AR	42	180-280
				Anticlinal mitotic proliferation	36	280-550
4 / *8*	EPI/EN/ML/TA/AR Specification done	24	55-60/*40*	EPI/EN/ML/TA/AR Specification done	24	550-700
				Anticlinal somatic cell proliferation	48	700-1200
5 / *9*	PMC mature	60	150/*120*	PMC mature	60	1000-1500
6 / *10*	Prophase 1 meiosis	12	300/*240*	Prophase 1 meiosis	48	1500-2000
Total hours		**246**			**410**	

Staging information for *A. thaliana* is taken from Smyth *et al*. (1990) and Sanders *et al*. (1999). Maize data are from Kelliher and Walbot (2011, 2012).

Tables S2-S3

Available for download at http://www.g3journal.org/lookup/suppl/doi:10.1534/g3.113.009738/-/DC1

Table S2 Differential expression of genes across three stages of pre-meiotic anther development. Transcriptome profiling data comparing global gene expression in whole anthers dissected at three developmental stages: 0.4 mm anther length (4 cell layers including SPL are present), 0.7 mm anther length (just after the birth of the tapetum and middle layer from the SPL), and 1.0 mm anther length (all cell fates are specified and cells are at least partially differentiated; start of the pre-meiotic transition of AR into PMC). Both the raw data (Columns A-BH) and the data after normalization between experiments (Columns BI-CQ) are included in the first tab of the worksheet.

Table S3 Gene lists included in cluster graphs in Figure 3. The microarray data were filtered to find transcripts with particular expression patterns over the course of anther development and the most biologically interesting of these patterns are illustrated in the cluster diagrams in Figure 3. The attached file contains the normalized intensity values, feature numbers and gene names of the transcripts included in those graphs in Figure 3, where each tab corresponds to a particular graph presented. The nomenclature exemplified by “OnOnOffOnOn”, denotes the detected presence or absence of those transcripts at particular stages in the following order: 0.15mm, 0.25mm, 0.40mm, 0.70mm and 1.0mm.

Table S4 Quantitative real-time PCR results for 19 genes sampled from the microarray results.

Feature Number	MaizeGDB Protein ID	MaizeGDB Protein Name	Microarray Expression Pattern			qRT-PCR Average C(t)			Pattern Verified
			0.4 mm	0.7 mm	1.0 mm	0.4 mm	0.7 mm	1.0 mm	
6513	N/A	N/A	OFF	ON	ON	26.7	26.6	26.6	No
10373	GRMZM2G113967	NAF	OFF	ON	ON	23.0	22.5	23.4	No
33683	GRMZM2G180916	Protein kinase-like	OFF	ON	ON	31.2	30.8	31.5	No
37851	GRMZM2G166665	lipopolysaccharide-modifying protein	OFF	ON	ON	28.4	28.5	26.9	No
44169	GRMZM2G131715	N/A	OFF	ON	ON	32.7	*32.8	32.8	No
18879	N/A	N/A	OFF	ON	ON	*28.1	25.1	23.8	Yes
29	GRMZM2G175761	Zinc finger, LIM-type	OFF	ON	ON	21.6	22.3	22.5	No
24021	GRMZM2G170457	hypothetical protein LOC100384355	ON	ON	ON	21.7	21.2	21.3	Yes
30914	GRMZM5G856777	Photosystem II CP43 chlorophyll apoprotein Precurs	ON	ON	ON	19.8	18.7	18.6	Yes
38273	GRMZM2G436092	N/A	ON	ON	ON	21.5	21.3	21.6	Yes
13365	GRMZM2G170120	Serine/threonine protein kinase, active site	ON	ON	ON	23.9	23.4	23.6	Yes
36944	GRMZM2G095892	LOC100281279	ON	ON	ON	23.2	23.6	24.1	Yes
5400	GRMZM2G166524	Leucine-rich repeat	ON	ON	ON	22.1	21.3	21.1	Yes
43905	GRMZM2G034882	Tropomyosin	ON	ON	ON	26.2	26.0	26.2	Yes
22801	GRMZM2G084779	K+ potassium transporter	OFF	OFF	OFF	28.8	28.6	28.6	No
34528	GRMZM2G130389	Serine/threonine protein kinase-related	OFF	OFF	OFF	**32.3	32.5	31.8	Yes
30365	GRMZM5G868296	Putative uncharacterized protein	OFF	OFF	OFF	30.6	31.1	31.1	Yes
39100	GRMZM2G111954	Aldo/keto reductase	OFF	OFF	ON	**30.1	*30.1	29.8	No
36385	GRMZM2G085872	CemA	OFF	OFF	ON	28.2	28.1	*28.3	No
33573	GRMZM2G134747	LOC100284448 (Cyanase)	ON	ON	ON	22.1	22.0	22.2	Yes

Feature Number	MaizeGDB Protein ID	Forward Primer Sequence	Reverse Primer Sequence
6513	N/A	GGCCATGAAACACCAGAAGG	AGGAAAGGTCACATGCACGA
10373	GRMZM2G113967	GACAGGCTATGATGGGGCAA	GCTGGGGTCGAGGATCTTG
33683	GRMZM2G180916	AATGTCTCGTGCTTGGCAGA	AGGCCAGCTTGGATTTGGAT
37851	GRMZM2G166665	TCCCCCTCCCTCTCTCTCTA	GCAGGACGGCAGGATACATA
44169	GRMZM2G131715	TGGCCGATCGAGCCTTTTT	ACGGTGGCAAGAATACAGCA
18879	N/A	TCTACACCTCCACGCTCGAC	GTCGCATACAACCACGGTCT
29	GRMZM2G175761	GAGAAGGGCAACTTCAGCC	TCTGAACGCACATGATCTCC
24021	GRMZM2G170457	CCCCTACTTCGATCTGGACA	TCTGAATGGAACCTGCTGTG
30914	GRMZM5G856777	TTCGCCAAATTACCCGAAG	ATTCAATTTGGCAGGCAGAA

38273	GRMZM2G436092	CGCAGTACGAGATTGGATGA	ACAGCACCAAATCCACACC
13365	GRMZM2G170120	AACCCGTCATCATTGTTGG	TCTGGTCTGTCTTGCAGGC
36944	GRMZM2G095892	CCAGTGTCTCAACTCAAGCG	CGGAGATCACGTAGAGGGG
5400	GRMZM2G166524	GGTTCTTGGGTTGGTGTCC	CCAGATCCATGTTCCCTCC
43905	GRMZM2G034882	ACCGAGGTCCTGAAGATGC	AATGCATCCAAACGAGGC
22801	GRMZM2G084779	CATAACAGACTGCTCCGGG	GAACAGAACCCTTCCATTTCC
34528	GRMZM2G130389	ACATGCATTGAAATTGGGC	AGCAAATCTCCATGGGTCC
30365	GRMZM5G868296	ACCTACGATTCCCTCACCG	CCCCAAATTTTCTTCCACG
39100	GRMZM2G111954	TCTGAACTGGCTGACCTGC	CTGCTACGACATGGGTTGG
36385	GRMZM2G085872	AACGAGGAGTGAATTGCTGC	TTTGAAGCTGATGAAGCGG
33573	GRMZM2G134747	GCTGGTGAGGAGGAGAAACA	CAGCAATCATGCCAGGTAGA

Candidate genes were chosen based on a pattern of expression using OFF or ON at 0.4, 0.7, and 1.0 mm whole anther stages as detailed under "Transcriptome Analysis – Data Analysis" in Materials and Methods. Patterns chosen to verify were: OffOnOn, OnOnOn, OffOffOff, and OffOffOn, relative to 0.4, 0.7, and 1.0 mm. Average $C_{(T)}$ values are shown using cyanase as an internal reference (Ma et al. 2007). Three biological samples and three technical replicates for each sample were used, except where indicated. Below, primer sequences for the transcripts examined in the qRT-PCR study. *One biological replicate excluded; **Two biological replicates excluded.

Table S5 Average intensities of hormone-related pathways.

Pathways	0.15 mm	0.25 mm	0.4 mm	0.7 mm	1.0 mm
**jasmonic acid biosynthesis**					
GRMZM2G002959	554.4	633.2	657.8	778.4	1323.3
GRMZM2G009479	244.1	303.1	300.6	333.5	298.2
GRMZM2G014136	152.5	142.7	140.7	104.9	175.5
GRMZM2G015419	108.5	91.5	83.3	114.4	113.1
GRMZM2G033491	290.2	244.3	218.9	397.5	352.7
GRMZM2G052389	267.5	183.6	137.7	172.4	177.3
GRMZM2G072653[a]	608.7	548.6	607.2	496.7	2133.3
GRMZM2G077316[a,b]	1044.6	1204.2	1328.3	1646.8	3669.4
GRMZM2G082087	142	145.7	149.1	161.7	270.1
GRMZM2G087192[a]	122.2	281.7	214.6	187.1	457.6
GRMZM2G102760	89.8	87.1	78.3	86.9	76.2
GRMZM2G104843	94.4	104.9	99.7	90.6	84.3
GRMZM2G106250	87	84.6	87.2	97.9	129.4
GRMZM2G109056[a]	4517	4803.9	4290.5	4761	2369.7
GRMZM2G109130[a]	1303.8	764.7	386.6	379.6	1768.2
GRMZM2G110201[a,b]	1409.9	1317.7	1390.3	1891.3	845.2
GRMZM2G117357	312.4	248	204.1	222.2	203.2
GRMZM2G132903[a,b]	508	531.1	549.5	423.2	1741.4
GRMZM2G146885	879.3	945.2	1017.6	646	998.2
GRMZM2G148281	1458.8	1271.5	1320	1378.5	1407.8
GRMZM2G151087[a]	3503.3	3650	3714.4	2928.4	8280.9
GRMZM2G156712[a]	143.4	161.7	165.6	111.4	230.1
GRMZM2G156861[a]	1133.5	432.6	199	112.5	3820.4
GRMZM2G168404	171.1	153.1	154.5	143.5	85.6
GRMZM2G398500	333.2	312.8	393.7	237.2	273.7
GRMZM2G415793	3569.9	3047.6	3782.3	7002.8	5972
GRMZM2G459755	84.9	92.7	98.2	90.9	72.3
GRMZM5G862219[a]	2417.9	2288.7	2159.6	2941.8	497.3
GRMZM5G864319[b]	1252.7	1061.9	1092	1407.4	546
**IAA biosynthesis I**					
GRMZM2G022192	123.9	115.2	113.2	128.2	93.7
GRMZM2G066345	122.6	148.1	153	87.7	139.8

GRMZM2G111225	175.2	151	139.7	197.4	111.9
GRMZM2G111309[a]	181.9	174.4	170.6	109	224.3
GRMZM2G117614[a]	348.1	304.2	320.4	290.3	690.4
GRMZM2G124175	93.4	106.7	108.1	115.1	95.6
GRMZM2G141473[a]	72.9	77	102.7	765.2	179.9
GRMZM2G141535[a]	297.9	598.2	750.8	1532.8	1941.3
GRMZM2G159542	230.9	223.2	205.9	267.1	137
GRMZM2G169087	95.1	94.4	81.5	112.2	118
GRMZM2G178517	152.8	143.6	142.4	159.9	147.8
GRMZM5G875732	83.4	88.2	89.3	88.7	173.4
**methyl indole-3-acetate interconversion**					
GRMZM2G022934[a]	3346.1	1403.8	500	101.6	8799.2
GRMZM2G046558[b]	4329.6	5738.9	5099.6	5581.9	4592.7
GRMZM2G060732	117.1	111.2	109.8	91.3	154.4
GRMZM2G060760	101.3	114.2	96	102	118.1
GRMZM2G085402[a]	476.1	355.3	295.7	305.2	649.7
GRMZM2G085939	108.9	101.1	104.6	102.9	82.5
GRMZM2G100716	254.4	318.9	388.6	419.9	774.5
GRMZM2G168299	996.6	959.4	1012.3	1334.8	1159.5
GRMZM2G174315[a]	377.1	501	380	959	826
**GA12 biosynthesis**					
GRMZM2G059308[a]	1993.6	3027	2477	1028.6	838.8
GRMZM2G093195[a]	95.7	220.5	336	216.7	72.3
**gibberellin inactivation I (2β-hydroxylation)**					
GRMZM2G022679[a]	542.5	289.3	217.8	377.8	1122.9
GRMZM2G051619[a]	1610.7	721	685.9	614.3	1998.2
GRMZM2G078798	84.5	88.4	98.3	79	80.4
GRMZM2G121700[b]	94.8	100.5	98.7	104.1	152
GRMZM2G147882	362.1	406.4	548.9	1087.1	546.3
GRMZM2G155686[a]	247.3	127.4	108.3	111.1	234
GRMZM2G427618	76.5	77.9	79.1	76.7	120.6
**brassinosteroid biosynthesis II**					
GRMZM2G009681	119.5	106.3	96.5	132.7	106.2
GRMZM2G017678	162	148.7	137.8	178.2	209.4
GRMZM2G031169	75.8	79.9	88	141.4	194.9
GRMZM2G031311	138.9	135.5	132.1	161	140

GRMZM2G033555	5652.9	7145.3	9292.1	11727.9	12421.3
GRMZM2G034069	1790.1	1910.1	1863.1	1169.4	1149.5
GRMZM2G040397[a]	611.8	739.9	756	635.6	1318.3
GRMZM2G042179	159.8	156.6	139.3	164.4	200.8
GRMZM2G044027[b]	253.5	193.8	175.1	234.7	309
GRMZM2G044281	144	162.4	127.9	158.2	90.1
GRMZM2G052336[a,b]	73.4	119.2	295.7	1083.6	428.3
GRMZM2G052357	785.4	872.1	739.9	608.3	1082.1
GRMZM2G057328	1517.3	1194.5	1276.5	1203.5	1473.6
GRMZM2G068917	628.2	409.3	454.2	303.9	338
GRMZM2G069708	271.6	281.6	367.3	427.1	409.7
GRMZM2G077531	194.1	193.2	231.8	269.9	226.5
GRMZM2G107076[a]	91.7	156.5	387.5	995.7	1725.4
GRMZM2G108570[a]	392.8	392.5	443.6	327.5	919.4
GRMZM2G109589[a]	145.6	116.4	107.1	183	84.3
GRMZM2G110558	626	674.4	592.1	1096.5	2054.7
GRMZM2G110881	114.5	119.8	150.8	212.2	127.1
GRMZM2G123652	247.9	256.7	279.4	357.3	671.9
GRMZM2G124434	395.3	244.4	209.8	300.6	565.5
GRMZM2G138410	173	199.7	245.1	192.8	257
GRMZM2G138907	176.3	143.7	135.4	169.7	117.6
GRMZM2G143235	197.4	112.7	133.8	137.9	182.8
GRMZM2G145460	139.7	150.9	150.9	142.1	106.9
GRMZM2G149224	199.5	206.9	179.7	145.9	74
GRMZM2G150541	337.1	289.3	314.3	398.7	389.9
GRMZM2G154532[b]	627.5	678.9	881.2	1288	972.3
GRMZM2G165357	158.3	145.3	144.3	141	228.4
GRMZM2G166767	1599.7	1861.6	2168.4	1973.7	2413.5
GRMZM2G170336	8180.9	9132.8	8184.9	6000.7	5626.6
GRMZM2G170812	333.4	353.5	649.3	851	513
GRMZM2G171662	1166.7	1204.3	1225.6	1377.2	1253.1
GRMZM2G179685	107.1	130.8	209.7	173.2	345.4
GRMZM2G179981	554	347.5	275.7	365.4	221.4
GRMZM2G180922	631.2	614.8	661.4	652	1031.7
GRMZM2G300412	135.7	104.1	92.3	96.8	164.2
GRMZM2G347717	146.6	134.7	143.8	205	317.7

GRMZM2G370048	135.1	162.7	158.5	152.1	300
GRMZM2G381473[a]	364.8	413	498.8	854.3	306.2
GRMZM2G468439[a]	199.1	147.7	156.3	189.9	824.1
GRMZM2G469523	2872.9	2978.7	3426.9	3411.9	4367.2
**abscisic acid biosynthesis**					
GRMZM2G124175	93.4	106.7	108.1	115.1	95.6
GRMZM2G141473[a]	72.9	77	102.7	765.2	179.9
GRMZM2G141535[a]	297.9	598.2	750.8	1532.8	1941.3
GRMZM2G446858	80.9	83.9	84.5	78.9	129.9
**cytokinins-O-glucoside biosynthesis**					
GRMZM2G004858[a]	90.2	92.9	85.3	385.6	72.1
GRMZM2G022101	167.3	106.2	104.4	99.8	137.7
GRMZM2G022242	314.7	357.2	421.3	277	358.8
GRMZM2G031138	92.1	123.5	149.7	131.6	126.3
GRMZM2G036409	134.2	104.4	93.8	117	115.1
GRMZM2G041699[a]	135.9	122.4	87.1	120.7	390.2
GRMZM2G043295	288	259.7	248.8	254.9	225
GRMZM2G058314	438.4	434.5	475.8	439	555.6
GRMZM2G061321	76.2	108.5	126.4	95.8	71.1
GRMZM2G063042	113.6	104.9	106	136.3	89.7
GRMZM2G073376	182.5	169.6	186.8	235.2	214.9
GRMZM2G082249[a]	158	196.7	239.3	297.3	673.8
GRMZM2G083935	72.3	90.9	121.9	95	65.2
GRMZM2G085054	706.3	548.5	396.1	409.7	767.4
GRMZM2G095280	440.5	299.1	254.1	227.1	260.5
GRMZM2G096412	72	87.1	134.2	82.4	134.2
GRMZM2G098667	414.4	359.1	352.8	314.6	320.3
GRMZM2G113794[a]	350.8	537.8	542.5	476.1	981.7
GRMZM2G118657	68.5	71.9	84.2	73.4	70.6
GRMZM2G122072	136.6	149.7	119.5	121.8	136
GRMZM2G142873[b]	196.5	224.8	239	200.3	188.9
GRMZM2G156127	179.1	212.9	359.2	239	163.4
GRMZM2G159404	215.6	196.7	203.7	244.9	295
GRMZM2G160452	207.8	277.9	257.8	294.2	215
GRMZM2G160523[a]	94.3	95.2	91.6	102.9	586.6
GRMZM2G161335	729.9	721.3	777.7	975.9	747.1

GRMZM2G165390[a]	447.9	390.9	412.5	776.8	3421.3
GRMZM2G173926	66.9	72.9	82.4	64.2	71.8
GRMZM2G304712	148.2	178.7	194.6	170.6	206.9
GRMZM2G363554[a]	853.8	690.2	792.1	337.2	170.7
GRMZM2G426415	84.6	120	132.7	174.2	153
GRMZM2G440902	87.6	94.9	110.6	104.8	78.2
GRMZM5G832805	74.8	78	93.4	133.5	152.1
**cytokinins degradation**					
GRMZM2G146644[a]	1502.9	1596.9	1706.3	846.6	420.2
GRMZM2G167220	103	105.8	113.9	101.6	121.3
GRMZM2G348452[a]	1039.1	1353.8	1170.6	429.5	618.8
GRMZM5G817173[a]	489.1	897.5	1297	572.2	1263.4
**ethylene biosynthesis from methionine**					
GRMZM2G006480[a]	69.5	122.2	237	126	252.7
GRMZM2G013448	265.2	215.4	137.5	270	393.8
GRMZM2G018006	145	138.6	138.7	107.8	68.6
GRMZM2G026131	137.8	132.4	140.8	152.7	168.8
GRMZM2G029135[a]	360.5	422.5	554.8	824.5	108.7
GRMZM2G033799[b]	1165.8	946.2	919.1	859.9	1077.1
GRMZM2G053999[b]	3028.9	3931.2	3965.8	3922.1	2975.5
GRMZM2G054361[a]	305.3	347.1	215.2	334.8	1066.7
GRMZM2G067265[b]	1161.5	1202.3	1052.5	1325	1949.1
GRMZM2G088064[a,b]	4767.7	6458.7	9210.4	12133.4	3546.9
GRMZM2G094712[b]	95.6	105.2	105.5	158.3	84.5
GRMZM2G107639[a,b]	457.1	652	1155.1	1384	692.4
GRMZM2G117198[a,b]	16000.8	18729.9	18788.1	23193.1	9959.9
GRMZM2G117230	70.4	75.1	72.4	78.2	155
GRMZM2G120302	99.5	103.6	109.4	123.5	128.8
GRMZM2G120563[b]	4605.6	5819.6	7347.5	4568.4	1953.1
GRMZM2G126732[a]	142.2	241.9	234.4	211.8	1136.2
GRMZM2G164405	90.4	96.1	113	130	227.3
GRMZM2G170595[a]	234.6	212.9	180.4	242.9	92
GRMZM2G174145[a]	747.7	949.4	744.6	869	409.3
GRMZM2G400604	158.5	198.7	163.2	88.2	94.8
GRMZM2G455945	245.1	235.5	227.3	301.5	400.7
GRMZM5G828630	137.8	148.5	130	187.9	134.7

GRMZM5G840582	236.6	244.9	329.3	172.2	135.4

The average intensities from the array experiments for all the constitutively expressed transcripts belonging to the hormone-associated pathways with at least one transcript meeting the two-fold cutoff as highlighted by Pathway Tools Omics Viewer (http://pathway.gramene.org/MAIZE/expression.html).

[a] Genes with two-fold difference between at least two stages.

[b] The protein was detected in the mass spectrometry data.

Table S6 Microarray intensities for transcription factor transcripts that exhibit differential accumulation during anther development.

Transcription factors scored as increased expression

Gene ID	Family	Stage	Relative intensity compared to previous stage
GRMZM2G151689	C3H	0.25mm	TURN ON
GRMZM2G124037	ERF	0.25mm	2.797966102
GRMZM2G069082	ERF	0.25mm	2.739469579
GRMZM2G139372	bHLH	0.25mm	2.435033259
GRMZM2G162481	WOX	0.25mm	2.379084967
GRMZM2G006578	bZIP	0.25mm	2.326327158
GRMZM2G113779	SBP	0.25mm	2.219683695
GRMZM2G069146	ERF	0.25mm	2.057844691
GRMZM2G137046	bZIP	0.40mm	TURN ON
GRMZM2G099522	MIKC	0.4mm	6.059807516
GRMZM2G095299	DBB	0.4mm	3.143856921
GRMZM2G102161	MIKC	0.4mm	2.828297362
GRMZM2G095598	CO-like	0.4mm	2.573320719
GRMZM2G171942	C3H	0.4mm	2.013937282
GRMZM2G030458	GeBP	0.7mm	TURN ON
GRMZM2G031120	NAC	0.7mm	TURN ON
GRMZM2G171468	G2-like	0.7mm	TURN ON
GRMZM2G126018	SBP	0.7mm	TURN ON
GRMZM2G124540	G2-like	0.7mm	TURN ON
GRMZM2G097349	HD-ZIP	0.7mm	TURN ON
GRMZM2G065896	GATA	0.7mm	TURN ON
GRMZM2G018876	DBB	0.7mm	TURN ON
GRMZM2G075562	CO-like	0.7mm	TURN ON
GRMZM2G108933	WOX	0.7mm	TURN ON
GRMZM2G098557	SBP	0.7mm	TURN ON
GRMZM2G447551	CAMTA	0.7mm	TURN ON
GRMZM2G102218	YABBY	0.7mm	3.52274377
GRMZM2G048450	WRKY	0.7mm	2.69488189
GRMZM5G839518	bHLH	0.7mm	2.604166667
GRMZM2G171942	C3H	0.7mm	2.53183391
GRMZM2G173882	G2-like	0.7mm	2.42605364
GRMZM2G147346	MYB	0.7mm	2.337253961
GRMZM2G019446	bZIP	0.7mm	2.280073462
GRMZM2G384528	NF-YB	0.7mm	2.262172285
GRMZM2G088524	MYB_related	0.7mm	2.250614251
GRMZM2G312201	NAC	0.7mm	2.2395771
GRMZM2G139688	MYB	0.7mm	2.193548387
GRMZM2G006745	ERF	0.7mm	2.186853767
GRMZM2G080509	GATA	0.7mm	2.145267545
GRMZM2G129147	GRF	0.7mm	2.118106433
GRMZM2G156016	VOZ	0.7mm	2.085276482
GRMZM2G169270	SBP	0.7mm	2.084667378
GRMZM2G067743	GRF	0.7mm	2.073722785

GRMZM2G139688	MYB	0.7mm	2.030612245
GRMZM2G103315	Trihelix	0.7mm	2.021681416
GRMZM2G067702	G2-like	0.7mm	2.014676716
GRMZM2G119823	bHLH	1.0mm	TURN ON
GRMZM2G136887	MYB_related	1.0mm	TURN ON
GRMZM2G109509	Nin-like	1.0mm	TURN ON
GRMZM2G154029	bHLH	1.0mm	TURN ON
GRMZM2G021339	HD-ZIP	1.0mm	TURN ON
GRMZM2G037910	bZIP	1.0mm	TURN ON
GRMZM2G463730	FAR1	1.0mm	TURN ON
GRMZM2G167829	MYB	1.0mm	TURN ON
GRMZM2G371058	Dof	1.0mm	TURN ON
GRMZM2G123667	NAC	1.0mm	TURN ON
GRMZM2G159119	G2-like	1.0mm	TURN ON
GRMZM2G173372	bHLH	1.0mm	TURN ON
GRMZM2G105387	M-type	1.0mm	TURN ON
GRMZM2G042101	bHLH	1.0mm	TURN ON
GRMZM2G445575	bZIP	1.0mm	TURN ON
GRMZM2G005353	YABBY	1.0mm	TURN ON
GRMZM2G139284	MYB	1.0mm	TURN ON
GRMZM2G114688	C3H	1.0mm	TURN ON
GRMZM2G099319	TALE	1.0mm	TURN ON
GRMZM2G016649	Trihelix	1.0mm	TURN ON
GRMZM2G079632	NAC	1.0mm	TURN ON
GRMZM2G044408	M-type	1.0mm	TURN ON
GRMZM2G150680	MYB	1.0mm	TURN ON
GRMZM2G439903	NAC	1.0mm	TURN ON
GRMZM2G380377	ERF	1.0mm	TURN ON
GRMZM2G092137	bZIP	1.0mm	TURN ON
GRMZM2G111731	MYB	1.0mm	26.65572391
GRMZM2G098696	HSF	1.0mm	9.914179104
GRMZM2G072582	MIKC	1.0mm	7.677824268
GRMZM5G874163	ARF	1.0mm	6.92749658
GRMZM2G449681	WRKY	1.0mm	6.849389987
GRMZM2G139963	HD-ZIP	1.0mm	6.807491702
GRMZM2G120320	WRKY	1.0mm	6.524705435
GRMZM2G384339	HSF	1.0mm	6.482216708
GRMZM2G125653	WRKY	1.0mm	5.978388998
GRMZM2G353076	ZF-HD	1.0mm	5.799773756
GRMZM2G134279	MYB	1.0mm	5.663985702
GRMZM2G171781	MYB	1.0mm	5.424817518
GRMZM2G079185	LBD	1.0mm	5.332498748
GRMZM2G394941	Dof	1.0mm	5.094233999
GRMZM5G828396	bHLH	1.0mm	4.849272349
GRMZM2G170049	MYB	1.0mm	4.842062853
GRMZM2G113060	ERF	1.0mm	4.657912016
GRMZM2G088524	MYB_related	1.0mm	4.622543668
GRMZM2G091201	MYB_related	1.0mm	4.203002611
GRMZM2G012724	WRKY	1.0mm	4.091583971

GRMZM2G104078	NAC	1.0mm	4.086577181
GRMZM2G324999	WRKY	1.0mm	3.989321789
GRMZM2G001223	MYB_related	1.0mm	3.969014085
GRMZM2G428184	bZIP	1.0mm	3.958141909
GRMZM2G375307	Trihelix	1.0mm	3.94591029
GRMZM2G001048	B3	1.0mm	3.911111111
GRMZM2G159500	NAC	1.0mm	3.910761155
GRMZM2G158328	WRKY	1.0mm	3.819941917
GRMZM2G106204	YABBY	1.0mm	3.774496644
GRMZM2G102583	WRKY	1.0mm	3.77252907
GRMZM2G014653	NAC	1.0mm	3.763888889
GRMZM2G148693	MIKC	1.0mm	3.758303887
GRMZM2G003466	ERF	1.0mm	3.678592375
GRMZM2G132367	HD-ZIP	1.0mm	3.661455201
GRMZM2G056600	HD-ZIP	1.0mm	3.66068168
GRMZM2G035405	ARF	1.0mm	3.645961077
GRMZM2G529859	YABBY	1.0mm	3.484215142
GRMZM2G171781	MYB	1.0mm	3.478658537
GRMZM2G060918	WRKY	1.0mm	3.470039947
GRMZM2G149347	C3H	1.0mm	3.45785877
GRMZM2G134073	NAC	1.0mm	3.411278195
GRMZM2G061408	WRKY	1.0mm	3.377542549
GRMZM2G301089	bHLH	1.0mm	3.372400756
GRMZM2G173862	bHLH	1.0mm	3.341553637
GRMZM2G024973	GRAS	1.0mm	3.333010492
GRMZM2G379005	GATA	1.0mm	3.322447292
GRMZM2G017087	TALE	1.0mm	3.317948718
GRMZM2G110582	M-type	1.0mm	3.301038062
GRMZM2G115070	MYB_related	1.0mm	3.270484981
GRMZM2G005624	HD-ZIP	1.0mm	3.254452926
GRMZM2G173882	G2-like	1.0mm	3.225521162
GRMZM2G160971	ERF	1.0mm	3.216624685
GRMZM2G157197	C2H2	1.0mm	3.201649175
GRMZM2G155662	Whirly	1.0mm	3.167141747
GRMZM2G068973	NAC	1.0mm	3.142911153
GRMZM2G020805	TCP	1.0mm	3.111872146
GRMZM2G097349	HD-ZIP	1.0mm	3.110945644
GRMZM2G314546	HB-PHD	1.0mm	3.06959136
GRMZM2G094892	bHLH	1.0mm	3.041726619
GRMZM2G160565	MIKC	1.0mm	2.998381877
GRMZM2G117193	G2-like	1.0mm	2.972972973
GRMZM2G125596	B3	1.0mm	2.966811414
GRMZM2G017606	SRS	1.0mm	2.946017699
GRMZM2G148074	HD-ZIP	1.0mm	2.902867174
GRMZM2G083886	GeBP	1.0mm	2.855906563
GRMZM2G179802	HSF	1.0mm	2.849860982
GRMZM2G139369	NF-X1	1.0mm	2.839322034
GRMZM2G134545	Dof	1.0mm	2.820720059
GRMZM2G098557	SBP	1.0mm	2.8203125

GRMZM2G169654	RAV	1.0mm	2.805497925
GRMZM2G116557	ARF	1.0mm	2.799946964
GRMZM2G096600	CPP	1.0mm	2.786065704
GRMZM2G106276	HD-ZIP	1.0mm	2.753188776
GRMZM2G152862	MIKC	1.0mm	2.74408284
GRMZM2G016649	Trihelix	1.0mm	2.732813607
GRMZM2G003514	MIKC	1.0mm	2.69541779
GRMZM2G153454	bHLH	1.0mm	2.674684305
GRMZM2G431156	MYB	1.0mm	2.632331283
GRMZM2G060544	LBD	1.0mm	2.604942339
GRMZM5G853392	GRF	1.0mm	2.571656051
GRMZM2G074124	YABBY	1.0mm	2.559023066
GRMZM2G171569	ERF	1.0mm	2.555205047
GRMZM2G178741	HD-ZIP	1.0mm	2.554880694
GRMZM2G104268	FAR1	1.0mm	2.541237113
GRMZM2G004641	TALE	1.0mm	2.521609538
GRMZM2G034638	TCP	1.0mm	2.47923588
GRMZM2G171365	MIKC	1.0mm	2.476977729
GRMZM2G126170	HB-other	1.0mm	2.473348229
GRMZM2G085678	ERF	1.0mm	2.473019518
GRMZM2G017349	bHLH	1.0mm	2.465884079
GRMZM2G116557	ARF	1.0mm	2.455823293
GRMZM2G052616	GATA	1.0mm	2.451843044
GRMZM2G041415	MYB	1.0mm	2.44200627
GRMZM2G000171	bZIP	1.0mm	2.408484654
GRMZM2G171179	ERF	1.0mm	2.377276115
GRMZM5G880268	Dof	1.0mm	2.36645525
GRMZM2G079470	GRAS	1.0mm	2.360888889
GRMZM2G113078	AP2	1.0mm	2.356396867
GRMZM2G148087	WRKY	1.0mm	2.343028229
GRMZM2G386163	NAC	1.0mm	2.328735632
GRMZM2G122076	HD-ZIP	1.0mm	2.32782292
GRMZM2G171179	ERF	1.0mm	2.322962963
GRMZM2G137510	MIKC	1.0mm	2.319758673
GRMZM2G166946	TCP	1.0mm	2.315142198
GRMZM2G336533	NAC	1.0mm	2.313117066
GRMZM2G064630	MYB	1.0mm	2.306837319
GRMZM2G126957	NF-YA	1.0mm	2.302362205
GRMZM2G113098	GATA	1.0mm	2.285059978
GRMZM2G151542	ERF	1.0mm	2.283093054
GRMZM2G103647	bZIP	1.0mm	2.26869092
GRMZM2G087741	TALE	1.0mm	2.25413403
GRMZM2G027333	C2H2	1.0mm	2.239792131
GRMZM2G164591	ERF	1.0mm	2.235989717
GRMZM2G098904	bZIP	1.0mm	2.223463687
GRMZM2G172621	B3	1.0mm	2.221378976
GRMZM2G141219	AP2	1.0mm	2.205665025
GRMZM2G024851	bZIP	1.0mm	2.196601942
GRMZM5G812774	BES1	1.0mm	2.193208593

GRMZM2G088309	YABBY	1.0mm	2.191432396
GRMZM2G142962	HD-ZIP	1.0mm	2.181132075
GRMZM2G049159	GRAS	1.0mm	2.167137809
GRMZM2G404973	GATA	1.0mm	2.166331321
GRMZM2G136266	bZIP	1.0mm	2.164710485
GRMZM2G306935	C3H	1.0mm	2.152099105
GRMZM2G027563	bHLH	1.0mm	2.142410016
GRMZM2G160687	MIKC	1.0mm	2.14229249
GRMZM2G097059	MIKC	1.0mm	2.139388343
GRMZM2G172657	GRAS	1.0mm	2.135502324
GRMZM2G379179	Trihelix	1.0mm	2.131596695
GRMZM2G310368	ERF	1.0mm	2.125984252
GRMZM2G069082	ERF	1.0mm	2.123264312
GRMZM2G082318	GeBP	1.0mm	2.115752829
GRMZM2G027976	bZIP	1.0mm	2.111402359
GRMZM2G086277	C2H2	1.0mm	2.101631117
GRMZM2G163200	GATA	1.0mm	2.083616918
GRMZM2G093305	C2H2	1.0mm	2.078774617
GRMZM2G087955	MYB	1.0mm	2.076335878
GRMZM2G093305	C2H2	1.0mm	2.075077399
GRMZM2G030710	ARF	1.0mm	2.071456462
GRMZM2G158162	B3	1.0mm	2.062742223
GRMZM2G531738	MYB_related	1.0mm	2.061936495
GRMZM2G102845	ARF	1.0mm	2.061165049
GRMZM2G162739	NAC	1.0mm	2.059100742
GRMZM2G032336	CAMTA	1.0mm	2.057898187
GRMZM2G010669	MIKC	1.0mm	2.056254626
GRMZM5G829103	NF-YA	1.0mm	2.043197937
GRMZM2G129428	C2H2	1.0mm	2.039702233
GRMZM2G148723	bHLH	1.0mm	2.022844688
GRMZM2G479110	ARR-B	1.0mm	2.021772939
GRMZM2G151763	WRKY	1.0mm	2.021327014
GRMZM2G320827	Trihelix	1.0mm	2.013457427

Transcription factors decreased in expression

Gene ID	Family	Stage	Relative intensity compared to previous stage
GRMZM2G005624	HD-ZIP	0.25mm	0.159622444
GRMZM2G087741	TALE	0.25mm	0.26545916
GRMZM2G529859	YABBY	0.25mm	0.283557919
GRMZM2G017087	TALE	0.25mm	0.28549975
GRMZM2G160565	MIKC	0.25mm	0.289216096
GRMZM2G134279	MYB	0.25mm	0.326057453
GRMZM2G091201	MYB_related	0.25mm	0.335834516
GRMZM2G003514	MIKC	0.25mm	0.348528015
GRMZM2G074543	YABBY	0.25mm	0.371785078
GRMZM2G120320	WRKY	0.25mm	0.387650872
GRMZM2G072582	MIKC	0.25mm	0.403132729

GRMZM2G353076	ZF-HD	0.25mm	0.414042553
GRMZM2G005353	YABBY	0.25mm	0.41617357
GRMZM2G028151	AP2	0.25mm	0.419765446
GRMZM2G068973	NAC	0.25mm	0.422173145
GRMZM2G108865	Dof	0.25mm	0.435992707
GRMZM2G054795	YABBY	0.25mm	0.455377574
GRMZM2G159431	TALE	0.25mm	0.460368641
GRMZM2G162739	NAC	0.25mm	0.462047961
GRMZM2G097349	HD-ZIP	0.25mm	0.462427746
GRMZM2G169654	RAV	0.25mm	0.468571761
GRMZM2G099522	MIKC	0.25mm	0.48069615
GRMZM2G177693	C2H2	0.25mm	0.48503937
GRMZM2G033570	EIL	0.25mm	0.486866792
GRMZM2G430522	NAC	0.25mm	0.492150706
GRMZM2G159500	NAC	0.25mm	0.493050133
GRMZM2G030458	GeBP	0.4mm	TURN OFF
GRMZM2G171468	G2-like	0.4mm	TURN OFF
GRMZM2G124540	G2-like	0.4mm	TURN OFF
GRMZM2G159119	G2-like	0.4mm	TURN OFF
GRMZM2G018254	GRAS	0.4mm	TURN OFF
GRMZM2G169580	Trihelix	0.4mm	TURN OFF
GRMZM2G005624	HD-ZIP	0.4mm	TURN OFF
GRMZM2G003514	MIKC	0.4mm	TURN OFF
GRMZM2G529859	YABBY	0.4mm	0.274272016
GRMZM2G069082	ERF	0.4mm	0.325882688
GRMZM2G160565	MIKC	0.4mm	0.387619323
GRMZM2G124037	ERF	0.4mm	0.440029077
GRMZM2G087741	TALE	0.4mm	0.455888744
GRMZM2G134279	MYB	0.4mm	0.472077697
GRMZM2G068973	NAC	0.4mm	0.480435237
GRMZM2G017087	TALE	0.4mm	0.481589714
GRMZM2G119823	bHLH	0.7mm	TURN OFF
GRMZM2G136887	MYB_related	0.7mm	TURN OFF
GRMZM2G080731	bZIP	0.7mm	TURN OFF
GRMZM2G162481	WOX	0.7mm	TURN OFF
GRMZM2G307119	ERF	0.7mm	TURN OFF
GRMZM2G099319	TALE	0.7mm	TURN OFF
GRMZM2G145041	MYB_related	0.7mm	TURN OFF
GRMZM2G370863	ZF-HD	0.7mm	TURN OFF
GRMZM2G145579	bHLH	0.7mm	0.154879494
GRMZM5G829103	NF-YA	0.7mm	0.225928623
GRMZM5G805685	B3	0.7mm	0.262887847
GRMZM2G169654	RAV	0.7mm	0.33937687
GRMZM2G158328	WRKY	0.7mm	0.354982818
GRMZM2G159500	NAC	0.7mm	0.363272311
GRMZM2G180190	LFY	0.7mm	0.365514392
GRMZM2G116557	ARF	0.7mm	0.378880739
GRMZM2G061408	WRKY	0.7mm	0.393756129
GRMZM2G045431	bHLH	0.7mm	0.400946814

GRMZM2G060216	bZIP	0.7mm	0.401126485
GRMZM2G027298	C3H	0.7mm	0.404302671
GRMZM2G386163	NAC	0.7mm	0.412713472
GRMZM5G803308	MYB	0.7mm	0.413376459
GRMZM2G060216	bZIP	0.7mm	0.416708271
GRMZM2G127857	MYB	0.7mm	0.417561592
GRMZM2G004957	HD-ZIP	0.7mm	0.417779898
GRMZM2G310368	ERF	0.7mm	0.423335745
GRMZM5G882527	bHLH	0.7mm	0.423863162
GRMZM2G120320	WRKY	0.7mm	0.431205441
GRMZM2G098813	LFY	0.7mm	0.447590661
GRMZM2G123900	Dof	0.7mm	0.448290184
GRMZM2G097683	SRS	0.7mm	0.460297507
GRMZM2G174284	bZIP	0.7mm	0.463846385
GRMZM2G139963	HD-ZIP	0.7mm	0.468666667
GRMZM2G133331	bZIP	0.7mm	0.469170579
GRMZM2G301089	bHLH	0.7mm	0.483369883
GRMZM2G178741	HD-ZIP	0.7mm	0.487521151
GRMZM2G453001	STAT	0.7mm	0.494759473
GRMZM2G134545	Dof	0.7mm	0.498534799
GRMZM2G065374	bHLH	1.0mm	TURN OFF
GRMZM2G327189	Dof	1.0mm	TURN OFF
GRMZM2G153333	GRAS	1.0mm	TURN OFF
GRMZM2G002915	HD-ZIP	1.0mm	TURN OFF
GRMZM2G470307	MYB	1.0mm	TURN OFF
GRMZM2G312201	NAC	1.0mm	TURN OFF
GRMZM2G034563	G2-like	1.0mm	0.043339578
GRMZM2G102218	YABBY	1.0mm	0.090284694
GRMZM2G096709	GRF	1.0mm	0.105003817
GRMZM2G100146	C2H2	1.0mm	0.122588993
GRMZM2G338259	ARF	1.0mm	0.129574468
GRMZM2G393433	NAC	1.0mm	0.14172688
GRMZM2G378580	ARF	1.0mm	0.188027274
GRMZM5G804893	NF-YB	1.0mm	0.189824773
GRMZM2G113779	SBP	1.0mm	0.206116839
GRMZM2G069146	ERF	1.0mm	0.214033019
GRMZM5G894234	NAC	1.0mm	0.228860936
GRMZM2G113127	NF-YC	1.0mm	0.24367077
GRMZM2G392516	GeBP	1.0mm	0.25477707
GRMZM5G887286	C2H2	1.0mm	0.259254482
GRMZM2G006493	C3H	1.0mm	0.264980491
GRMZM2G041223	GRF	1.0mm	0.280744068
GRMZM2G076272	TALE	1.0mm	0.284058522
GRMZM2G352159	ARF	1.0mm	0.286424355
GRMZM2G050939	C2H2	1.0mm	0.29821718
GRMZM2G159357	HB-other	1.0mm	0.304162725
GRMZM2G102514	BES1	1.0mm	0.315651626
GRMZM2G033413	bZIP	1.0mm	0.317846656
GRMZM2G065506	GeBP	1.0mm	0.318018665

GRMZM2G039828	bZIP	1.0mm	0.318021631
GRMZM2G701689	CPP	1.0mm	0.319008759
GRMZM2G019446	bZIP	1.0mm	0.320781313
GRMZM2G133169	GRAS	1.0mm	0.324274471
GRMZM2G123140	HD-ZIP	1.0mm	0.33064776
GRMZM2G154641	TALE	1.0mm	0.331354972
GRMZM2G129147	GRF	1.0mm	0.338819415
GRMZM2G069408	bHLH	1.0mm	0.343832021
GRMZM2G031120	NAC	1.0mm	0.34404708
GRMZM2G053503	ERF	1.0mm	0.347585662
GRMZM2G702026	ARF	1.0mm	0.353558926
GRMZM2G156016	VOZ	1.0mm	0.35942492
GRMZM2G120740	C3H	1.0mm	0.366255144
GRMZM2G479110	ARR-B	1.0mm	0.369612069
GRMZM2G109987	HD-ZIP	1.0mm	0.369684607
GRMZM2G048450	WRKY	1.0mm	0.373132346
GRMZM2G174917	ERF	1.0mm	0.380319497
GRMZM2G312201	NAC	1.0mm	0.383005255
GRMZM2G126957	NF-YA	1.0mm	0.38499385
GRMZM2G003944	TCP	1.0mm	0.386550308
GRMZM2G064541	NAC	1.0mm	0.386563416
GRMZM2G022213	C2H2	1.0mm	0.388605442
GRMZM2G173425	LSD	1.0mm	0.389802875
GRMZM2G096759	MYB_related	1.0mm	0.391697493
GRMZM2G061487	ERF	1.0mm	0.392430279
GRMZM2G055204	ERF	1.0mm	0.392656808
GRMZM2G341747	CAMTA	1.0mm	0.395641646
GRMZM2G089406	MYB_related	1.0mm	0.398830007
GRMZM2G479110	ARR-B	1.0mm	0.401297947
GRMZM2G431157	C2H2	1.0mm	0.404473439
GRMZM2G456568	NAC	1.0mm	0.405565979
GRMZM2G174240	GeBP	1.0mm	0.406111536
GRMZM2G431157	C2H2	1.0mm	0.406652807
GRMZM2G370777	MIKC	1.0mm	0.413241293
GRMZM2G389567	bHLH	1.0mm	0.415374241
GRMZM2G125777	NAC	1.0mm	0.417694933
GRMZM2G031001	NAC	1.0mm	0.417739483
GRMZM2G071907	WRKY	1.0mm	0.417923959
GRMZM2G009530	GATA	1.0mm	0.419946174
GRMZM2G174776	NF-YC	1.0mm	0.421550355
GRMZM2G151407	WRKY	1.0mm	0.421917808
GRMZM5G805026	WOX	1.0mm	0.422074604
GRMZM2G396451	GATA	1.0mm	0.426806613
GRMZM2G114461	FAR1	1.0mm	0.427480916
GRMZM2G050590	E2F/DP	1.0mm	0.427574171
GRMZM2G164735	BBR-BPC	1.0mm	0.431788653
GRMZM2G064197	G2-like	1.0mm	0.438966877
GRMZM2G379608	NAC	1.0mm	0.442261104
GRMZM2G416652	MYB	1.0mm	0.447083775

GRMZM2G126018	SBP	1.0mm	0.449616648
GRMZM2G101499	SBP	1.0mm	0.450732137
GRMZM2G013657	AP2	1.0mm	0.450952717
GRMZM2G125777	NAC	1.0mm	0.451251841
GRMZM2G003304	HD-ZIP	1.0mm	0.451289398
GRMZM2G070034	MIKC	1.0mm	0.451731375
GRMZM2G339848	HB-other	1.0mm	0.453190676
GRMZM2G169316	MYB	1.0mm	0.454652256
GRMZM2G033138	HB-other	1.0mm	0.456330081
GRMZM2G030744	bHLH	1.0mm	0.459872458
GRMZM2G095598	CO-like	1.0mm	0.460066393
GRMZM2G338259	ARF	1.0mm	0.463735361
GRMZM2G403620	MYB	1.0mm	0.465581051
GRMZM2G032336	CAMTA	1.0mm	0.465858328
GRMZM2G179049	NAC	1.0mm	0.4660523
GRMZM2G092091	bHLH	1.0mm	0.469780609
GRMZM2G028980	ARF	1.0mm	0.470108696
GRMZM2G130854	WRKY	1.0mm	0.472826087
GRMZM2G081812	C2H2	1.0mm	0.475670308
GRMZM2G060216	bZIP	1.0mm	0.476190476
GRMZM2G006871	GeBP	1.0mm	0.477103301
GRMZM2G139815	WRKY	1.0mm	0.479763861
GRMZM2G067743	GRF	1.0mm	0.481185031
GRMZM2G150262	C3H	1.0mm	0.483062331
GRMZM2G129428	C2H2	1.0mm	0.483926423
GRMZM2G025685	HSF	1.0mm	0.489446388
GRMZM2G061906	bHLH	1.0mm	0.490433577
GRMZM2G125239	GeBP	1.0mm	0.493167325
GRMZM2G384528	NF-YB	1.0mm	0.493708609
GRMZM2G069009	C3H	1.0mm	0.495503999
GRMZM2G095239	MYB_related	1.0mm	0.49656035

Transcription factors that are turned ON/OFF or UP/DOWN-regulated by at least two fold compared to previous stage. Gene identifiers, gene families, stages when differential transcript accumulation occurs and relative intensity of transcripts compared to prior stages are listed.

Table S7 Meiotic gene expression at five stages of early anther development.

MaizeGdbProteinID	MaizeGdbProteinName	0.15mm	0.25mm	0.4mm	0.7mm	1.0mm
AC198518.3_FG002	Sterile alpha motif (SAM) domain-containing protein	ON	ON	ON	ON	ON
AC207628.4_FG005	Porin/voltage-dependent anion-selective channel protein, alpha amylase activity	OFF	ON	ON	ON	ON
AC231745.1_FGT004	Protein-tyrosine sulfotransferase†	ON	ON	ON	ON	ON
GRMZM2G000397	Calcium-binding EF hand family protein	ON	ON	ON	ON	ON
GRMZM2G001803	ZIP metal ion transporter family	ON	ON	ON	ON	ON
GRMZM2G002765	UBA/THIF-type NAD/FAD binding fold	ON	ON	ON	ON	ON
GRMZM2G004455	RNA recognition motif 2	ON	ON	ON	ON	ON
GRMZM2G005036	Mitochondrial processing peptidase, alpha subunit	ON	ON	ON	ON	ON
GRMZM2G005374	Splicing factor 3b, subunit 4	ON	ON	ON	ON	ON
GRMZM2G006452	RecF/RecN/SMC protein, N-terminal	ON	ON	ON	ON	ON
GRMZM2G006452	RecF/RecN/SMC protein, N-terminal	ON	ON	ON	ON	ON
GRMZM2G006452	Tropomyosin	ON	ON	ON	ON	ON
GRMZM2G006452	RecF/RecN/SMC protein, N-terminal	ON	ON	ON	ON	ON
GRMZM2G006468	BBD: bifunctional nuclease in wound defense	ON	ON	ON	ON	ON
GRMZM2G007300	Ubiquitin carrier protein	ON	ON	ON	ON	ON
GRMZM2G008226	Trehalose-6-phosphate synthase	ON	ON	OFF	ON	OFF
GRMZM2G011078	Secretory carrier membrane protein, SC3, transport	ON	ON	ON	ON	ON
GRMZM2G012030	Vinculin/alpha-catenin	ON	ON	ON	ON	ON
GRMZM2G016602	protein recA	ON	ON	ON	ON	ON
GRMZM2G019596	Vamp/synaptobrevin-associated protein 27-2; major sperm protein	ON	ON	ON	ON	ON
GRMZM2G019596	Vamp/synaptobrevin-associated protein 27-2; major sperm protein	ON	ON	ON	ON	ON
GRMZM2G020974	DNA breaking-rejoining enzyme, catalytic core	ON	ON	ON	ON	ON
GRMZM2G021270	hypothetical protein LOC100193866	ON	ON	ON	ON	ON
GRMZM2G021270	DNA topoisomerase, type IIA, subunit A or C-termin	ON	ON	ON	ON	ON
GRMZM2G024739	Cryptochrome/DNA photolyase, class 1 conserved sit	ON	ON	ON	ON	ON
GRMZM2G028369	Chorismate mutase precursor	ON	ON	ON	ON	ON
GRMZM2G028640	Cupin superfamily enzyme	ON	ON	ON	ON	ON
GRMZM2G028763	Membrane related protein-like	ON	ON	ON	ON	ON
GRMZM2G029385	Chaperone (DnaJ); mitochondrial import inner membrane translocase subunit TIM14	ON	ON	ON	ON	ON
GRMZM2G030523	Proliferating cell nuclear antigen, PCNA	ON	ON	ON	ON	ON
GRMZM2G032003	UTP--glucose-1-phosphate uridylyltransferase†	ON	ON	ON	ON	ON
GRMZM2G032562	SCF ubiquitin ligase, Skp1	ON	ON	ON	ON	ON

	component; SKP1-like protein 1B (mediates cell cycle)					
GRMZM2G034631	Tyrosylprotein sulfotransferase	ON	ON	ON	ON	ON
GRMZM2G035996	Shaggy kinase homolog Fragment	ON	ON	ON	ON	OFF
GRMZM2G036765	Aspartate decarboxylase-like fold	ON	ON	ON	ON	ON
GRMZM2G039094	MATH domain containing protein	ON	ON	ON	ON	ON
GRMZM2G041418	NADH-dehydrogenase (ubiquinone),	OFF	ON	OFF	ON	ON
GRMZM2G042477	Monoglyceride lipase isoform 2-like, partial (90%)	ON	ON	ON	ON	ON
GRMZM2G043509	Endonuclease/Exonuclease/phosphatase family; DNaseI-like	ON	ON	ON	ON	ON
GRMZM2G044011	eRF1 domain 3	ON	ON	ON	ON	ON
GRMZM2G046055	Histone H2A	ON	ON	ON	ON	ON
GRMZM2G047204	Peptidyl-prolyl glycoprotein; histidine kinase	ON	ON	ON	ON	ON
GRMZM2G050329	Major Facilitator Superfamily with SPX (SYG1/Pho81/XPR1)	ON	ON	ON	ON	ON
GRMZM2G050684	Predicted membrane protein, contains two CBS domains	ON	ON	ON	ON	ON
GRMZM2G050684	CBS domain containing protein	ON	ON	ON	ON	ON
GRMZM2G052403	hypothetical protein LOC100383700	ON	ON	ON	ON	ON
GRMZM2G054115	Alliinase EGF-like domain; Pyridoxal phosphate (PLP)-dependent transferases †	ON	ON	ON	ON	ON
GRMZM2G055807	Restriction endonuclease, type I, R subunit/Type I	ON	ON	ON	ON	ON
GRMZM2G055807	DNA repair and transcription factor XPB1	ON	ON	ON	ON	ON
GRMZM2G056075	DNA mismatch repair protein MSH2 (MUS1)	ON	ON	ON	ON	ON
GRMZM2G057652	Protein of unknown function DUF1751, integral memb	ON	ON	OFF	OFF	ON
GRMZM2G058954	ATPase, AAA+ type, core	ON	ON	ON	ON	ON
GRMZM2G058954	DNA repair protein RAD51/RHP55, single stranded DNA repair	ON	ON	ON	ON	ON
GRMZM2G060394	SKP1-like protein 1A	ON	ON	OFF	ON	ON
GRMZM2G061023	RuvA domain 2-like	ON	ON	ON	ON	ON
GRMZM2G062761	MAP kinase, conserved site	ON	ON	ON	ON	ON
GRMZM2G062914	Serine/threonine protein kinase-related	ON	ON	ON	ON	ON
GRMZM2G063316	SET domain-containing protein,Rubisco methyltransferase family protein	ON	ON	ON	ON	ON
GRMZM2G063961	Serine/threonine-protein kinase SAPK4	ON	ON	ON	ON	ON
GRMZM2G064868	Putative 5-3 exonuclease	ON	ON	ON	ON	ON
GRMZM2G064868	Zinc finger, CCHC-type	ON	ON	ON	ON	ON
GRMZM2G070047	Ubiquitin-conjugating enzyme/RWD-like	OFF	OFF	OFF	OFF	OFF
GRMZM2G070639	Kinesin-like protein	OFF	OFF	OFF	OFF	ON
GRMZM2G071304	ATP dependent DNA ligase, central	ON	ON	ON	ON	ON
GRMZM2G071304	ATP dependent DNA ligase,	ON	ON	ON	ON	ON

	central					
GRMZM2G071630	Glyceraldehyde 3-phosphate dehydrogenase (GAPC3)	ON	ON	ON	ON	ON
GRMZM2G072088	Unknown function, localized to plasma membrane	ON	ON	OFF	ON	ON
GRMZM2G074082	Shugoshin, N-terminal	ON	ON	OFF	ON	ON
GRMZM2G074818	Rad21/Rec8 like protein, N-terminal	ON	ON	ON	ON	ON
GRMZM2G074818	Rad21/Rec8 like protein, N-terminal	ON	ON	ON	ON	ON
GRMZM2G074818	Rad21/Rec8 like protein, N-terminal	ON	ON	ON	ON	ON
GRMZM2G077823	Arginine:serine-rich splicing factor SC35	ON	ON	ON	ON	ON
GRMZM2G079613	Tetratricopeptide TPR-1	ON	ON	ON	ON	ON
GRMZM2G082214	Phosphoribosyl-AMP cyclohydrolase	ON	ON	ON	ON	ON
GRMZM2G083394	DCD domain protein - involved in development and cell death during HR response	ON	ON	ON	ON	ON
GRMZM2G083475	Prefoldin	ON	ON	ON	ON	ON
GRMZM2G083975	Splicing factor RSZ33 (RSZ33)	ON	ON	ON	ON	ON
GRMZM2G084762	DNA repair and recombination, RecA-like	ON	ON	ON	ON	ON
GRMZM2G088162	hypothetical protein LOC100276374	ON	ON	ON	ON	ON
GRMZM2G090152	Core-2/I-Branching enzyme, beta-1,6-N-acetylglucosaminyltransferase, role in salt stress.	ON	ON	ON	ON	ON
GRMZM2G090262	C-terminal phosphatase-like 4; NLI interacting factor-like phosphatase	ON	ON	ON	ON	ON
GRMZM2G091656	GRAS domain family, RGA-like 2	ON	ON	ON	ON	ON
GRMZM2G092232	Kinesin, motor region	ON	ON	ON	ON	ON
GRMZM2G093119	Rad21/Rec8 like protein, N-terminal	ON	ON	ON	ON	ON
GRMZM2G093623	DNA mismatch repair protein MutS, C-terminal	ON	ON	OFF	ON	ON
GRMZM2G096070	WD40 repeat-like	ON	ON	ON	ON	ON
GRMZM2G097605	Helicase-like, DEXD box c2 type	ON	ON	ON	ON	ON
GRMZM2G099080	SNX2b: sorting nexin 2B; phox domain containing protein	ON	ON	ON	ON	ON
GRMZM2G100103	Poor homologous synapsis 1 (PHS1) (meiosis chromosome pairing)	OFF	OFF	OFF	OFF	ON
GRMZM2G101613	Cyclin-dependent kinase inhibitor	OFF	ON	ON	ON	ON
GRMZM2G102088	Serine/threonine protein kinase-related	ON	ON	ON	ON	ON
GRMZM2G103287	Apospory-associated proteion aldose 1-epimerase	ON	ON	ON	ON	ON
GRMZM2G103287	Apospory-associated protein; aldose 1-epimerase	ON	ON	ON	ON	ON
GRMZM2G105250	AGO18a; Translation initiation factor 2C (eIF-2C)	ON	ON	ON	ON	OFF
GRMZM2G105387	MADS32; transcription factor; homolog of AtAGAMOUS-like 12	OFF	OFF	OFF	OFF	OFF

GRMZM2G108712	Proliferating cell nuclear antigen, PCNA	ON	ON	ON	ON	ON
GRMZM2G109383	Phosphoglucomutase, cytoplasmic	ON	ON	ON	ON	ON
GRMZM2G109383	Phosphoglucomutase, cytoplasmic 1 (PGM 1)(EC 5.4.2), phosphomannomutase	ON	ON	ON	ON	ON
GRMZM2G109496	Serine/threonine protein phosphatase	ON	ON	ON	ON	ON
GRMZM2G109618	DMC1 protein type B, DNA repair during meiotic recombination	OFF	OFF	OFF	OFF	OFF
GRMZM2G110212	DNA mismatch repair protein MutS-like, N-terminal	ON	ON	ON	ON	ON
GRMZM2G111436	Predicted 3'-5' exonuclease, Werner syndrome DNA helicase, nucleosidase	ON	ON	ON	ON	ON
GRMZM2G113228	Endonuclease III, 4Fe4S cluster, base excision repair	ON	ON	ON	ON	ON
GRMZM2G113967	NAF	ON	ON	OFF	ON	ON
GRMZM2G115013	RPA1a, replication protein required for meiotic DNA repair	ON	ON	ON	ON	ON
GRMZM2G115504	alpha / beta esterase; thioesterase; hydrolase	ON	ON	ON	ON	ON
GRMZM2G116243	1-acylglycerol-3-phosphate O-acyltransferase	ON	ON	ON	ON	ON
GRMZM2G116427	Metal ion binding; mitochondrial protein	ON	ON	ON	ON	ON
GRMZM2G119886	Arf GTPase activating protein	ON	ON	ON	ON	ON
GRMZM2G121210	DNA topoisomerase, type IIA, central	ON	ON	ON	ON	ON
GRMZM2G121262	Helix-hairpin-helix motif, class 2	ON	ON	ON	ON	ON
GRMZM2G121312	Leucine-rich repeat, typical subtype	ON	ON	ON	ON	ON
GRMZM2G121543	DNA repair protein RAD51	ON	ON	ON	ON	ON
GRMZM2G122306	Nucleic acid-binding, OB-fold-like	ON	ON	ON	ON	ON
GRMZM2G122965	P-loop ATP hydrolase, CH (Calponin Homology) domain, KINESIN motor domain containing	OFF	OFF	OFF	OFF	ON
GRMZM2G123776	Thioredoxin or redox protein	ON	ON	ON	ON	ON
GRMZM2G124365	Chorismate mutase	ON	ON	OFF	ON	ON
GRMZM2G124691	Cyclin	OFF	ON	OFF	ON	ON
GRMZM2G124718	Poly(ADP-ribose) polymerase, catalytic region	ON	ON	ON	ON	ON
GRMZM2G127893	hypothetical protein LOC100217125	ON	ON	OFF	ON	ON
GRMZM2G128771	GTPase Rab6/YPT6/Ryh1, small G protein superfamily	ON	ON	ON	ON	ON
GRMZM2G129175	Uncharacterized, contains transmembrane domain	ON	ON	ON	ON	ON
GRMZM2G129913	Spo11/DNA topoisomerase VI, subunit A	ON	ON	ON	ON	ON
GRMZM2G131443	Aldehyde dehydrogenase, conserved site	ON	ON	ON	ON	ON
GRMZM2G133006	MATE efflux family protein	ON	OFF	OFF	OFF	ON
GRMZM2G133048	Phosphatidate cytidylyltransferase†	ON	ON	ON	ON	ON
GRMZM2G133952	DNA mismatch repair, conserved	ON	ON	ON	ON	ON

	site					
GRMZM2G134502	Emp24/gp25L/p24 membrane trafficking; phospholipid binding; annexin	ON	ON	ON	ON	ON
GRMZM2G134708	Monodehydroascorbate/ferredoxin reductase	ON	ON	ON	ON	ON
GRMZM2G135073	Ste20-like serine/threonine protein kinase	ON	ON	ON	ON	ON
GRMZM2G135654	Ribosomal protein L7Ae/L30e/S12e/Gadd45	ON	ON	ON	ON	ON
GRMZM2G136455	GTPase Rab1/YPT1, small G protein superfamily, and related GTP-binding proteins	ON	ON	ON	ON	ON
GRMZM2G136710	Predicted K+/H+-antiporter	OFF	ON	ON	ON	ON
GRMZM2G137968	DSB repair, DNA helicase - NHEJ (non homologous end joining) double stranded break	ON	ON	ON	ON	ON
GRMZM2G138566	Calcium binding atopy-related autoantigen 1 (LOC10	ON	ON	ON	ON	ON
GRMZM2G138907	GDP-mannose 3\'5\' epimerase; dehydratase	ON	ON	ON	ON	ON
GRMZM2G139031	8-oxoguanine DNA glycosylase, N-terminal	ON	ON	ON	ON	ON
GRMZM2G140994	Rhomboid domain containing 1	ON	ON	ON	ON	ON
GRMZM2G143211	WD40 repeat	ON	ON	ON	ON	ON
GRMZM2G143462	Ubiquitin-associated/translation elongation factor	ON	ON	ON	ON	ON
GRMZM2G143590	Synaptonemal complex central region protein ZYP1-1	ON	ON	ON	ON	ON
GRMZM2G145034	Splicing factor, SPF45	ON	ON	ON	ON	ON
GRMZM2G145085	Transcription initiation factor IIF, large subunit (RAP74)	ON	ON	ON	ON	ON
GRMZM2G146206	Triosephosphate isomerase	ON	ON	OFF	ON	ON
GRMZM2G146490	BRCT	ON	ON	ON	ON	ON
GRMZM2G148249	DEAD-like helicase, N-terminal	OFF	OFF	OFF	OFF	OFF
GRMZM2G149289	hypothetical protein LOC100383454	ON	ON	ON	ON	ON
GRMZM2G153760	Galactosyltransferases	ON	ON	ON	ON	ON
GRMZM2G153899	Mitochondrial K+-H+ exchange-related	ON	ON	ON	ON	ON
GRMZM2G155806	Pumilio-family RNA binding repeat†	OFF	OFF	OFF	OFF	OFF
GRMZM2G157269	Acetyl-CoA synthetase	OFF	ON	OFF	OFF	OFF
GRMZM2G157505	EGG APPARATUS-1 protein	ON	ON	ON	ON	ON
GRMZM2G157817	Rad51, C-terminal	OFF	OFF	OFF	OFF	OFF
GRMZM2G159034	VH1-interacting kinase; ankyrin repeat containing	ON	ON	ON	ON	ON
GRMZM2G159307	ATPase associated with various cellular activities	OFF	ON	OFF	ON	OFF
GRMZM2G159724	NADP+-dependent malic enzyme	ON	ON	ON	ON	ON
GRMZM2G162968	Heat shock protein	ON	ON	ON	ON	ON
GRMZM2G163514	Leafbladeless1-like	ON	ON	ON	ON	ON
GRMZM2G164470	RXW8 protein, Lipase, GDSL	ON	ON	ON	ON	ON
GRMZM2G166597	Alpha-D-phosphohexomutase, alpha/beta/alpha I, II	ON	OFF	OFF	ON	ON

GRMZM2G166658	Serine/threonine protein kinase-related	ON	ON	ON	ON	ON
GRMZM2G169089	Acyl-CoA:diacylglycerol acyltransferase 1	ON	ON	ON	ON	OFF
GRMZM2G169709	Phosphoethanolamine N-methyltransferase	ON	ON	ON	ON	ON
GRMZM2G169931	Nonsense-mediated mRNA decay NMD3 family protein	OFF	ON	ON	ON	ON
GRMZM2G173428	FOG: RRM domain, CID11, nucleic acid binding	OFF	ON	OFF	ON	ON
GRMZM2G174574	Acyl-CoA synthetase	OFF	OFF	OFF	ON	OFF
GRMZM2G175676	hypothetical protein LOC100384862	ON	ON	ON	ON	ON
GRMZM2G175676	RNA recognition motif, RNP-1	ON	ON	ON	ON	ON
GRMZM2G177461	Myo-inositol-1-phosphate synthase, inositol-3-phosphate synthase	ON	ON	ON	ON	ON
GRMZM2G179215	Ras GTPase	ON	ON	ON	ON	ON
GRMZM2G300786	SWI1 / DYAD involved in meiotic recombination	ON	ON	OFF	ON	ON
GRMZM2G301405	hypothetical protein LOC100192986	ON	ON	ON	ON	ON
GRMZM2G310739	3-glucanase	ON	ON	OFF	ON	ON
GRMZM2G315176	DNA/RNA helicase, DEAD/DEAH box type, N-terminal	ON	ON	ON	ON	ON
GRMZM2G315726	LAG1 longevity assurance homolog 3, ASC1	ON	ON	ON	ON	ON
GRMZM2G315726	ASC1, WD-domain, sphingolipid biosynthesis, resists fungal infection	ON	ON	ON	ON	ON
GRMZM2G315769	Sodium:dicarboxylate symporter	ON	ON	ON	ON	ON
GRMZM2G324390	Keratin, type I	ON	ON	ON	ON	ON
GRMZM2G324781	SNF2 helicase domain-containing protein; DNA repair protein RAD16	ON	ON	ON	ON	ON
GRMZM2G329710	EBNA-1, FAS1 domain	ON	ON	ON	ON	ON
GRMZM2G336761	Zinc finger, C3HC4 type (RING finger), U-box	ON	ON	ON	ON	ON
GRMZM2G342807	Permeases (Mycobacterium smegmatis str. MC2 155)	ON	ON	ON	ON	ON
GRMZM2G347717	UDP-glucuronic acid decarboxylase, 3-beta-hydroxy-Delta(5)-steroid dehydrogenase	ON	ON	OFF	ON	ON
GRMZM2G349996	Eukaryotic translation initiation factor 1A	ON	ON	ON	ON	ON
GRMZM2G351810	bHLH transcription factor	OFF	ON	OFF	ON	ON
GRMZM2G372180	Calponin-like actin-binding	ON	ON	ON	ON	ON
GRMZM2G390896	Protein kinase-like	ON	ON	OFF	ON	OFF
GRMZM2G408768	14-3-3 protein	ON	ON	OFF	ON	ON
GRMZM2G410710	Prohibitin, Mitochondrial prohibitin complex protein 1, membrane, PHB3	ON	ON	ON	ON	ON
GRMZM2G416019	Leucine-rich repeat	OFF	OFF	OFF	OFF	OFF
GRMZM2G416069	RecF/RecN/SMC protein, N-terminal	OFF	OFF	OFF	OFF	ON
GRMZM2G425986	Endonuclease III-like, iron-sulphur	ON	ON	ON	ON	ON

	cluster loop					
GRMZM2G432075	ZmAGO121, AtAGO6 and AtAGO4 homolog (involved in RdDM) Zwille:pinhead-like	ON	ON	ON	ON	ON
GRMZM2G447447	Protein kinase-like	ON	ON	ON	ON	ON
GRMZM2G456570	SMCs flexible hinge	ON	ON	ON	ON	ON
GRMZM2G456626	Inositol 1,3,4-trisphosphate 5/6-kinase family protein	ON	ON	ON	ON	ON
GRMZM2G457370	AGO18b; similar to ZLL:PNH	OFF	OFF	OFF	OFF	ON
GRMZM2G458974	Ribosomal protein S7e family	OFF	ON	OFF	ON	OFF
GRMZM2G459702	tRNA pseudouridine synthase D (TruD)†	ON	ON	ON	ON	ON
GRMZM2G464382	Exo70 exocyst complex subunit†(exocytosis)	OFF	OFF	OFF	ON	OFF
GRMZM2G465553	Transcription elongation factor, IIS	ON	ON	ON	ON	ON
GRMZM2G468479	hypothetical protein LOC100217142	ON	ON	ON	ON	ON
GRMZM2G470438	Zinc finger, GRF-type	ON	ON	ON	ON	ON
GRMZM2G474929	Selenium-binding protein-like	ON	ON	ON	ON	ON
GRMZM2G547542	Malonyl CoA-acyl carrier protein transacylase	ON	ON	ON	ON	ON
GRMZM2G563190	NADH-dehydrogenase (ubiquinone) - FAD, NADP, NADPH binding, cytoplasm	ON	ON	ON	ON	OFF
GRMZM2G588241	FOG: Predicted E3 ubiquitin ligase, RHC1A	ON	ON	ON	ON	ON
GRMZM2G700757	RecA family protein, NTP binding, DNA repair, ssDNA binding	ON	ON	ON	ON	ON
GRMZM5G802801	HSP70	ON	ON	ON	ON	ON
GRMZM5G806358	FACT complex subunit SPT16 (Facilitates transcription via Pol II)	ON	ON	ON	ON	ON
GRMZM5G828630	Alanine aminotransferase	ON	ON	ON	ON	ON
GRMZM5G831712	Poly [ADP-ribose] polymerase 1 (PARP-1)(EC 2.4.2.3	ON	ON	ON	ON	ON
GRMZM5G832378	Transcription initiation factor IIA gamma chain (TFIIA-gamma)	OFF	OFF	OFF	ON	ON
GRMZM5G841619	Tryptophan synthase alpha	ON	ON	ON	ON	ON
GRMZM5G842071	Laccase 1, complete (oxygen binding - quinone associated)	OFF	OFF	OFF	OFF	OFF
GRMZM5G856297	Meiotic recombination protein	OFF	OFF	OFF	OFF	OFF
GRMZM5G870959	somatic embryogenesis receptor-like kinase1	ON	ON	ON	ON	ON
GRMZM5G878823	Kinesin 1-like	ON	ON	ON	ON	ON
GRMZM5G889299	UTP--glucose-1-phosphate uridylyltransferase	ON	ON	ON	ON	ON
GRMZM5G890815	PAP/OAS1 substrate-binding domain superfamily, nucleotidyltransferase	OFF	OFF	OFF	ON	ON
GRMZM5G892926	Not determined - may be psuedogene	ON	ON	ON	ON	ON
GRMZM5G899800	hypothetical protein LOC100272324	ON	ON	ON	ON	ON

Expression patterns of genes classified as meiotic genes, including known genes involved in maize meiosis, maize homologs of meiotic genes in other flowering plants, and 222 annotated genes that are all mis-regulated in *ameiotic1* anthers were scored. Gene identifiers, Gene Ontology and their expressions (ON or OFF) are listed.

Tables S8-S15

Available for download at http://www.g3journal.org/lookup/suppl/doi:10.1534/g3.113.009738/-/DC1

Table S8 Candidate cell-type enriched transcripts as shown in Figure 7 Venn diagrams. This file contains the feature numbers, Protein IDs, and log-fold enrichment of the hundreds or thousands of transcripts demonstrated to be significantly enriched in a particular anther cell type at the 0.7 mm stage of anther development, just after the formation of the presumptive middle layer and tapetum cell types by periclinal division of the SPL. The log-fold enrichment is a quantification by microarray hybridization of tissue-specific enrichment compared to whole anthers of the 0.7 mm stage. Towards the right side of the file (column S-U) there is a key describing the comparisons that made up each list. Abbreviations: MLT, middle layer & tapetum; EE, epidermis & endothecium; AR, archesporial cells; C & V, connective tissue and vasculature.

Table S9 Pluripotency markers. Transcripts rapidly quenched after the primordium stage (DOWN in 0.25 mm compared to 0.15mm, and DOWN again in 0.4 mm compared to 0.25 mm) were assigned as pluripotency markers if they were also upregulated in 0.4 mm *mac1* anthers. The logic is that pluripotency markers should have elevated expression in 0.4 mm *mac1* compared to 0.4 mm wild type because there are some remnant pluripotent L2-d cells in 0.4 mm *mac1* anthers that are absent in wild type, because all L2-d cells now have acquired a specific cell fate.

Table S10 Transcripts enriched in or specific to wild type over *mac1* at 0.2 mm. At the 0.2 mm stage, the AR are just starting to secrete the MAC1 protein ligand into the surrounding L2-d cells, triggering a periclinal division forming presumptive SPL and EN cell types while at the same time repressing AR proliferation. MAC1 suppression of AR proliferation could be a direct action of this protein on AR cells or an indirect consequence of L2-d specification initiated by MAC1. This list contains the early set of genes that are downregulated in the *mac1* mutant, potentially representing early markers for EN and SPL cell type identity or factors responsible for somatic niche construction.

Table S11 Transcripts enriched in or specific to *mac1* over wild type at 0.2 mm. This list contains the first upregulated (or 'de-repressed') genes in the *mac1* mutant when compared to wild type anthers of the same stage. Genes in this list may relate to L2-d pluripotency, which would normally be repressed upon construction of the SPL and EN.

Table S12 Transcripts enriched in or specific to wild type over *mac1* at 0.4 mm. At 0.4 mm in *mac1*, there is still no somatic bilayer formed – no SPL or EN cell types – rather a single layer of pluripotent L2-d remain uncommitted to a particular cellular fate. In addition, the AR population has doubled its size compared to wild type. This list contains the transcripts downregulated or missing in *mac1* mutant anthers at this stage, which may represent SPL or EN markers or genes normally turned off in AR cells to prevent their overproliferation.

Table S13 Transcripts enriched in or specific to *mac1* over wild type at 0.4 mm. This list may contain AR markers (as there are excess AR in *mac1* compared to wild type) and pluripotency markers for the L2-d cell type.

Table S14 Transcripts enriched in or specific to *mac1* over wild type at both 0.2 and 0.4 mm stages. compared to fertile anthers at the same stages. This list shows the overlap between the very early 0.2 mm anther and early 0.4 mm anther comparisons between *mac1* and wild type. This set may describe genes that are upregulated immediately downstream of *mac1* signal perception. Transcription factors are in bold.

Table S15 Transcripts enriched in or specific to wild type over *mac1* at both 0.2 and 0.4 mm stages. compared to fertile anthers at the same stages. Like Table S14, this list shows the overlap between 0.2 and 0.4 mm *mac1* vs. wild type comparisons – particularly the genes enriched in the *mac1* mutant. This set may contain genes downregulated immediately after *mac1* signal perception. Transcription factors are in bold.
