# Supplementary material for: Transcriptomes and Proteomes Define Gene Expression Progression in Pre-meiotic Maize Anthers
Source: G3 (Bethesda). 2014 Jun 1;4(6):993–1010. doi: 10.1534/g3.113.009738 (PMC4065268; doi:10.1534/g3.113.009738)
Supplement: Supporting Information [file supp_4.6.993_FigureS1.pdf]

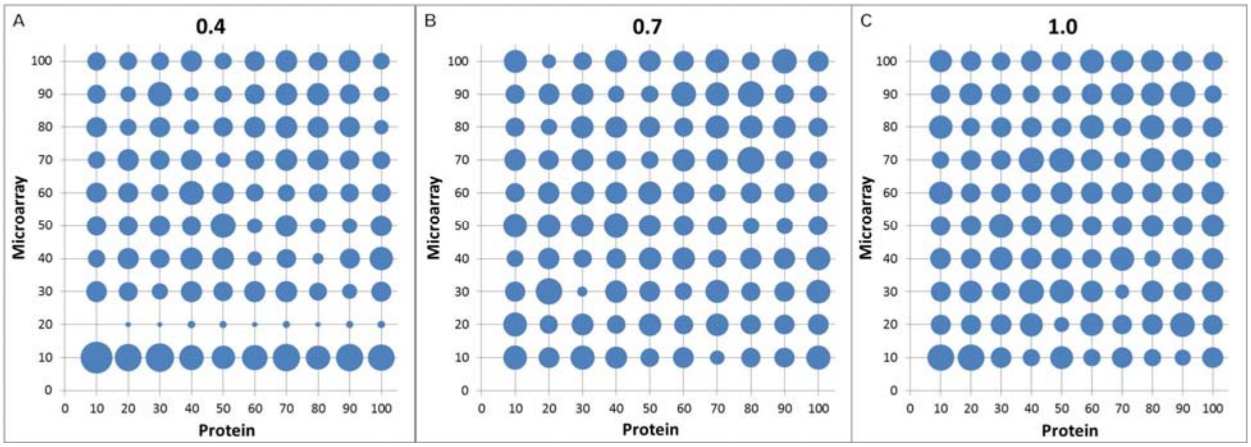

Figure S1 Decile comparisons of transcriptomes and proteomes at threes stages. Comparison of protein and transcript abundances at three developmental stages: 0.4, 0.7, and 1.0 mm. Data were divided into ten bins based on abundance. Circle diameters reflect the number of protein-transcript matches in each of the 100 bins.
