# Supplementary material for: Transcriptomes and Proteomes Define Gene Expression Progression in Pre-meiotic Maize Anthers
Source: G3 (Bethesda). 2014 Jun 1;4(6):993–1010. doi: 10.1534/g3.113.009738 (PMC4065268; doi:10.1534/g3.113.009738)
Supplement: Supporting Information [file supp_4.6.993_FigureS2.pdf]

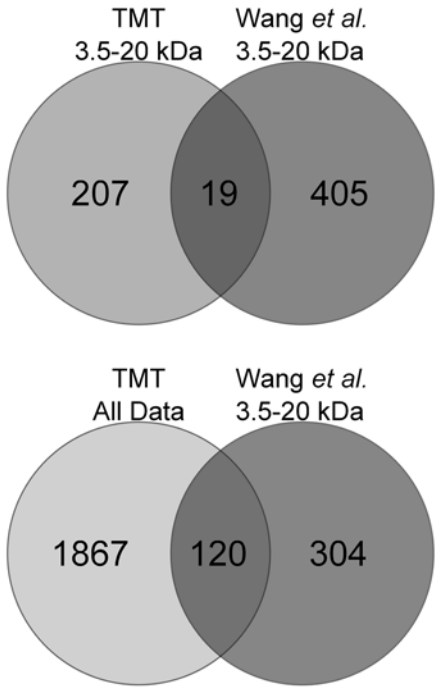

Figure S2 Comparison between the early anther proteomes with previously-published meiotic-stage small protein proteomes. Venn diagrams illustrating shared and unshared 3.5 – 20 kDa proteins identified in the present proteomes (*TMT*, including 0.4, 0.7, and 1.0 mm anther sizes) and published 1.5 plus 2.0 mm meiotic anther proteomes (Wang *et al*. 2010a). The upper Venn includes only proteins identified in the 3.5 – 20 kDa gel slices for both proteomes; the lower Venn examines proteins from any size class in the current data compared to the 3.5 – 20 kDa from Wang *et al.*
