# Supplementary material for: Transcriptomes and Proteomes Define Gene Expression Progression in Pre-meiotic Maize Anthers
Source: G3 (Bethesda). 2014 Jun 1;4(6):993–1010. doi: 10.1534/g3.113.009738 (PMC4065268; doi:10.1534/g3.113.009738)
Supplement: Supporting Information [file supp_4.6.993_TableS1.pdf]

Table S1 Comparative anther staging in *Arabidopsis thaliana* and *Zea mays*.

Arabidopsis				Maize		
Anther stage */floral stage*	Cell types present	Hours	Stamen length (µm) */anther*	Cell types + anther event	Hours	Anther length (µm)
1 / *1-5*	L1 L2 L3	96	<10/ no data	L1 L2 primordium	96	0-120
2 / *6*	EPI/AR	30	<20/no data	EPI/L2-d/AR specification	56	70-220
3 / *7*	EPI/SPL/AR	24	<50/ no data	EPI/EN/SPL/AR	42	180-280
				Anticlinal mitotic proliferation	36	280-550
4 / *8*	EPI/EN/ML/TA/AR Specification done	24	55-60/*40*	EPI/EN/ML/TA/AR Specification done	24	550-700
				Anticlinal somatic cell proliferation	48	700-1200
5 / *9*	PMC mature	60	150/*120*	PMC mature	60	1000-1500
6 / *10*	Prophase 1 meiosis	12	300/*240*	Prophase 1 meiosis	48	1500-2000
Total hours		**246**			**410**	

Staging information for *A. thaliana* is taken from Smyth *et al*. (1990) and Sanders *et al*. (1999). Maize data are from Kelliher and Walbot (2011, 2012).
