# Supplementary material for: Transcriptomes and Proteomes Define Gene Expression Progression in Pre-meiotic Maize Anthers
Source: G3 (Bethesda). 2014 Jun 1;4(6):993–1010. doi: 10.1534/g3.113.009738 (PMC4065268; doi:10.1534/g3.113.009738)
Supplement: Supporting Information [file supp_4.6.993_TableS4.pdf]

Table S4 Quantitative real-time PCR results for 19 genes sampled from the microarray results.

Feature Number	MaizeGDB Protein ID	MaizeGDB Protein Name	Microarray Expression Pattern 0.4 mm	0.7 mm	1.0 mm	qRT-PCR Average C(t) 0.4 mm	0.7 mm	1.0 mm	Pattern Verified
6513	N/A	N/A	OFF	ON	ON	26.7	26.6	26.6	No
10373	GRMZM2G113967	NAF	OFF	ON	ON	23.0	22.5	23.4	No
33683	GRMZM2G180916	Protein kinase-like	OFF	ON	ON	31.2	30.8	31.5	No
37851	GRMZM2G166665	lipopolysaccharide-modifying protein	OFF	ON	ON	28.4	28.5	26.9	No
44169	GRMZM2G131715	N/A	OFF	ON	ON	32.7	*32.8	32.8	No
18879	N/A	N/A	OFF	ON	ON	*28.1	25.1	23.8	Yes
29	GRMZM2G175761	Zinc finger, LIM-type	OFF	ON	ON	21.6	22.3	22.5	No
24021	GRMZM2G170457	hypothetical protein LOC100384355	ON	ON	ON	21.7	21.2	21.3	Yes
30914	GRMZM5G856777	Photosystem II CP43 chlorophyll apoprotein Precurs	ON	ON	ON	19.8	18.7	18.6	Yes
38273	GRMZM2G436092	N/A	ON	ON	ON	21.5	21.3	21.6	Yes
13365	GRMZM2G170120	Serine/threonine protein kinase, active site	ON	ON	ON	23.9	23.4	23.6	Yes
36944	GRMZM2G095892	LOC100281279	ON	ON	ON	23.2	23.6	24.1	Yes
5400	GRMZM2G166524	Leucine-rich repeat	ON	ON	ON	22.1	21.3	21.1	Yes
43905	GRMZM2G034882	Tropomyosin	ON	ON	ON	26.2	26.0	26.2	Yes
22801	GRMZM2G084779	K+ potassium transporter	OFF	OFF	OFF	28.8	28.6	28.6	No
34528	GRMZM2G130389	Serine/threonine protein kinase-related	OFF	OFF	OFF	**32.3	32.5	31.8	Yes
30365	GRMZM5G868296	Putative uncharacterized protein	OFF	OFF	OFF	30.6	31.1	31.1	Yes
39100	GRMZM2G111954	Aldo/keto reductase	OFF	OFF	ON	**30.1	*30.1	29.8	No
36385	GRMZM2G085872	CemA	OFF	OFF	ON	28.2	28.1	*28.3	No
33573	GRMZM2G134747	LOC100284448 (Cyanase)	ON	ON	ON	22.1	22.0	22.2	Yes

Feature Number	MaizeGDB Protein ID	Forward Primer Sequence	Reverse Primer Sequence
6513	N/A	GGCCATGAAACACCAGAAGG	AGGAAAGGTCACATGCACGA
10373	GRMZM2G113967	GACAGGCTATGATGGGGCAA	GCTGGGGTCGAGGATCTTG
33683	GRMZM2G180916	AATGTCTCGTGCTTGGCAGA	AGGCCAGCTTGGATTTGGAT
37851	GRMZM2G166665	TCCCCCTCCCTCTCTCTCTA	GCAGGACGGCAGGATACATA
44169	GRMZM2G131715	TGGCCGATCGAGCCTTTTT	ACGGTGGCAAGAATACAGCA
18879	N/A	TCTACACCTCCACGCTCGAC	GTCGCATACAACCACGGTCT
29	GRMZM2G175761	GAGAAGGGCAACTTCAGCC	TCTGAACGCACATGATCTCC
24021	GRMZM2G170457	CCCCTACTTCGATCTGGACA	TCTGAATGGAACCTGCTGTG
30914	GRMZM5G856777	TTCGCCAAATTACCCGAAG	ATTCAATTTGGCAGGCAGAA

38273	GRMZM2G436092	CGCAGTACGAGATTGGATGA	ACAGCACCAAATCCACACC
13365	GRMZM2G170120	AACCCGTCATCATTGTTGG	TCTGGTCTGTCTTGCAGGC
36944	GRMZM2G095892	CCAGTGTCTCAACTCAAGCG	CGGAGATCACGTAGAGGGG
5400	GRMZM2G166524	GGTTCTTGGGTTGGTGTCC	CCAGATCCATGTTCCCTCC
43905	GRMZM2G034882	ACCGAGGTCCTGAAGATGC	AATGCATCCAAACGAGGC
22801	GRMZM2G084779	CATAACAGACTGCTCCGGG	GAACAGAACCCTTCCATTTCC
34528	GRMZM2G130389	ACATGCATTGAAATTGGGC	AGCAAATCTCCATGGGTCC
30365	GRMZM5G868296	ACCTACGATTCCCTCACCG	CCCCAAATTTTCTTCCACG
39100	GRMZM2G111954	TCTGAACTGGCTGACCTGC	CTGCTACGACATGGGTTGG
36385	GRMZM2G085872	AACGAGGAGTGAATTGCTGC	TTTGAAGCTGATGAAGCGG
33573	GRMZM2G134747	GCTGGTGAGGAGGAGAAACA	CAGCAATCATGCCAGGTAGA

Candidate genes were chosen based on a pattern of expression using OFF or ON at 0.4, 0.7, and 1.0 mm whole anther stages as detailed under "Transcriptome Analysis – Data Analysis" in Materials and Methods. Patterns chosen to verify were: OffOnOn, OnOnOn, OffOffOff, and OffOffOn, relative to 0.4, 0.7, and 1.0 mm. Average $C_{(T)}$ values are shown using cyanase as an internal reference (Ma et al. 2007). Three biological samples and three technical replicates for each sample were used, except where indicated. Below, primer sequences for the transcripts examined in the qRT-PCR study. *One biological replicate excluded; **Two biological replicates excluded.
