# Supplementary material for: Transcriptomes and Proteomes Define Gene Expression Progression in Pre-meiotic Maize Anthers
Source: G3 (Bethesda). 2014 Jun 1;4(6):993–1010. doi: 10.1534/g3.113.009738 (PMC4065268; doi:10.1534/g3.113.009738)
Supplement: Supporting Information [file supp_4.6.993_TableS5.pdf]

Table S5 Average intensities of hormone-related pathways.

Pathways	0.15 mm	0.25 mm	0.4 mm	0.7 mm	1.0 mm
**jasmonic acid biosynthesis**					
GRMZM2G002959	554.4	633.2	657.8	778.4	1323.3
GRMZM2G009479	244.1	303.1	300.6	333.5	298.2
GRMZM2G014136	152.5	142.7	140.7	104.9	175.5
GRMZM2G015419	108.5	91.5	83.3	114.4	113.1
GRMZM2G033491	290.2	244.3	218.9	397.5	352.7
GRMZM2G052389	267.5	183.6	137.7	172.4	177.3
GRMZM2G072653[a]	608.7	548.6	607.2	496.7	2133.3
GRMZM2G077316[a,b]	1044.6	1204.2	1328.3	1646.8	3669.4
GRMZM2G082087	142	145.7	149.1	161.7	270.1
GRMZM2G087192[a]	122.2	281.7	214.6	187.1	457.6
GRMZM2G102760	89.8	87.1	78.3	86.9	76.2
GRMZM2G104843	94.4	104.9	99.7	90.6	84.3
GRMZM2G106250	87	84.6	87.2	97.9	129.4
GRMZM2G109056[a]	4517	4803.9	4290.5	4761	2369.7
GRMZM2G109130[a]	1303.8	764.7	386.6	379.6	1768.2
GRMZM2G110201[a,b]	1409.9	1317.7	1390.3	1891.3	845.2
GRMZM2G117357	312.4	248	204.1	222.2	203.2
GRMZM2G132903[a,b]	508	531.1	549.5	423.2	1741.4
GRMZM2G146885	879.3	945.2	1017.6	646	998.2
GRMZM2G148281	1458.8	1271.5	1320	1378.5	1407.8
GRMZM2G151087[a]	3503.3	3650	3714.4	2928.4	8280.9
GRMZM2G156712[a]	143.4	161.7	165.6	111.4	230.1
GRMZM2G156861[a]	1133.5	432.6	199	112.5	3820.4
GRMZM2G168404	171.1	153.1	154.5	143.5	85.6
GRMZM2G398500	333.2	312.8	393.7	237.2	273.7
GRMZM2G415793	3569.9	3047.6	3782.3	7002.8	5972
GRMZM2G459755	84.9	92.7	98.2	90.9	72.3
GRMZM5G862219[a]	2417.9	2288.7	2159.6	2941.8	497.3
GRMZM5G864319[b]	1252.7	1061.9	1092	1407.4	546
**IAA biosynthesis I**					
GRMZM2G022192	123.9	115.2	113.2	128.2	93.7
GRMZM2G066345	122.6	148.1	153	87.7	139.8

GRMZM2G111225	175.2	151	139.7	197.4	111.9
GRMZM2G111309[a]	181.9	174.4	170.6	109	224.3
GRMZM2G117614[a]	348.1	304.2	320.4	290.3	690.4
GRMZM2G124175	93.4	106.7	108.1	115.1	95.6
GRMZM2G141473[a]	72.9	77	102.7	765.2	179.9
GRMZM2G141535[a]	297.9	598.2	750.8	1532.8	1941.3
GRMZM2G159542	230.9	223.2	205.9	267.1	137
GRMZM2G169087	95.1	94.4	81.5	112.2	118
GRMZM2G178517	152.8	143.6	142.4	159.9	147.8
GRMZM5G875732	83.4	88.2	89.3	88.7	173.4
**methyl indole-3-acetate interconversion**					
GRMZM2G022934[a]	3346.1	1403.8	500	101.6	8799.2
GRMZM2G046558[b]	4329.6	5738.9	5099.6	5581.9	4592.7
GRMZM2G060732	117.1	111.2	109.8	91.3	154.4
GRMZM2G060760	101.3	114.2	96	102	118.1
GRMZM2G085402[a]	476.1	355.3	295.7	305.2	649.7
GRMZM2G085939	108.9	101.1	104.6	102.9	82.5
GRMZM2G100716	254.4	318.9	388.6	419.9	774.5
GRMZM2G168299	996.6	959.4	1012.3	1334.8	1159.5
GRMZM2G174315[a]	377.1	501	380	959	826
**GA12 biosynthesis**					
GRMZM2G059308[a]	1993.6	3027	2477	1028.6	838.8
GRMZM2G093195[a]	95.7	220.5	336	216.7	72.3
**gibberellin inactivation I (2β-hydroxylation)**					
GRMZM2G022679[a]	542.5	289.3	217.8	377.8	1122.9
GRMZM2G051619[a]	1610.7	721	685.9	614.3	1998.2
GRMZM2G078798	84.5	88.4	98.3	79	80.4
GRMZM2G121700[b]	94.8	100.5	98.7	104.1	152
GRMZM2G147882	362.1	406.4	548.9	1087.1	546.3
GRMZM2G155686[a]	247.3	127.4	108.3	111.1	234
GRMZM2G427618	76.5	77.9	79.1	76.7	120.6
**brassinosteroid biosynthesis II**					
GRMZM2G009681	119.5	106.3	96.5	132.7	106.2
GRMZM2G017678	162	148.7	137.8	178.2	209.4
GRMZM2G031169	75.8	79.9	88	141.4	194.9
GRMZM2G031311	138.9	135.5	132.1	161	140

GRMZM2G033555	5652.9	7145.3	9292.1	11727.9	12421.3
GRMZM2G034069	1790.1	1910.1	1863.1	1169.4	1149.5
GRMZM2G040397[a]	611.8	739.9	756	635.6	1318.3
GRMZM2G042179	159.8	156.6	139.3	164.4	200.8
GRMZM2G044027[b]	253.5	193.8	175.1	234.7	309
GRMZM2G044281	144	162.4	127.9	158.2	90.1
GRMZM2G052336[a,b]	73.4	119.2	295.7	1083.6	428.3
GRMZM2G052357	785.4	872.1	739.9	608.3	1082.1
GRMZM2G057328	1517.3	1194.5	1276.5	1203.5	1473.6
GRMZM2G068917	628.2	409.3	454.2	303.9	338
GRMZM2G069708	271.6	281.6	367.3	427.1	409.7
GRMZM2G077531	194.1	193.2	231.8	269.9	226.5
GRMZM2G107076[a]	91.7	156.5	387.5	995.7	1725.4
GRMZM2G108570[a]	392.8	392.5	443.6	327.5	919.4
GRMZM2G109589[a]	145.6	116.4	107.1	183	84.3
GRMZM2G110558	626	674.4	592.1	1096.5	2054.7
GRMZM2G110881	114.5	119.8	150.8	212.2	127.1
GRMZM2G123652	247.9	256.7	279.4	357.3	671.9
GRMZM2G124434	395.3	244.4	209.8	300.6	565.5
GRMZM2G138410	173	199.7	245.1	192.8	257
GRMZM2G138907	176.3	143.7	135.4	169.7	117.6
GRMZM2G143235	197.4	112.7	133.8	137.9	182.8
GRMZM2G145460	139.7	150.9	150.9	142.1	106.9
GRMZM2G149224	199.5	206.9	179.7	145.9	74
GRMZM2G150541	337.1	289.3	314.3	398.7	389.9
GRMZM2G154532[b]	627.5	678.9	881.2	1288	972.3
GRMZM2G165357	158.3	145.3	144.3	141	228.4
GRMZM2G166767	1599.7	1861.6	2168.4	1973.7	2413.5
GRMZM2G170336	8180.9	9132.8	8184.9	6000.7	5626.6
GRMZM2G170812	333.4	353.5	649.3	851	513
GRMZM2G171662	1166.7	1204.3	1225.6	1377.2	1253.1
GRMZM2G179685	107.1	130.8	209.7	173.2	345.4
GRMZM2G179981	554	347.5	275.7	365.4	221.4
GRMZM2G180922	631.2	614.8	661.4	652	1031.7
GRMZM2G300412	135.7	104.1	92.3	96.8	164.2
GRMZM2G347717	146.6	134.7	143.8	205	317.7

GRMZM2G370048	135.1	162.7	158.5	152.1	300
GRMZM2G381473[a]	364.8	413	498.8	854.3	306.2
GRMZM2G468439[a]	199.1	147.7	156.3	189.9	824.1
GRMZM2G469523	2872.9	2978.7	3426.9	3411.9	4367.2
**abscisic acid biosynthesis**					
GRMZM2G124175	93.4	106.7	108.1	115.1	95.6
GRMZM2G141473[a]	72.9	77	102.7	765.2	179.9
GRMZM2G141535[a]	297.9	598.2	750.8	1532.8	1941.3
GRMZM2G446858	80.9	83.9	84.5	78.9	129.9
**cytokinins-O-glucoside biosynthesis**					
GRMZM2G004858[a]	90.2	92.9	85.3	385.6	72.1
GRMZM2G022101	167.3	106.2	104.4	99.8	137.7
GRMZM2G022242	314.7	357.2	421.3	277	358.8
GRMZM2G031138	92.1	123.5	149.7	131.6	126.3
GRMZM2G036409	134.2	104.4	93.8	117	115.1
GRMZM2G041699[a]	135.9	122.4	87.1	120.7	390.2
GRMZM2G043295	288	259.7	248.8	254.9	225
GRMZM2G058314	438.4	434.5	475.8	439	555.6
GRMZM2G061321	76.2	108.5	126.4	95.8	71.1
GRMZM2G063042	113.6	104.9	106	136.3	89.7
GRMZM2G073376	182.5	169.6	186.8	235.2	214.9
GRMZM2G082249[a]	158	196.7	239.3	297.3	673.8
GRMZM2G083935	72.3	90.9	121.9	95	65.2
GRMZM2G085054	706.3	548.5	396.1	409.7	767.4
GRMZM2G095280	440.5	299.1	254.1	227.1	260.5
GRMZM2G096412	72	87.1	134.2	82.4	134.2
GRMZM2G098667	414.4	359.1	352.8	314.6	320.3
GRMZM2G113794[a]	350.8	537.8	542.5	476.1	981.7
GRMZM2G118657	68.5	71.9	84.2	73.4	70.6
GRMZM2G122072	136.6	149.7	119.5	121.8	136
GRMZM2G142873[b]	196.5	224.8	239	200.3	188.9
GRMZM2G156127	179.1	212.9	359.2	239	163.4
GRMZM2G159404	215.6	196.7	203.7	244.9	295
GRMZM2G160452	207.8	277.9	257.8	294.2	215
GRMZM2G160523[a]	94.3	95.2	91.6	102.9	586.6
GRMZM2G161335	729.9	721.3	777.7	975.9	747.1

GRMZM2G165390[a]	447.9	390.9	412.5	776.8	3421.3
GRMZM2G173926	66.9	72.9	82.4	64.2	71.8
GRMZM2G304712	148.2	178.7	194.6	170.6	206.9
GRMZM2G363554[a]	853.8	690.2	792.1	337.2	170.7
GRMZM2G426415	84.6	120	132.7	174.2	153
GRMZM2G440902	87.6	94.9	110.6	104.8	78.2
GRMZM5G832805	74.8	78	93.4	133.5	152.1
**cytokinins degradation**					
GRMZM2G146644[a]	1502.9	1596.9	1706.3	846.6	420.2
GRMZM2G167220	103	105.8	113.9	101.6	121.3
GRMZM2G348452[a]	1039.1	1353.8	1170.6	429.5	618.8
GRMZM5G817173[a]	489.1	897.5	1297	572.2	1263.4
**ethylene biosynthesis from methionine**					
GRMZM2G006480[a]	69.5	122.2	237	126	252.7
GRMZM2G013448	265.2	215.4	137.5	270	393.8
GRMZM2G018006	145	138.6	138.7	107.8	68.6
GRMZM2G026131	137.8	132.4	140.8	152.7	168.8
GRMZM2G029135[a]	360.5	422.5	554.8	824.5	108.7
GRMZM2G033799[b]	1165.8	946.2	919.1	859.9	1077.1
GRMZM2G053999[b]	3028.9	3931.2	3965.8	3922.1	2975.5
GRMZM2G054361[a]	305.3	347.1	215.2	334.8	1066.7
GRMZM2G067265[b]	1161.5	1202.3	1052.5	1325	1949.1
GRMZM2G088064[a,b]	4767.7	6458.7	9210.4	12133.4	3546.9
GRMZM2G094712[b]	95.6	105.2	105.5	158.3	84.5
GRMZM2G107639[a,b]	457.1	652	1155.1	1384	692.4
GRMZM2G117198[a,b]	16000.8	18729.9	18788.1	23193.1	9959.9
GRMZM2G117230	70.4	75.1	72.4	78.2	155
GRMZM2G120302	99.5	103.6	109.4	123.5	128.8
GRMZM2G120563[b]	4605.6	5819.6	7347.5	4568.4	1953.1
GRMZM2G126732[a]	142.2	241.9	234.4	211.8	1136.2
GRMZM2G164405	90.4	96.1	113	130	227.3
GRMZM2G170595[a]	234.6	212.9	180.4	242.9	92
GRMZM2G174145[a]	747.7	949.4	744.6	869	409.3
GRMZM2G400604	158.5	198.7	163.2	88.2	94.8
GRMZM2G455945	245.1	235.5	227.3	301.5	400.7
GRMZM5G828630	137.8	148.5	130	187.9	134.7

GRMZM5G840582	236.6	244.9	329.3	172.2	135.4

The average intensities from the array experiments for all the constitutively expressed transcripts belonging to the hormone-associated pathways with at least one transcript meeting the two-fold cutoff as highlighted by Pathway Tools Omics Viewer (http://pathway.gramene.org/MAIZE/expression.html).

[a] Genes with two-fold difference between at least two stages.

[b] The protein was detected in the mass spectrometry data.
