# Supplementary material for: Transcriptomes and Proteomes Define Gene Expression Progression in Pre-meiotic Maize Anthers
Source: G3 (Bethesda). 2014 Jun 1;4(6):993–1010. doi: 10.1534/g3.113.009738 (PMC4065268; doi:10.1534/g3.113.009738)
Supplement: Supporting Information [file supp_4.6.993_TableS6.pdf]

**Table S6 Microarray intensities for transcription factor transcripts that exhibit differential accumulation during anther development.**

**Transcription factors scored as increased expression**

| Gene ID | Family | Stage | Relative intensity compared to previous stage |
|---|---|---|---|
| GRMZM2G151689 | C3H | 0.25mm | TURN ON |
| GRMZM2G124037 | ERF | 0.25mm | 2.797966102 |
| GRMZM2G069082 | ERF | 0.25mm | 2.739469579 |
| GRMZM2G139372 | bHLH | 0.25mm | 2.435033259 |
| GRMZM2G162481 | WOX | 0.25mm | 2.379084967 |
| GRMZM2G006578 | bZIP | 0.25mm | 2.326327158 |
| GRMZM2G113779 | SBP | 0.25mm | 2.219683695 |
| GRMZM2G069146 | ERF | 0.25mm | 2.057844691 |
| GRMZM2G137046 | bZIP | 0.40mm | TURN ON |
| GRMZM2G099522 | MIKC | 0.4mm | 6.059807516 |
| GRMZM2G095299 | DBB | 0.4mm | 3.143856921 |
| GRMZM2G102161 | MIKC | 0.4mm | 2.828297362 |
| GRMZM2G095598 | CO-like | 0.4mm | 2.573320719 |
| GRMZM2G171942 | C3H | 0.4mm | 2.013937282 |
| GRMZM2G030458 | GeBP | 0.7mm | TURN ON |
| GRMZM2G031120 | NAC | 0.7mm | TURN ON |
| GRMZM2G171468 | G2-like | 0.7mm | TURN ON |
| GRMZM2G126018 | SBP | 0.7mm | TURN ON |
| GRMZM2G124540 | G2-like | 0.7mm | TURN ON |
| GRMZM2G097349 | HD-ZIP | 0.7mm | TURN ON |
| GRMZM2G065896 | GATA | 0.7mm | TURN ON |
| GRMZM2G018876 | DBB | 0.7mm | TURN ON |
| GRMZM2G075562 | CO-like | 0.7mm | TURN ON |
| GRMZM2G108933 | WOX | 0.7mm | TURN ON |
| GRMZM2G098557 | SBP | 0.7mm | TURN ON |
| GRMZM2G447551 | CAMTA | 0.7mm | TURN ON |
| GRMZM2G102218 | YABBY | 0.7mm | 3.52274377 |
| GRMZM2G048450 | WRKY | 0.7mm | 2.69488189 |
| GRMZM5G839518 | bHLH | 0.7mm | 2.604166667 |
| GRMZM2G171942 | C3H | 0.7mm | 2.53183391 |
| GRMZM2G173882 | G2-like | 0.7mm | 2.42605364 |
| GRMZM2G147346 | MYB | 0.7mm | 2.337253961 |
| GRMZM2G019446 | bZIP | 0.7mm | 2.280073462 |
| GRMZM2G384528 | NF-YB | 0.7mm | 2.262172285 |
| GRMZM2G088524 | MYB_related | 0.7mm | 2.250614251 |
| GRMZM2G312201 | NAC | 0.7mm | 2.2395771 |
| GRMZM2G139688 | MYB | 0.7mm | 2.193548387 |
| GRMZM2G006745 | ERF | 0.7mm | 2.186853767 |
| GRMZM2G080509 | GATA | 0.7mm | 2.145267545 |
| GRMZM2G129147 | GRF | 0.7mm | 2.118106433 |
| GRMZM2G156016 | VOZ | 0.7mm | 2.085276482 |
| GRMZM2G169270 | SBP | 0.7mm | 2.084667378 |
| GRMZM2G067743 | GRF | 0.7mm | 2.073722785 |

| | | | |
|---|---|---|---|
| GRMZM2G139688 | MYB | 0.7mm | 2.030612245 |
| GRMZM2G103315 | Trihelix | 0.7mm | 2.021681416 |
| GRMZM2G067702 | G2-like | 0.7mm | 2.014676716 |
| GRMZM2G119823 | bHLH | 1.0mm | TURN ON |
| GRMZM2G136887 | MYB_related | 1.0mm | TURN ON |
| GRMZM2G109509 | Nin-like | 1.0mm | TURN ON |
| GRMZM2G154029 | bHLH | 1.0mm | TURN ON |
| GRMZM2G021339 | HD-ZIP | 1.0mm | TURN ON |
| GRMZM2G037910 | bZIP | 1.0mm | TURN ON |
| GRMZM2G463730 | FAR1 | 1.0mm | TURN ON |
| GRMZM2G167829 | MYB | 1.0mm | TURN ON |
| GRMZM2G371058 | Dof | 1.0mm | TURN ON |
| GRMZM2G123667 | NAC | 1.0mm | TURN ON |
| GRMZM2G159119 | G2-like | 1.0mm | TURN ON |
| GRMZM2G173372 | bHLH | 1.0mm | TURN ON |
| GRMZM2G105387 | M-type | 1.0mm | TURN ON |
| GRMZM2G042101 | bHLH | 1.0mm | TURN ON |
| GRMZM2G445575 | bZIP | 1.0mm | TURN ON |
| GRMZM2G005353 | YABBY | 1.0mm | TURN ON |
| GRMZM2G139284 | MYB | 1.0mm | TURN ON |
| GRMZM2G114688 | C3H | 1.0mm | TURN ON |
| GRMZM2G099319 | TALE | 1.0mm | TURN ON |
| GRMZM2G016649 | Trihelix | 1.0mm | TURN ON |
| GRMZM2G079632 | NAC | 1.0mm | TURN ON |
| GRMZM2G044408 | M-type | 1.0mm | TURN ON |
| GRMZM2G150680 | MYB | 1.0mm | TURN ON |
| GRMZM2G439903 | NAC | 1.0mm | TURN ON |
| GRMZM2G380377 | ERF | 1.0mm | TURN ON |
| GRMZM2G092137 | bZIP | 1.0mm | TURN ON |
| GRMZM2G111731 | MYB | 1.0mm | 26.65572391 |
| GRMZM2G098696 | HSF | 1.0mm | 9.914179104 |
| GRMZM2G072582 | MIKC | 1.0mm | 7.677824268 |
| GRMZM5G874163 | ARF | 1.0mm | 6.92749658 |
| GRMZM2G449681 | WRKY | 1.0mm | 6.849389987 |
| GRMZM2G139963 | HD-ZIP | 1.0mm | 6.807491702 |
| GRMZM2G120320 | WRKY | 1.0mm | 6.524705435 |
| GRMZM2G384339 | HSF | 1.0mm | 6.482216708 |
| GRMZM2G125653 | WRKY | 1.0mm | 5.978388998 |
| GRMZM2G353076 | ZF-HD | 1.0mm | 5.799773756 |
| GRMZM2G134279 | MYB | 1.0mm | 5.663985702 |
| GRMZM2G171781 | MYB | 1.0mm | 5.424817518 |
| GRMZM2G079185 | LBD | 1.0mm | 5.332498748 |
| GRMZM2G394941 | Dof | 1.0mm | 5.094233999 |
| GRMZM5G828396 | bHLH | 1.0mm | 4.849272349 |
| GRMZM2G170049 | MYB | 1.0mm | 4.842062853 |
| GRMZM2G113060 | ERF | 1.0mm | 4.657912016 |
| GRMZM2G088524 | MYB_related | 1.0mm | 4.622543668 |
| GRMZM2G091201 | MYB_related | 1.0mm | 4.203002611 |
| GRMZM2G012724 | WRKY | 1.0mm | 4.091583971 |

| | | | |
|---|---|---|---|
| GRMZM2G104078 | NAC | 1.0mm | 4.086577181 |
| GRMZM2G324999 | WRKY | 1.0mm | 3.989321789 |
| GRMZM2G001223 | MYB_related | 1.0mm | 3.969014085 |
| GRMZM2G428184 | bZIP | 1.0mm | 3.958141909 |
| GRMZM2G375307 | Trihelix | 1.0mm | 3.94591029 |
| GRMZM2G001048 | B3 | 1.0mm | 3.911111111 |
| GRMZM2G159500 | NAC | 1.0mm | 3.910761155 |
| GRMZM2G158328 | WRKY | 1.0mm | 3.819941917 |
| GRMZM2G106204 | YABBY | 1.0mm | 3.774496644 |
| GRMZM2G102583 | WRKY | 1.0mm | 3.77252907 |
| GRMZM2G014653 | NAC | 1.0mm | 3.763888889 |
| GRMZM2G148693 | MIKC | 1.0mm | 3.758303887 |
| GRMZM2G003466 | ERF | 1.0mm | 3.678592375 |
| GRMZM2G132367 | HD-ZIP | 1.0mm | 3.661455201 |
| GRMZM2G056600 | HD-ZIP | 1.0mm | 3.66068168 |
| GRMZM2G035405 | ARF | 1.0mm | 3.645961077 |
| GRMZM2G529859 | YABBY | 1.0mm | 3.484215142 |
| GRMZM2G171781 | MYB | 1.0mm | 3.478658537 |
| GRMZM2G060918 | WRKY | 1.0mm | 3.470039947 |
| GRMZM2G149347 | C3H | 1.0mm | 3.45785877 |
| GRMZM2G134073 | NAC | 1.0mm | 3.411278195 |
| GRMZM2G061408 | WRKY | 1.0mm | 3.377542549 |
| GRMZM2G301089 | bHLH | 1.0mm | 3.372400756 |
| GRMZM2G173862 | bHLH | 1.0mm | 3.341553637 |
| GRMZM2G024973 | GRAS | 1.0mm | 3.333010492 |
| GRMZM2G379005 | GATA | 1.0mm | 3.322447292 |
| GRMZM2G017087 | TALE | 1.0mm | 3.317948718 |
| GRMZM2G110582 | M-type | 1.0mm | 3.301038062 |
| GRMZM2G115070 | MYB_related | 1.0mm | 3.270484981 |
| GRMZM2G005624 | HD-ZIP | 1.0mm | 3.254452926 |
| GRMZM2G173882 | G2-like | 1.0mm | 3.225521162 |
| GRMZM2G160971 | ERF | 1.0mm | 3.216624685 |
| GRMZM2G157197 | C2H2 | 1.0mm | 3.201649175 |
| GRMZM2G155662 | Whirly | 1.0mm | 3.167141747 |
| GRMZM2G068973 | NAC | 1.0mm | 3.142911153 |
| GRMZM2G020805 | TCP | 1.0mm | 3.111872146 |
| GRMZM2G097349 | HD-ZIP | 1.0mm | 3.110945644 |
| GRMZM2G314546 | HB-PHD | 1.0mm | 3.06959136 |
| GRMZM2G094892 | bHLH | 1.0mm | 3.041726619 |
| GRMZM2G160565 | MIKC | 1.0mm | 2.998381877 |
| GRMZM2G117193 | G2-like | 1.0mm | 2.972972973 |
| GRMZM2G125596 | B3 | 1.0mm | 2.966811414 |
| GRMZM2G017606 | SRS | 1.0mm | 2.946017699 |
| GRMZM2G148074 | HD-ZIP | 1.0mm | 2.902867174 |
| GRMZM2G083886 | GeBP | 1.0mm | 2.855906563 |
| GRMZM2G179802 | HSF | 1.0mm | 2.849860982 |
| GRMZM2G139369 | NF-X1 | 1.0mm | 2.839322034 |
| GRMZM2G134545 | Dof | 1.0mm | 2.820720059 |
| GRMZM2G098557 | SBP | 1.0mm | 2.8203125 |

| | | | |
|---|---|---|---|
| GRMZM2G169654 | RAV | 1.0mm | 2.805497925 |
| GRMZM2G116557 | ARF | 1.0mm | 2.799946964 |
| GRMZM2G096600 | CPP | 1.0mm | 2.786065704 |
| GRMZM2G106276 | HD-ZIP | 1.0mm | 2.753188776 |
| GRMZM2G152862 | MIKC | 1.0mm | 2.74408284 |
| GRMZM2G016649 | Trihelix | 1.0mm | 2.732813607 |
| GRMZM2G003514 | MIKC | 1.0mm | 2.69541779 |
| GRMZM2G153454 | bHLH | 1.0mm | 2.674684305 |
| GRMZM2G431156 | MYB | 1.0mm | 2.632331283 |
| GRMZM2G060544 | LBD | 1.0mm | 2.604942339 |
| GRMZM5G853392 | GRF | 1.0mm | 2.571656051 |
| GRMZM2G074124 | YABBY | 1.0mm | 2.559023066 |
| GRMZM2G171569 | ERF | 1.0mm | 2.555205047 |
| GRMZM2G178741 | HD-ZIP | 1.0mm | 2.554880694 |
| GRMZM2G104268 | FAR1 | 1.0mm | 2.541237113 |
| GRMZM2G004641 | TALE | 1.0mm | 2.521609538 |
| GRMZM2G034638 | TCP | 1.0mm | 2.47923588 |
| GRMZM2G171365 | MIKC | 1.0mm | 2.476977729 |
| GRMZM2G126170 | HB-other | 1.0mm | 2.473348229 |
| GRMZM2G085678 | ERF | 1.0mm | 2.473019518 |
| GRMZM2G017349 | bHLH | 1.0mm | 2.465884079 |
| GRMZM2G116557 | ARF | 1.0mm | 2.455823293 |
| GRMZM2G052616 | GATA | 1.0mm | 2.451843044 |
| GRMZM2G041415 | MYB | 1.0mm | 2.44200627 |
| GRMZM2G000171 | bZIP | 1.0mm | 2.408484654 |
| GRMZM2G171179 | ERF | 1.0mm | 2.377276115 |
| GRMZM5G880268 | Dof | 1.0mm | 2.36645525 |
| GRMZM2G079470 | GRAS | 1.0mm | 2.360888889 |
| GRMZM2G113078 | AP2 | 1.0mm | 2.356396867 |
| GRMZM2G148087 | WRKY | 1.0mm | 2.343028229 |
| GRMZM2G386163 | NAC | 1.0mm | 2.328735632 |
| GRMZM2G122076 | HD-ZIP | 1.0mm | 2.32782292 |
| GRMZM2G171179 | ERF | 1.0mm | 2.322962963 |
| GRMZM2G137510 | MIKC | 1.0mm | 2.319758673 |
| GRMZM2G166946 | TCP | 1.0mm | 2.315142198 |
| GRMZM2G336533 | NAC | 1.0mm | 2.313117066 |
| GRMZM2G064630 | MYB | 1.0mm | 2.306837319 |
| GRMZM2G126957 | NF-YA | 1.0mm | 2.302362205 |
| GRMZM2G113098 | GATA | 1.0mm | 2.285059978 |
| GRMZM2G151542 | ERF | 1.0mm | 2.283093054 |
| GRMZM2G103647 | bZIP | 1.0mm | 2.26869092 |
| GRMZM2G087741 | TALE | 1.0mm | 2.25413403 |
| GRMZM2G027333 | C2H2 | 1.0mm | 2.239792131 |
| GRMZM2G164591 | ERF | 1.0mm | 2.235989717 |
| GRMZM2G098904 | bZIP | 1.0mm | 2.223463687 |
| GRMZM2G172621 | B3 | 1.0mm | 2.221378976 |
| GRMZM2G141219 | AP2 | 1.0mm | 2.205665025 |
| GRMZM2G024851 | bZIP | 1.0mm | 2.196601942 |
| GRMZM5G812774 | BES1 | 1.0mm | 2.193208593 |

| | | | |
|---|---|---|---|
| GRMZM2G088309 | YABBY | 1.0mm | 2.191432396 |
| GRMZM2G142962 | HD-ZIP | 1.0mm | 2.181132075 |
| GRMZM2G049159 | GRAS | 1.0mm | 2.167137809 |
| GRMZM2G404973 | GATA | 1.0mm | 2.166331321 |
| GRMZM2G136266 | bZIP | 1.0mm | 2.164710485 |
| GRMZM2G306935 | C3H | 1.0mm | 2.152099105 |
| GRMZM2G027563 | bHLH | 1.0mm | 2.142410016 |
| GRMZM2G160687 | MIKC | 1.0mm | 2.14229249 |
| GRMZM2G097059 | MIKC | 1.0mm | 2.139388343 |
| GRMZM2G172657 | GRAS | 1.0mm | 2.135502324 |
| GRMZM2G379179 | Trihelix | 1.0mm | 2.131596695 |
| GRMZM2G310368 | ERF | 1.0mm | 2.125984252 |
| GRMZM2G069082 | ERF | 1.0mm | 2.123264312 |
| GRMZM2G082318 | GeBP | 1.0mm | 2.115752829 |
| GRMZM2G027976 | bZIP | 1.0mm | 2.111402359 |
| GRMZM2G086277 | C2H2 | 1.0mm | 2.101631117 |
| GRMZM2G163200 | GATA | 1.0mm | 2.083616918 |
| GRMZM2G093305 | C2H2 | 1.0mm | 2.078774617 |
| GRMZM2G087955 | MYB | 1.0mm | 2.076335878 |
| GRMZM2G093305 | C2H2 | 1.0mm | 2.075077399 |
| GRMZM2G030710 | ARF | 1.0mm | 2.071456462 |
| GRMZM2G158162 | B3 | 1.0mm | 2.062742223 |
| GRMZM2G531738 | MYB_related | 1.0mm | 2.061936495 |
| GRMZM2G102845 | ARF | 1.0mm | 2.061165049 |
| GRMZM2G162739 | NAC | 1.0mm | 2.059100742 |
| GRMZM2G032336 | CAMTA | 1.0mm | 2.057898187 |
| GRMZM2G010669 | MIKC | 1.0mm | 2.056254626 |
| GRMZM5G829103 | NF-YA | 1.0mm | 2.043197937 |
| GRMZM2G129428 | C2H2 | 1.0mm | 2.039702233 |
| GRMZM2G148723 | bHLH | 1.0mm | 2.022844688 |
| GRMZM2G479110 | ARR-B | 1.0mm | 2.021772939 |
| GRMZM2G151763 | WRKY | 1.0mm | 2.021327014 |
| GRMZM2G320827 | Trihelix | 1.0mm | 2.013457427 |

**Transcription factors decreased in expression**

| Gene ID | Family | Stage | Relative intensity compared to previous stage |
|---|---|---|---|
| GRMZM2G005624 | HD-ZIP | 0.25mm | 0.159622444 |
| GRMZM2G087741 | TALE | 0.25mm | 0.26545916 |
| GRMZM2G529859 | YABBY | 0.25mm | 0.283557919 |
| GRMZM2G017087 | TALE | 0.25mm | 0.28549975 |
| GRMZM2G160565 | MIKC | 0.25mm | 0.289216096 |
| GRMZM2G134279 | MYB | 0.25mm | 0.326057453 |
| GRMZM2G091201 | MYB_related | 0.25mm | 0.335834516 |
| GRMZM2G003514 | MIKC | 0.25mm | 0.348528015 |
| GRMZM2G074543 | YABBY | 0.25mm | 0.371785078 |
| GRMZM2G120320 | WRKY | 0.25mm | 0.387650872 |
| GRMZM2G072582 | MIKC | 0.25mm | 0.403132729 |

| | | | |
|---|---|---|---|
| GRMZM2G353076 | ZF-HD | 0.25mm | 0.414042553 |
| GRMZM2G005353 | YABBY | 0.25mm | 0.41617357 |
| GRMZM2G028151 | AP2 | 0.25mm | 0.419765446 |
| GRMZM2G068973 | NAC | 0.25mm | 0.422173145 |
| GRMZM2G108865 | Dof | 0.25mm | 0.435992707 |
| GRMZM2G054795 | YABBY | 0.25mm | 0.455377574 |
| GRMZM2G159431 | TALE | 0.25mm | 0.460368641 |
| GRMZM2G162739 | NAC | 0.25mm | 0.462047961 |
| GRMZM2G097349 | HD-ZIP | 0.25mm | 0.462427746 |
| GRMZM2G169654 | RAV | 0.25mm | 0.468571761 |
| GRMZM2G099522 | MIKC | 0.25mm | 0.48069615 |
| GRMZM2G177693 | C2H2 | 0.25mm | 0.48503937 |
| GRMZM2G033570 | EIL | 0.25mm | 0.486866792 |
| GRMZM2G430522 | NAC | 0.25mm | 0.492150706 |
| GRMZM2G159500 | NAC | 0.25mm | 0.493050133 |
| GRMZM2G030458 | GeBP | 0.4mm | TURN OFF |
| GRMZM2G171468 | G2-like | 0.4mm | TURN OFF |
| GRMZM2G124540 | G2-like | 0.4mm | TURN OFF |
| GRMZM2G159119 | G2-like | 0.4mm | TURN OFF |
| GRMZM2G018254 | GRAS | 0.4mm | TURN OFF |
| GRMZM2G169580 | Trihelix | 0.4mm | TURN OFF |
| GRMZM2G005624 | HD-ZIP | 0.4mm | TURN OFF |
| GRMZM2G003514 | MIKC | 0.4mm | TURN OFF |
| GRMZM2G529859 | YABBY | 0.4mm | 0.274272016 |
| GRMZM2G069082 | ERF | 0.4mm | 0.325882688 |
| GRMZM2G160565 | MIKC | 0.4mm | 0.387619323 |
| GRMZM2G124037 | ERF | 0.4mm | 0.440029077 |
| GRMZM2G087741 | TALE | 0.4mm | 0.455888744 |
| GRMZM2G134279 | MYB | 0.4mm | 0.472077697 |
| GRMZM2G068973 | NAC | 0.4mm | 0.480435237 |
| GRMZM2G017087 | TALE | 0.4mm | 0.481589714 |
| GRMZM2G119823 | bHLH | 0.7mm | TURN OFF |
| GRMZM2G136887 | MYB_related | 0.7mm | TURN OFF |
| GRMZM2G080731 | bZIP | 0.7mm | TURN OFF |
| GRMZM2G162481 | WOX | 0.7mm | TURN OFF |
| GRMZM2G307119 | ERF | 0.7mm | TURN OFF |
| GRMZM2G099319 | TALE | 0.7mm | TURN OFF |
| GRMZM2G145041 | MYB_related | 0.7mm | TURN OFF |
| GRMZM2G370863 | ZF-HD | 0.7mm | TURN OFF |
| GRMZM2G145579 | bHLH | 0.7mm | 0.154879494 |
| GRMZM5G829103 | NF-YA | 0.7mm | 0.225928623 |
| GRMZM5G805685 | B3 | 0.7mm | 0.262887847 |
| GRMZM2G169654 | RAV | 0.7mm | 0.33937687 |
| GRMZM2G158328 | WRKY | 0.7mm | 0.354982818 |
| GRMZM2G159500 | NAC | 0.7mm | 0.363272311 |
| GRMZM2G180190 | LFY | 0.7mm | 0.365514392 |
| GRMZM2G116557 | ARF | 0.7mm | 0.378880739 |
| GRMZM2G061408 | WRKY | 0.7mm | 0.393756129 |
| GRMZM2G045431 | bHLH | 0.7mm | 0.400946814 |

| | | | |
|---|---|---|---|
| GRMZM2G060216 | bZIP | 0.7mm | 0.401126485 |
| GRMZM2G027298 | C3H | 0.7mm | 0.404302671 |
| GRMZM2G386163 | NAC | 0.7mm | 0.412713472 |
| GRMZM5G803308 | MYB | 0.7mm | 0.413376459 |
| GRMZM2G060216 | bZIP | 0.7mm | 0.416708271 |
| GRMZM2G127857 | MYB | 0.7mm | 0.417561592 |
| GRMZM2G004957 | HD-ZIP | 0.7mm | 0.417779898 |
| GRMZM2G310368 | ERF | 0.7mm | 0.423335745 |
| GRMZM5G882527 | bHLH | 0.7mm | 0.423863162 |
| GRMZM2G120320 | WRKY | 0.7mm | 0.431205441 |
| GRMZM2G098813 | LFY | 0.7mm | 0.447590661 |
| GRMZM2G123900 | Dof | 0.7mm | 0.448290184 |
| GRMZM2G097683 | SRS | 0.7mm | 0.460297507 |
| GRMZM2G174284 | bZIP | 0.7mm | 0.463846385 |
| GRMZM2G139963 | HD-ZIP | 0.7mm | 0.468666667 |
| GRMZM2G133331 | bZIP | 0.7mm | 0.469170579 |
| GRMZM2G301089 | bHLH | 0.7mm | 0.483369883 |
| GRMZM2G178741 | HD-ZIP | 0.7mm | 0.487521151 |
| GRMZM2G453001 | STAT | 0.7mm | 0.494759473 |
| GRMZM2G134545 | Dof | 0.7mm | 0.498534799 |
| GRMZM2G065374 | bHLH | 1.0mm | TURN OFF |
| GRMZM2G327189 | Dof | 1.0mm | TURN OFF |
| GRMZM2G153333 | GRAS | 1.0mm | TURN OFF |
| GRMZM2G002915 | HD-ZIP | 1.0mm | TURN OFF |
| GRMZM2G470307 | MYB | 1.0mm | TURN OFF |
| GRMZM2G312201 | NAC | 1.0mm | TURN OFF |
| GRMZM2G034563 | G2-like | 1.0mm | 0.043339578 |
| GRMZM2G102218 | YABBY | 1.0mm | 0.090284694 |
| GRMZM2G096709 | GRF | 1.0mm | 0.105003817 |
| GRMZM2G100146 | C2H2 | 1.0mm | 0.122588993 |
| GRMZM2G338259 | ARF | 1.0mm | 0.129574468 |
| GRMZM2G393433 | NAC | 1.0mm | 0.14172688 |
| GRMZM2G378580 | ARF | 1.0mm | 0.188027274 |
| GRMZM5G804893 | NF-YB | 1.0mm | 0.189824773 |
| GRMZM2G113779 | SBP | 1.0mm | 0.206116839 |
| GRMZM2G069146 | ERF | 1.0mm | 0.214033019 |
| GRMZM5G894234 | NAC | 1.0mm | 0.228860936 |
| GRMZM2G113127 | NF-YC | 1.0mm | 0.24367077 |
| GRMZM2G392516 | GeBP | 1.0mm | 0.25477707 |
| GRMZM5G887286 | C2H2 | 1.0mm | 0.259254482 |
| GRMZM2G006493 | C3H | 1.0mm | 0.264980491 |
| GRMZM2G041223 | GRF | 1.0mm | 0.280744068 |
| GRMZM2G076272 | TALE | 1.0mm | 0.284058522 |
| GRMZM2G352159 | ARF | 1.0mm | 0.286424355 |
| GRMZM2G050939 | C2H2 | 1.0mm | 0.29821718 |
| GRMZM2G159357 | HB-other | 1.0mm | 0.304162725 |
| GRMZM2G102514 | BES1 | 1.0mm | 0.315651626 |
| GRMZM2G033413 | bZIP | 1.0mm | 0.317846656 |
| GRMZM2G065506 | GeBP | 1.0mm | 0.318018665 |

| | | | |
|---|---|---|---|
| GRMZM2G039828 | bZIP | 1.0mm | 0.318021631 |
| GRMZM2G701689 | CPP | 1.0mm | 0.319008759 |
| GRMZM2G019446 | bZIP | 1.0mm | 0.320781313 |
| GRMZM2G133169 | GRAS | 1.0mm | 0.324274471 |
| GRMZM2G123140 | HD-ZIP | 1.0mm | 0.33064776 |
| GRMZM2G154641 | TALE | 1.0mm | 0.331354972 |
| GRMZM2G129147 | GRF | 1.0mm | 0.338819415 |
| GRMZM2G069408 | bHLH | 1.0mm | 0.343832021 |
| GRMZM2G031120 | NAC | 1.0mm | 0.34404708 |
| GRMZM2G053503 | ERF | 1.0mm | 0.347585662 |
| GRMZM2G702026 | ARF | 1.0mm | 0.353558926 |
| GRMZM2G156016 | VOZ | 1.0mm | 0.35942492 |
| GRMZM2G120740 | C3H | 1.0mm | 0.366255144 |
| GRMZM2G479110 | ARR-B | 1.0mm | 0.369612069 |
| GRMZM2G109987 | HD-ZIP | 1.0mm | 0.369684607 |
| GRMZM2G048450 | WRKY | 1.0mm | 0.373132346 |
| GRMZM2G174917 | ERF | 1.0mm | 0.380319497 |
| GRMZM2G312201 | NAC | 1.0mm | 0.383005255 |
| GRMZM2G126957 | NF-YA | 1.0mm | 0.38499385 |
| GRMZM2G003944 | TCP | 1.0mm | 0.386550308 |
| GRMZM2G064541 | NAC | 1.0mm | 0.386563416 |
| GRMZM2G022213 | C2H2 | 1.0mm | 0.388605442 |
| GRMZM2G173425 | LSD | 1.0mm | 0.389802875 |
| GRMZM2G096759 | MYB_related | 1.0mm | 0.391697493 |
| GRMZM2G061487 | ERF | 1.0mm | 0.392430279 |
| GRMZM2G055204 | ERF | 1.0mm | 0.392656808 |
| GRMZM2G341747 | CAMTA | 1.0mm | 0.395641646 |
| GRMZM2G089406 | MYB_related | 1.0mm | 0.398830007 |
| GRMZM2G479110 | ARR-B | 1.0mm | 0.401297947 |
| GRMZM2G431157 | C2H2 | 1.0mm | 0.404473439 |
| GRMZM2G456568 | NAC | 1.0mm | 0.405565979 |
| GRMZM2G174240 | GeBP | 1.0mm | 0.406111536 |
| GRMZM2G431157 | C2H2 | 1.0mm | 0.406652807 |
| GRMZM2G370777 | MIKC | 1.0mm | 0.413241293 |
| GRMZM2G389567 | bHLH | 1.0mm | 0.415374241 |
| GRMZM2G125777 | NAC | 1.0mm | 0.417694933 |
| GRMZM2G031001 | NAC | 1.0mm | 0.417739483 |
| GRMZM2G071907 | WRKY | 1.0mm | 0.417923959 |
| GRMZM2G009530 | GATA | 1.0mm | 0.419946174 |
| GRMZM2G174776 | NF-YC | 1.0mm | 0.421550355 |
| GRMZM2G151407 | WRKY | 1.0mm | 0.421917808 |
| GRMZM5G805026 | WOX | 1.0mm | 0.422074604 |
| GRMZM2G396451 | GATA | 1.0mm | 0.426806613 |
| GRMZM2G114461 | FAR1 | 1.0mm | 0.427480916 |
| GRMZM2G050590 | E2F/DP | 1.0mm | 0.427574171 |
| GRMZM2G164735 | BBR-BPC | 1.0mm | 0.431788653 |
| GRMZM2G064197 | G2-like | 1.0mm | 0.438966877 |
| GRMZM2G379608 | NAC | 1.0mm | 0.442261104 |
| GRMZM2G416652 | MYB | 1.0mm | 0.447083775 |

| | | | |
|---|---|---|---|
| GRMZM2G126018 | SBP | 1.0mm | 0.449616648 |
| GRMZM2G101499 | SBP | 1.0mm | 0.450732137 |
| GRMZM2G013657 | AP2 | 1.0mm | 0.450952717 |
| GRMZM2G125777 | NAC | 1.0mm | 0.451251841 |
| GRMZM2G003304 | HD-ZIP | 1.0mm | 0.451289398 |
| GRMZM2G070034 | MIKC | 1.0mm | 0.451731375 |
| GRMZM2G339848 | HB-other | 1.0mm | 0.453190676 |
| GRMZM2G169316 | MYB | 1.0mm | 0.454652256 |
| GRMZM2G033138 | HB-other | 1.0mm | 0.456330081 |
| GRMZM2G030744 | bHLH | 1.0mm | 0.459872458 |
| GRMZM2G095598 | CO-like | 1.0mm | 0.460066393 |
| GRMZM2G338259 | ARF | 1.0mm | 0.463735361 |
| GRMZM2G403620 | MYB | 1.0mm | 0.465581051 |
| GRMZM2G032336 | CAMTA | 1.0mm | 0.465858328 |
| GRMZM2G179049 | NAC | 1.0mm | 0.4660523 |
| GRMZM2G092091 | bHLH | 1.0mm | 0.469780609 |
| GRMZM2G028980 | ARF | 1.0mm | 0.470108696 |
| GRMZM2G130854 | WRKY | 1.0mm | 0.472826087 |
| GRMZM2G081812 | C2H2 | 1.0mm | 0.475670308 |
| GRMZM2G060216 | bZIP | 1.0mm | 0.476190476 |
| GRMZM2G006871 | GeBP | 1.0mm | 0.477103301 |
| GRMZM2G139815 | WRKY | 1.0mm | 0.479763861 |
| GRMZM2G067743 | GRF | 1.0mm | 0.481185031 |
| GRMZM2G150262 | C3H | 1.0mm | 0.483062331 |
| GRMZM2G129428 | C2H2 | 1.0mm | 0.483926423 |
| GRMZM2G025685 | HSF | 1.0mm | 0.489446388 |
| GRMZM2G061906 | bHLH | 1.0mm | 0.490433577 |
| GRMZM2G125239 | GeBP | 1.0mm | 0.493167325 |
| GRMZM2G384528 | NF-YB | 1.0mm | 0.493708609 |
| GRMZM2G069009 | C3H | 1.0mm | 0.495503999 |
| GRMZM2G095239 | MYB_related | 1.0mm | 0.49656035 |

Transcription factors that are turned ON/OFF or UP/DOWN-regulated by at least two fold compared to previous stage. Gene identifiers, gene families, stages when differential transcript accumulation occurs and relative intensity of transcripts compared to prior stages are listed.
