# Supplementary material for: Transcriptomes and Proteomes Define Gene Expression Progression in Pre-meiotic Maize Anthers
Source: G3 (Bethesda). 2014 Jun 1;4(6):993–1010. doi: 10.1534/g3.113.009738 (PMC4065268; doi:10.1534/g3.113.009738)
Supplement: Supporting Information [file supp_4.6.993_TableS7.pdf]

Table S7 Meiotic gene expression at five stages of early anther development.

MaizeGdbProteinID	MaizeGdbProteinName	0.15mm	0.25mm	0.4mm	0.7mm	1.0mm
AC198518.3_FG002	Sterile alpha motif (SAM) domain-containing protein	ON	ON	ON	ON	ON
AC207628.4_FG005	Porin/voltage-dependent anion-selective channel protein, alpha amylase activity	OFF	ON	ON	ON	ON
AC231745.1_FGT004	Protein-tyrosine sulfotransferase†	ON	ON	ON	ON	ON
GRMZM2G000397	Calcium-binding EF hand family protein	ON	ON	ON	ON	ON
GRMZM2G001803	ZIP metal ion transporter family	ON	ON	ON	ON	ON
GRMZM2G002765	UBA/THIF-type NAD/FAD binding fold	ON	ON	ON	ON	ON
GRMZM2G004455	RNA recognition motif 2	ON	ON	ON	ON	ON
GRMZM2G005036	Mitochondrial processing peptidase, alpha subunit	ON	ON	ON	ON	ON
GRMZM2G005374	Splicing factor 3b, subunit 4	ON	ON	ON	ON	ON
GRMZM2G006452	RecF/RecN/SMC protein, N-terminal	ON	ON	ON	ON	ON
GRMZM2G006452	RecF/RecN/SMC protein, N-terminal	ON	ON	ON	ON	ON
GRMZM2G006452	Tropomyosin	ON	ON	ON	ON	ON
GRMZM2G006452	RecF/RecN/SMC protein, N-terminal	ON	ON	ON	ON	ON
GRMZM2G006468	BBD: bifunctional nuclease in wound defense	ON	ON	ON	ON	ON
GRMZM2G007300	Ubiquitin carrier protein	ON	ON	ON	ON	ON
GRMZM2G008226	Trehalose-6-phosphate synthase	ON	ON	OFF	ON	OFF
GRMZM2G011078	Secretory carrier membrane protein, SC3, transport	ON	ON	ON	ON	ON
GRMZM2G012030	Vinculin/alpha-catenin	ON	ON	ON	ON	ON
GRMZM2G016602	protein recA	ON	ON	ON	ON	ON
GRMZM2G019596	Vamp/synaptobrevin-associated protein 27-2; major sperm protein	ON	ON	ON	ON	ON
GRMZM2G019596	Vamp/synaptobrevin-associated protein 27-2; major sperm protein	ON	ON	ON	ON	ON
GRMZM2G020974	DNA breaking-rejoining enzyme, catalytic core	ON	ON	ON	ON	ON
GRMZM2G021270	hypothetical protein LOC100193866	ON	ON	ON	ON	ON
GRMZM2G021270	DNA topoisomerase, type IIA, subunit A or C-termin	ON	ON	ON	ON	ON
GRMZM2G024739	Cryptochrome/DNA photolyase, class 1 conserved sit	ON	ON	ON	ON	ON
GRMZM2G028369	Chorismate mutase precursor	ON	ON	ON	ON	ON
GRMZM2G028640	Cupin superfamily enzyme	ON	ON	ON	ON	ON
GRMZM2G028763	Membrane related protein-like	ON	ON	ON	ON	ON
GRMZM2G029385	Chaperone (DnaJ); mitochondrial import inner membrane translocase subunit TIM14	ON	ON	ON	ON	ON
GRMZM2G030523	Proliferating cell nuclear antigen, PCNA	ON	ON	ON	ON	ON
GRMZM2G032003	UTP--glucose-1-phosphate uridylyltransferase†	ON	ON	ON	ON	ON
GRMZM2G032562	SCF ubiquitin ligase, Skp1	ON	ON	ON	ON	ON

	component; SKP1-like protein 1B (mediates cell cycle)					
GRMZM2G034631	Tyrosylprotein sulfotransferase	ON	ON	ON	ON	ON
GRMZM2G035996	Shaggy kinase homolog Fragment	ON	ON	ON	ON	OFF
GRMZM2G036765	Aspartate decarboxylase-like fold	ON	ON	ON	ON	ON
GRMZM2G039094	MATH domain containing protein	ON	ON	ON	ON	ON
GRMZM2G041418	NADH-dehydrogenase (ubiquinone),	OFF	ON	OFF	ON	ON
GRMZM2G042477	Monoglyceride lipase isoform 2-like, partial (90%)	ON	ON	ON	ON	ON
GRMZM2G043509	Endonuclease/Exonuclease/phosphatase family; DNaseI-like	ON	ON	ON	ON	ON
GRMZM2G044011	eRF1 domain 3	ON	ON	ON	ON	ON
GRMZM2G046055	Histone H2A	ON	ON	ON	ON	ON
GRMZM2G047204	Peptidyl-prolyl glycoprotein; histidine kinase	ON	ON	ON	ON	ON
GRMZM2G050329	Major Facilitator Superfamily with SPX (SYG1/Pho81/XPR1)	ON	ON	ON	ON	ON
GRMZM2G050684	Predicted membrane protein, contains two CBS domains	ON	ON	ON	ON	ON
GRMZM2G050684	CBS domain containing protein	ON	ON	ON	ON	ON
GRMZM2G052403	hypothetical protein LOC100383700	ON	ON	ON	ON	ON
GRMZM2G054115	Alliinase EGF-like domain; Pyridoxal phosphate (PLP)-dependent transferases †	ON	ON	ON	ON	ON
GRMZM2G055807	Restriction endonuclease, type I, R subunit/Type I	ON	ON	ON	ON	ON
GRMZM2G055807	DNA repair and transcription factor XPB1	ON	ON	ON	ON	ON
GRMZM2G056075	DNA mismatch repair protein MSH2 (MUS1)	ON	ON	ON	ON	ON
GRMZM2G057652	Protein of unknown function DUF1751, integral memb	ON	ON	OFF	OFF	ON
GRMZM2G058954	ATPase, AAA+ type, core	ON	ON	ON	ON	ON
GRMZM2G058954	DNA repair protein RAD51/RHP55, single stranded DNA repair	ON	ON	ON	ON	ON
GRMZM2G060394	SKP1-like protein 1A	ON	ON	OFF	ON	ON
GRMZM2G061023	RuvA domain 2-like	ON	ON	ON	ON	ON
GRMZM2G062761	MAP kinase, conserved site	ON	ON	ON	ON	ON
GRMZM2G062914	Serine/threonine protein kinase-related	ON	ON	ON	ON	ON
GRMZM2G063316	SET domain-containing protein,Rubisco methyltransferase family protein	ON	ON	ON	ON	ON
GRMZM2G063961	Serine/threonine-protein kinase SAPK4	ON	ON	ON	ON	ON
GRMZM2G064868	Putative 5-3 exonuclease	ON	ON	ON	ON	ON
GRMZM2G064868	Zinc finger, CCHC-type	ON	ON	ON	ON	ON
GRMZM2G070047	Ubiquitin-conjugating enzyme/RWD-like	OFF	OFF	OFF	OFF	OFF
GRMZM2G070639	Kinesin-like protein	OFF	OFF	OFF	OFF	ON
GRMZM2G071304	ATP dependent DNA ligase, central	ON	ON	ON	ON	ON
GRMZM2G071304	ATP dependent DNA ligase,	ON	ON	ON	ON	ON

	central					
GRMZM2G071630	Glyceraldehyde 3-phosphate dehydrogenase (GAPC3)	ON	ON	ON	ON	ON
GRMZM2G072088	Unknown function, localized to plasma membrane	ON	ON	OFF	ON	ON
GRMZM2G074082	Shugoshin, N-terminal	ON	ON	OFF	ON	ON
GRMZM2G074818	Rad21/Rec8 like protein, N-terminal	ON	ON	ON	ON	ON
GRMZM2G074818	Rad21/Rec8 like protein, N-terminal	ON	ON	ON	ON	ON
GRMZM2G074818	Rad21/Rec8 like protein, N-terminal	ON	ON	ON	ON	ON
GRMZM2G077823	Arginine:serine-rich splicing factor SC35	ON	ON	ON	ON	ON
GRMZM2G079613	Tetratricopeptide TPR-1	ON	ON	ON	ON	ON
GRMZM2G082214	Phosphoribosyl-AMP cyclohydrolase	ON	ON	ON	ON	ON
GRMZM2G083394	DCD domain protein - involved in development and cell death during HR response	ON	ON	ON	ON	ON
GRMZM2G083475	Prefoldin	ON	ON	ON	ON	ON
GRMZM2G083975	Splicing factor RSZ33 (RSZ33)	ON	ON	ON	ON	ON
GRMZM2G084762	DNA repair and recombination, RecA-like	ON	ON	ON	ON	ON
GRMZM2G088162	hypothetical protein LOC100276374	ON	ON	ON	ON	ON
GRMZM2G090152	Core-2/I-Branching enzyme, beta-1,6-N-acetylglucosaminyltransferase, role in salt stress.	ON	ON	ON	ON	ON
GRMZM2G090262	C-terminal phosphatase-like 4; NLI interacting factor-like phosphatase	ON	ON	ON	ON	ON
GRMZM2G091656	GRAS domain family, RGA-like 2	ON	ON	ON	ON	ON
GRMZM2G092232	Kinesin, motor region	ON	ON	ON	ON	ON
GRMZM2G093119	Rad21/Rec8 like protein, N-terminal	ON	ON	ON	ON	ON
GRMZM2G093623	DNA mismatch repair protein MutS, C-terminal	ON	ON	OFF	ON	ON
GRMZM2G096070	WD40 repeat-like	ON	ON	ON	ON	ON
GRMZM2G097605	Helicase-like, DEXD box c2 type	ON	ON	ON	ON	ON
GRMZM2G099080	SNX2b: sorting nexin 2B; phox domain containing protein	ON	ON	ON	ON	ON
GRMZM2G100103	Poor homologous synapsis 1 (PHS1) (meiosis chromosome pairing)	OFF	OFF	OFF	OFF	ON
GRMZM2G101613	Cyclin-dependent kinase inhibitor	OFF	ON	ON	ON	ON
GRMZM2G102088	Serine/threonine protein kinase-related	ON	ON	ON	ON	ON
GRMZM2G103287	Apospory-associated proteion aldose 1-epimerase	ON	ON	ON	ON	ON
GRMZM2G103287	Apospory-associated protein; aldose 1-epimerase	ON	ON	ON	ON	ON
GRMZM2G105250	AGO18a; Translation initiation factor 2C (eIF-2C)	ON	ON	ON	ON	OFF
GRMZM2G105387	MADS32; transcription factor; homolog of AtAGAMOUS-like 12	OFF	OFF	OFF	OFF	OFF

GRMZM2G108712	Proliferating cell nuclear antigen, PCNA	ON	ON	ON	ON	ON
GRMZM2G109383	Phosphoglucomutase, cytoplasmic	ON	ON	ON	ON	ON
GRMZM2G109383	Phosphoglucomutase, cytoplasmic 1 (PGM 1)(EC 5.4.2), phosphomannomutase	ON	ON	ON	ON	ON
GRMZM2G109496	Serine/threonine protein phosphatase	ON	ON	ON	ON	ON
GRMZM2G109618	DMC1 protein type B, DNA repair during meiotic recombination	OFF	OFF	OFF	OFF	OFF
GRMZM2G110212	DNA mismatch repair protein MutS-like, N-terminal	ON	ON	ON	ON	ON
GRMZM2G111436	Predicted 3'-5' exonuclease, Werner syndrome DNA helicase, nucleosidase	ON	ON	ON	ON	ON
GRMZM2G113228	Endonuclease III, 4Fe4S cluster, base excision repair	ON	ON	ON	ON	ON
GRMZM2G113967	NAF	ON	ON	OFF	ON	ON
GRMZM2G115013	RPA1a, replication protein required for meiotic DNA repair	ON	ON	ON	ON	ON
GRMZM2G115504	alpha / beta esterase; thioesterase; hydrolase	ON	ON	ON	ON	ON
GRMZM2G116243	1-acylglycerol-3-phosphate O-acyltransferase	ON	ON	ON	ON	ON
GRMZM2G116427	Metal ion binding; mitochondrial protein	ON	ON	ON	ON	ON
GRMZM2G119886	Arf GTPase activating protein	ON	ON	ON	ON	ON
GRMZM2G121210	DNA topoisomerase, type IIA, central	ON	ON	ON	ON	ON
GRMZM2G121262	Helix-hairpin-helix motif, class 2	ON	ON	ON	ON	ON
GRMZM2G121312	Leucine-rich repeat, typical subtype	ON	ON	ON	ON	ON
GRMZM2G121543	DNA repair protein RAD51	ON	ON	ON	ON	ON
GRMZM2G122306	Nucleic acid-binding, OB-fold-like	ON	ON	ON	ON	ON
GRMZM2G122965	P-loop ATP hydrolase, CH (Calponin Homology) domain, KINESIN motor domain containing	OFF	OFF	OFF	OFF	ON
GRMZM2G123776	Thioredoxin or redox protein	ON	ON	ON	ON	ON
GRMZM2G124365	Chorismate mutase	ON	ON	OFF	ON	ON
GRMZM2G124691	Cyclin	OFF	ON	OFF	ON	ON
GRMZM2G124718	Poly(ADP-ribose) polymerase, catalytic region	ON	ON	ON	ON	ON
GRMZM2G127893	hypothetical protein LOC100217125	ON	ON	OFF	ON	ON
GRMZM2G128771	GTPase Rab6/YPT6/Ryh1, small G protein superfamily	ON	ON	ON	ON	ON
GRMZM2G129175	Uncharacterized, contains transmembrane domain	ON	ON	ON	ON	ON
GRMZM2G129913	Spo11/DNA topoisomerase VI, subunit A	ON	ON	ON	ON	ON
GRMZM2G131443	Aldehyde dehydrogenase, conserved site	ON	ON	ON	ON	ON
GRMZM2G133006	MATE efflux family protein	ON	OFF	OFF	OFF	ON
GRMZM2G133048	Phosphatidate cytidylyltransferase†	ON	ON	ON	ON	ON
GRMZM2G133952	DNA mismatch repair, conserved	ON	ON	ON	ON	ON

	site					
GRMZM2G134502	Emp24/gp25L/p24 membrane trafficking; phospholipid binding; annexin	ON	ON	ON	ON	ON
GRMZM2G134708	Monodehydroascorbate/ferredoxin reductase	ON	ON	ON	ON	ON
GRMZM2G135073	Ste20-like serine/threonine protein kinase	ON	ON	ON	ON	ON
GRMZM2G135654	Ribosomal protein L7Ae/L30e/S12e/Gadd45	ON	ON	ON	ON	ON
GRMZM2G136455	GTPase Rab1/YPT1, small G protein superfamily, and related GTP-binding proteins	ON	ON	ON	ON	ON
GRMZM2G136710	Predicted K+/H+-antiporter	OFF	ON	ON	ON	ON
GRMZM2G137968	DSB repair, DNA helicase - NHEJ (non homologous end joining) double stranded break	ON	ON	ON	ON	ON
GRMZM2G138566	Calcium binding atopy-related autoantigen 1 (LOC10	ON	ON	ON	ON	ON
GRMZM2G138907	GDP-mannose 3\'5\' epimerase; dehydratase	ON	ON	ON	ON	ON
GRMZM2G139031	8-oxoguanine DNA glycosylase, N-terminal	ON	ON	ON	ON	ON
GRMZM2G140994	Rhomboid domain containing 1	ON	ON	ON	ON	ON
GRMZM2G143211	WD40 repeat	ON	ON	ON	ON	ON
GRMZM2G143462	Ubiquitin-associated/translation elongation factor	ON	ON	ON	ON	ON
GRMZM2G143590	Synaptonemal complex central region protein ZYP1-1	ON	ON	ON	ON	ON
GRMZM2G145034	Splicing factor, SPF45	ON	ON	ON	ON	ON
GRMZM2G145085	Transcription initiation factor IIF, large subunit (RAP74)	ON	ON	ON	ON	ON
GRMZM2G146206	Triosephosphate isomerase	ON	ON	OFF	ON	ON
GRMZM2G146490	BRCT	ON	ON	ON	ON	ON
GRMZM2G148249	DEAD-like helicase, N-terminal	OFF	OFF	OFF	OFF	OFF
GRMZM2G149289	hypothetical protein LOC100383454	ON	ON	ON	ON	ON
GRMZM2G153760	Galactosyltransferases	ON	ON	ON	ON	ON
GRMZM2G153899	Mitochondrial K+-H+ exchange-related	ON	ON	ON	ON	ON
GRMZM2G155806	Pumilio-family RNA binding repeat†	OFF	OFF	OFF	OFF	OFF
GRMZM2G157269	Acetyl-CoA synthetase	OFF	ON	OFF	OFF	OFF
GRMZM2G157505	EGG APPARATUS-1 protein	ON	ON	ON	ON	ON
GRMZM2G157817	Rad51, C-terminal	OFF	OFF	OFF	OFF	OFF
GRMZM2G159034	VH1-interacting kinase; ankyrin repeat containing	ON	ON	ON	ON	ON
GRMZM2G159307	ATPase associated with various cellular activities	OFF	ON	OFF	ON	OFF
GRMZM2G159724	NADP+-dependent malic enzyme	ON	ON	ON	ON	ON
GRMZM2G162968	Heat shock protein	ON	ON	ON	ON	ON
GRMZM2G163514	Leafbladeless1-like	ON	ON	ON	ON	ON
GRMZM2G164470	RXW8 protein, Lipase, GDSL	ON	ON	ON	ON	ON
GRMZM2G166597	Alpha-D-phosphohexomutase, alpha/beta/alpha I, II	ON	OFF	OFF	ON	ON

GRMZM2G166658	Serine/threonine protein kinase-related	ON	ON	ON	ON	ON
GRMZM2G169089	Acyl-CoA:diacylglycerol acyltransferase 1	ON	ON	ON	ON	OFF
GRMZM2G169709	Phosphoethanolamine N-methyltransferase	ON	ON	ON	ON	ON
GRMZM2G169931	Nonsense-mediated mRNA decay NMD3 family protein	OFF	ON	ON	ON	ON
GRMZM2G173428	FOG: RRM domain, CID11, nucleic acid binding	OFF	ON	OFF	ON	ON
GRMZM2G174574	Acyl-CoA synthetase	OFF	OFF	OFF	ON	OFF
GRMZM2G175676	hypothetical protein LOC100384862	ON	ON	ON	ON	ON
GRMZM2G175676	RNA recognition motif, RNP-1	ON	ON	ON	ON	ON
GRMZM2G177461	Myo-inositol-1-phosphate synthase, inositol-3-phosphate synthase	ON	ON	ON	ON	ON
GRMZM2G179215	Ras GTPase	ON	ON	ON	ON	ON
GRMZM2G300786	SWI1 / DYAD involved in meiotic recombination	ON	ON	OFF	ON	ON
GRMZM2G301405	hypothetical protein LOC100192986	ON	ON	ON	ON	ON
GRMZM2G310739	3-glucanase	ON	ON	OFF	ON	ON
GRMZM2G315176	DNA/RNA helicase, DEAD/DEAH box type, N-terminal	ON	ON	ON	ON	ON
GRMZM2G315726	LAG1 longevity assurance homolog 3, ASC1	ON	ON	ON	ON	ON
GRMZM2G315726	ASC1, WD-domain, sphingolipid biosynthesis, resists fungal infection	ON	ON	ON	ON	ON
GRMZM2G315769	Sodium:dicarboxylate symporter	ON	ON	ON	ON	ON
GRMZM2G324390	Keratin, type I	ON	ON	ON	ON	ON
GRMZM2G324781	SNF2 helicase domain-containing protein; DNA repair protein RAD16	ON	ON	ON	ON	ON
GRMZM2G329710	EBNA-1, FAS1 domain	ON	ON	ON	ON	ON
GRMZM2G336761	Zinc finger, C3HC4 type (RING finger), U-box	ON	ON	ON	ON	ON
GRMZM2G342807	Permeases (Mycobacterium smegmatis str. MC2 155)	ON	ON	ON	ON	ON
GRMZM2G347717	UDP-glucuronic acid decarboxylase, 3-beta-hydroxy-Delta(5)-steroid dehydrogenase	ON	ON	OFF	ON	ON
GRMZM2G349996	Eukaryotic translation initiation factor 1A	ON	ON	ON	ON	ON
GRMZM2G351810	bHLH transcription factor	OFF	ON	OFF	ON	ON
GRMZM2G372180	Calponin-like actin-binding	ON	ON	ON	ON	ON
GRMZM2G390896	Protein kinase-like	ON	ON	OFF	ON	OFF
GRMZM2G408768	14-3-3 protein	ON	ON	OFF	ON	ON
GRMZM2G410710	Prohibitin, Mitochondrial prohibitin complex protein 1, membrane, PHB3	ON	ON	ON	ON	ON
GRMZM2G416019	Leucine-rich repeat	OFF	OFF	OFF	OFF	OFF
GRMZM2G416069	RecF/RecN/SMC protein, N-terminal	OFF	OFF	OFF	OFF	ON
GRMZM2G425986	Endonuclease III-like, iron-sulphur	ON	ON	ON	ON	ON

	cluster loop					
GRMZM2G432075	ZmAGO121, AtAGO6 and AtAGO4 homolog (involved in RdDM) Zwille:pinhead-like	ON	ON	ON	ON	ON
GRMZM2G447447	Protein kinase-like	ON	ON	ON	ON	ON
GRMZM2G456570	SMCs flexible hinge	ON	ON	ON	ON	ON
GRMZM2G456626	Inositol 1,3,4-trisphosphate 5/6-kinase family protein	ON	ON	ON	ON	ON
GRMZM2G457370	AGO18b; similar to ZLL:PNH	OFF	OFF	OFF	OFF	ON
GRMZM2G458974	Ribosomal protein S7e family	OFF	ON	OFF	ON	OFF
GRMZM2G459702	tRNA pseudouridine synthase D (TruD)†	ON	ON	ON	ON	ON
GRMZM2G464382	Exo70 exocyst complex subunit†(exocytosis)	OFF	OFF	OFF	ON	OFF
GRMZM2G465553	Transcription elongation factor, IIS	ON	ON	ON	ON	ON
GRMZM2G468479	hypothetical protein LOC100217142	ON	ON	ON	ON	ON
GRMZM2G470438	Zinc finger, GRF-type	ON	ON	ON	ON	ON
GRMZM2G474929	Selenium-binding protein-like	ON	ON	ON	ON	ON
GRMZM2G547542	Malonyl CoA-acyl carrier protein transacylase	ON	ON	ON	ON	ON
GRMZM2G563190	NADH-dehydrogenase (ubiquinone) - FAD, NADP, NADPH binding, cytoplasm	ON	ON	ON	ON	OFF
GRMZM2G588241	FOG: Predicted E3 ubiquitin ligase, RHC1A	ON	ON	ON	ON	ON
GRMZM2G700757	RecA family protein, NTP binding, DNA repair, ssDNA binding	ON	ON	ON	ON	ON
GRMZM5G802801	HSP70	ON	ON	ON	ON	ON
GRMZM5G806358	FACT complex subunit SPT16 (Facilitates transcription via Pol II)	ON	ON	ON	ON	ON
GRMZM5G828630	Alanine aminotransferase	ON	ON	ON	ON	ON
GRMZM5G831712	Poly [ADP-ribose] polymerase 1 (PARP-1)(EC 2.4.2.3	ON	ON	ON	ON	ON
GRMZM5G832378	Transcription initiation factor IIA gamma chain (TFIIA-gamma)	OFF	OFF	OFF	ON	ON
GRMZM5G841619	Tryptophan synthase alpha	ON	ON	ON	ON	ON
GRMZM5G842071	Laccase 1, complete (oxygen binding - quinone associated)	OFF	OFF	OFF	OFF	OFF
GRMZM5G856297	Meiotic recombination protein	OFF	OFF	OFF	OFF	OFF
GRMZM5G870959	somatic embryogenesis receptor-like kinase1	ON	ON	ON	ON	ON
GRMZM5G878823	Kinesin 1-like	ON	ON	ON	ON	ON
GRMZM5G889299	UTP--glucose-1-phosphate uridylyltransferase	ON	ON	ON	ON	ON
GRMZM5G890815	PAP/OAS1 substrate-binding domain superfamily, nucleotidyltransferase	OFF	OFF	OFF	ON	ON
GRMZM5G892926	Not determined - may be psuedogene	ON	ON	ON	ON	ON
GRMZM5G899800	hypothetical protein LOC100272324	ON	ON	ON	ON	ON

Expression patterns of genes classified as meiotic genes, including known genes involved in maize meiosis, maize homologs of meiotic genes in other flowering plants, and 222 annotated genes that are all mis-regulated in *ameiotic1* anthers were scored. Gene identifiers, Gene Ontology and their expressions (ON or OFF) are listed.
